# Stepwise Modulation of Bridged Single‐Benzene‐Based Fluorophores for Materials Science

**DOI:** 10.1002/chem.202404263

**Published:** 2025-01-22

**Authors:** Alexander Huber, Laura Schmidt, Tim Gatz, Jana Bublitz, Tobias Rex, Sidharth Thulaseedharen Nair Sailaja, Elisabeth Verheggen, Lea Höfmann, Christoph Wölper, Cristian A. Strassert, Shirley K. Knauer, Jens Voskuhl

**Affiliations:** ^1^ Faculty of Chemistry (Organic Chemistry) CENIDE and Center of Medical Biotechnology (ZMB) University of Duisburg-Essen Universitätsstraße 7 45141 Essen Germany; ^2^ Department of Molecular Biology II Center of Medical Biotechnology (ZMB) University of Duisburg-Essen Universitätsstraße 2 45141 Essen Germany; ^3^ Institut für Anorganische und Analytische Chemie CeNTech, CiMIC, SoN Universität Münster Heisenbergstraße 11 48149 Münster Germany; ^4^ Faculty of Chemistry (Inorganic Chemistry) University of Duisburg-Essen Universitätsstraße 7 45141 Essen Germany

**Keywords:** Single-Benzene Based Fluorophores (SBBF), Solution- and Solid-State Emission (SSSE), White light emission (WLE), Stereolithography (SLA), Structure-property relationship

## Abstract

In recent years, researchers studying fluorogenic samples have steadily shifted from using large, expensive, poorly soluble fluorophores with complex synthetic sequences to smaller, simpler π scaffolds with low molecular weight. This research article presents an in‐depth study of the photophysical properties of five bridged single‐benzene‐based fluorophores (SBBFs) investigated for their solution and solid‐state emission (SSSE) properties. The compounds **O_4_**, **N_1_O_3_**, **N_2_O_2_**, **N_3_O_1_**, and **N_4_** are derived from a central terephthalonitrile core and vary in the amount of oxygen and nitrogen bridging atoms. These minimalized emitters show full‐color tunable emission properties and exhibit moderate‐to‐high photoluminescence quantum yield values reaching up to 0.78 in dimethyl sulfoxide (DMSO). In addition to demonstrating excellent compatibility in poly(methyl methacrylate) (PMMA) films and additive manufacturing using stereolithography (SLA), white light emission was achieved in both solution and 3D‐printed materials by controlling the mixing ratio of the compounds. Employing density‐functional theory (DFT), well‐correlating theoretical absorption and emission wavelengths were calculated as average values of the different possible conformers. Furthermore, cellular internalization of the substances was accomplished using Pluronic^®^ F‐127 nanoparticles. Overall, this study emphasizes the remarkable properties of single‐benzene‐based emitters, showcasing their accessibility and potential applications in biomedical fields and materials science.

## Graphical abstract

A library consisting of five single‐benzene‐based fluorophores was designed. Changing the bridging atoms between oxygen and nitrogen significantly impacts the photophysical properties in solution, the solid‐state, and 3D‐printed materials. This results in high quantum yield values and environmentally insensitive behavior. Using the full‐color range of these compounds, white light emission was attained utilizing stereolithography.

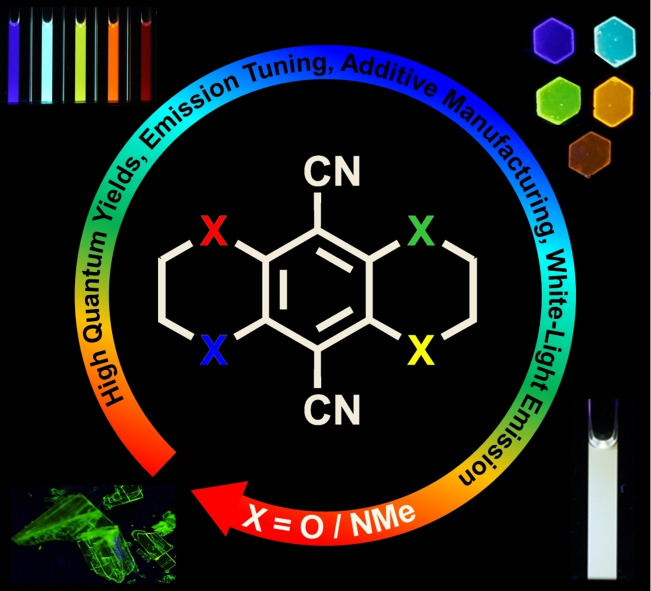

## Introduction

Discovering the unexpectedly strong fluorescent abilities of structurally simple luminophores resulted from comprehensive investigations of diverse molecular properties.[^1^, ^2^, ^3^] To understand this rare phenomenon further, several research groups have developed novel dyes implementing single‐benzene cores over the past five years. This approach allows designing specifically tailored fluorophores with biological tags to preserve their function without disrupting protein chemistry. Building on the pioneering work of Shimizu's group,^[4]^ Katagiri and colleagues published an illustrious report on the photophysical properties of a water‐soluble, green‐emitting (*λ*_em_=517 nm), tetra‐substituted single‐benzene fluorophore with a remarkable photoluminescence quantum yield (*Φ*_PL_) of 0.67 in water.^[5]^ Furthermore, SBBFs are particularly significant for applications in crystalline lasers and optical waveguides.[^6^, ^7^]

Achieving efficient fluorescence in organic materials using SBBFs is challenging due to the following criteria: 1) usually, an extended conjugated π system is required to facilitate electronic excitations using visible light; 2) the push‐pull character must be optimized, that enhances charge‐transfer (CT) excitations mediated by electron‐donating groups (EDG) and ‐withdrawing groups (EWG); 3) microenvironmentally insensitive emissive behavior, e. g., in dilute solution, amorphous powders, thin films, or specific materials, must be ensured by balancing planar, rigid cores and stacking‐preventing building blocks.^[8]^ This aspect highlights luminophores capable of surmounting detrimental quenching effects caused by aggregation or the unrestricted motion of flexible moieties in dilute solutions. Hence, compounds displaying SSSE properties combine the effects of chromophores that either experience aggregation‐caused quenching (ACQ) or exhibit aggregation‐induced emission (AIE).[^9^, ^10^] This versatility promotes a wide range of potential applications, including metal‐ion sensing,[^11^, ^12^] fingerprint visualization,^[13]^ or organic light‐emitting diodes (OLEDs).^[14]^

In previous studies, various substitution patterns for SBBFs have been explored,^[15]^ including di‐,^[16]^ tri‐,^[17]^ or tetra‐substituted benzene cores.^[18]^ The “X‐shaped” tetra‐substituted ring motif has become particularly popular due to its symmetrical dipolar structure. Frequently, diamino‐dicarbonyl^[19]^ or diamino‐diester^[20]^ groups are incorporated while being capable of excited‐state processes such as excited‐state intramolecular proton transfer (ESIPT)[^21^, ^22^] or intramolecular charge‐transfer (ICT).^[23]^

An intriguing report published by Zhang's group showcased the ability to cover the entire visible emissive spectrum from deep blue to red (*λ*_em_=432–654 nm) through utilizing different EDG and EWG in a subtle interplay.^[24]^ This fine‐tuning approach allows a wide range of mixed colors by adjusting the mixing ratio of the dyes. A significant challenge remains in achieving pure white light emission (WLE), with color coordinates of 0.33, 0.33 according to the colorimetric standard system CIE 1931 using simple organic materials.^[25]^ WLE is primarily utilized in electronic displays and light‐emitting diodes (LEDs) for everyday lighting.^[26]^ Consequently, there is a growing demand for developing organic WLE materials due to their simple tunability, low toxicity, and high versatility compared to inorganic, metal‐based alternatives.^[27]^

Hexa‐substituted SBBFs remain seldom reported.^[28]^ The terephthalonitrile (TN) core provides an excellent platform for synthesizing novel SBBFs by nucleophilic aromatic substitution reactions using various nucleophiles. This TN core was employed effectively in previous studies, including those by Maly and co‐workers,^[12]^ Banerjee and colleagues,[^29^, ^30^, ^31^] Feng *et al*.,^[32]^ and our research group.[^33^, ^34^, ^35^] In our exploration of reactions involving 2,3,5,6‐tetrafluoroterephthalonitrile (**TFTN**) with different nucleophiles, we successfully synthesized a series of compounds: **O_4_**, **N_1_O_3_**, **N_2_O_2_**, **N_3_O_1_**, and **N_4_**. Banerjee's group also reported **N_1_O_3_** and **N_2_O_2_** independently and investigated their photophysical properties in DMSO.[^30^, ^31^] Based on these findings, we aimed to conduct a comprehensive study of stepwise modulated compounds with varying N/O ratios, examining the photophysical properties in solution, solid‐state, thin films, 3D‐printed materials and micellar environments for potential applications in bioimaging and materials sciences.

Our design is based on the non‐classical idea of SSSE by merging static and dynamic functional groups in a hybrid fashion.^[8]^ We envisioned that the compounds meet the criteria for strong SSSE behavior, including sufficient conjugation, molecular stiffness through rigidified conformations, and stacking averting groups.^[36]^ Predicting the molecular arrangements and packing of these compounds, as well as their influence on solid‐state emissive properties, is difficult. Therefore, this study aims to contribute to the nascent topics of SBBFs and SSSE by attempting the ambitious challenge of synthesizing compounds that harness the advantages of both phenomena. Ultimately, this study intends to maximize the photophysical properties through precise synthetic modifications.

## Results and Discussion

### Design and Synthesis

**Figure 1:**
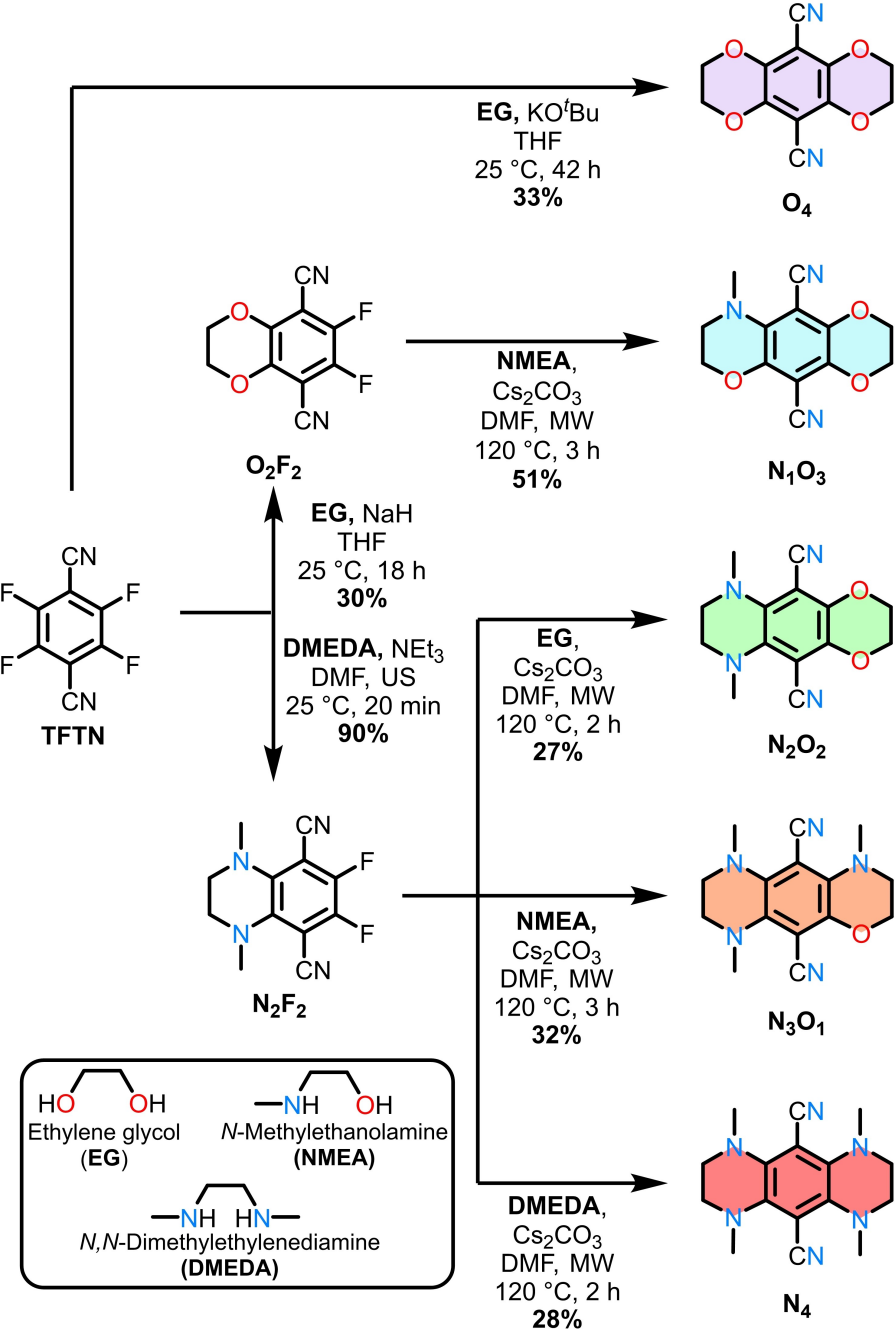
Overview of the performed synthetic routes yielding the desired compounds **O_4_**, **N_1_O_3_**, **N_2_O_2_**, **N_3_O_1_**, and **N_4_** with varying N/O ratio.

The library consisting of five target compounds was prepared using a straightforward two‐step pathway starting from **TFTN** (Figure 1). **TFTN** was selected as the starting material for purity validation because the absence of signals in the corresponding ^19^F‐NMR spectra confirms full conversions to hexa‐substituted luminophores. Synthetic procedures adapted from literature‐known nucleophilic aromatic substitutions were employed.^[29]^ Instead of primary amines, mono *N*‐methylated reagents were used to reduce potential side reactions of the formed products upon subsequent deprotonation, as reported by Hiscock *et al*.^[37]^ *N*‐methylated groups were used because they sufficiently suppress detrimental packing effects, according to the findings of Zhang's group.^[24]^ Since Banerjee's group reported the facile synthesis of **N_2_F_2_** with 85 % yield and excellent photophysical properties (*Φ*_PL_=0.76 in DMSO), we aimed for **N_2_F_2_** as an intermediary platform.^[29]^ Ultrasound‐assisted conditions improved the synthesis yield of **N_2_F_2_** to 90 % in just 20 minutes of reaction time.^[38]^ Unfortunately, compound **O_2_F_2_** could not be obtained using the same reaction conditions. Instead, the reaction was carried out by stirring ethylene glycol with sodium hydride and **TFTN** overnight, leading to 30 % yield of **O_2_F_2_** after column chromatography. Efforts to increase the reaction yield through dilution (from 0.2 m to 0.02 m) showed no improvement, but using potassium *tert*‐butoxide as the base yielded compound **O_4_** as the major product. For synthesizing the other target compounds, microwave‐assisted reaction conditions were employed to ensure full cyclization within a limited time frame, resulting in sufficient yields. Notably, our synthetic approach differs from Banerjee's by using anhydrous conditions (Cs_2_CO_3_ instead of KOH) in anhydrous solvents (DMF instead of water) to prevent the possible hydrolysis of the nitrile groups. The complete analytical characterization, including melting points, ^1^H‐, ^13^C‐, 2D‐NMR, IR spectroscopy, and high‐resolution mass‐spectrometry confirmed the proposed structures. Suitable crystals for X‐ray diffractometry were obtained for **O_4_**, **N_2_O_2_**, **N_3_O_1_**, and **N_4_**, ultimately validating the molecular build‐up. High‐performance liquid chromatography (HPLC) ensured high purity (>99 %) to exclude the effect of impurities on the photophysical properties (Figures S8‐S12).[^39^, ^40^]

### Optical Properties

Different molecular environments were selected to investigate the photophysical properties of the synthesized compounds. Studies in solution were performed using the solvents dichloromethane (DCM), tetrahydrofuran (THF), acetonitrile (ACN), methanol (MeOH), and DMSO to assess a range of polarities. The investigated compounds displayed limited solubility in more hydrophobic solvents than DCM, such as toluene or cyclohexane.

UV/Vis spectra were recorded in DMSO (see Figure S13). A gradual red shift of the bathochromic absorption maximum (*λ*_ab_) was observed with an increasing number of nitrogen atoms (Table 1). Using Lambert‐Beer's law, molar absorption coefficients were determined for the most bathochromic absorption bands measured in THF. The obtained values are consistent with the conventional range for SBBFs (6400‐8100 L**⋅**mol^−1^ **⋅**cm^−1^).^[15]^

**Table 1:** Selected photophysical properties in DMSO: absorption and emission wavelengths *λ*_ab_, *λ*_em_, molar absorption coefficients *ϵ* (*determined for the most bathochromic absorption bands in THF solution), Stokes shifts in nm (Δ*λ*) and cm^−1^ *(*Δν), absolute photoluminescence quantum yields *Φ*_PL_, amplitude‐weighted average fluorescence lifetimes *τ*_AvAmp_ [ns], and brightness determination (*B*=*Φ*_PL_ ⋅ *ϵ*).

	**O_4_**	**N_1_O_3_**	**N_2_O_2_**	**N_3_O_1_**	**N_4_**
*λ*_ab_ [nm]	389	410	437	450	467
*ϵ* [L**⋅**mol^−1^ **⋅**cm^−1^]*	8070	6840	6590	6490	7760
*λ*_em_ [nm]	433	495	545	603	652
Δ*λ*_em‐ab_ [nm]	44	85	108	153	185
Δν_ab‐em_ [cm^−1^]	2612	4188	4535	5638	6076
*Φ*_PL_	0.23±0.02	0.78±0.04	0.69±0.03	0.50±0.03	0.14±0.02
*τ*_AvAmp_ [ns]	3.237±0.003	13.98±0.03	13.38±0.09	12.67±0.02	4.057±0.009
*B* [L**⋅**mol^−1^ **⋅**cm^−1^]	1856	5335	4547	3245	1086

The excited‐state properties of the compounds were analyzed *via* steady‐state and time‐resolved photoluminescence spectroscopy. Noteworthy, all compounds displayed a minor solvatochromic behavior (see Figure S20). Consequently, DMSO was used as the only solvent for further investigations. Lifetime measurements revealed values up to 14 ns, confirming that fluorescence from an excited singlet state is the dominant emission mechanism (Table 1). Similar to the red shift of the absorption band, a steady red shift of the fluorescence band occurs from **O_4_** to **N_4_**. As a result, the Stokes shifts also progressively increase to nearly 200 nm (6076 cm^−1^) for **N_4_** (Table 1), which is highly desirable for biomedical applications to reduce self‐absorption^[41]^ and to enhance the signal‐to‐noise ratio.^[42]^ This leads to a most pronounced red emission (*λ*_em_=652 nm) for compound **N_4_** in dilute DMSO solution (*Φ*_PL_=0.14). Although red emissive colors of SBBFs were previously reported, these systems often consist of amino‐carbonyl skeletons that display ESIPT^[43]^ or ICT,^[44]^ leading to significant geometrical changes and larger Stokes shifts. **N_4_**, on the other hand, displays internal charge‐transfer as suggested by calculations assessing the charge‐transfer character of the excited state (*vide infra*). Thus, the enhanced donor strength is primarily responsible for the red emission.

**Figure 2:**
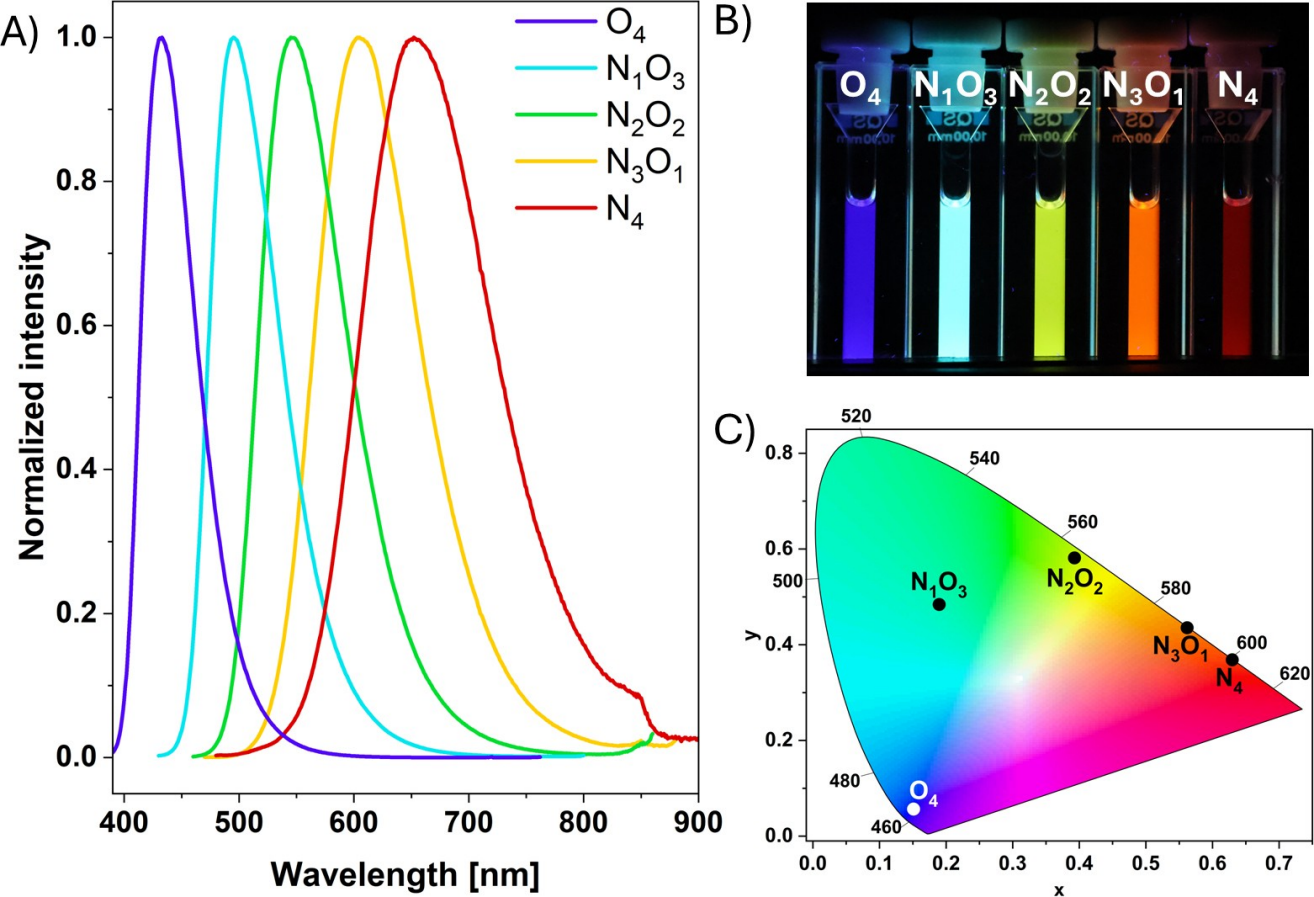
A) Normalized emission spectra of all compounds (10 μM) in DMSO solution (*λ*_ex_ (**O_4_**)=370 nm; *λ*_ex_ (**N_1_O_3_**)=410 nm; *λ*_ex_ (**N_2_O_2_**)=440 nm; *λ*_ex_ (**N_3_O_1_**)=450 nm; *λ*_ex_ (**N_4_**)=470 nm); B) respective pictures taken under 365 nm UV light; C) corresponding CIE 1931 chromaticity plot.

Chromaticity diagrams were generated using the CIE 1931 color spaces (Figure 2).^[45]^ Here, the entire color spectrum can be covered by the fluorescence of the compounds, where **N_3_O_1_** and **N_4_** even show fully saturated orange and red colors, respectively. Remarkably high photoluminescence quantum yields were measured for **N_1_O_3_** (0.78) and **N_2_O_2_** (0.69). These values deviate from the reported literature values (*Φ*_PL_=0.84 and 0.49, respectively). This difference can be attributed to using deoxygenated solvents in this study and applying the absolute quantum yield measurement method. With the determined absorption coefficients, brightness *B* values were calculated according to Klymchenko *et al*.^[46]^ **N_1_O_3_**, **N_2_O_2_**, and **N_3_O_1_** exhibit higher brightness values than the literature‐known fluorescence standard quinine sulfate (3112 L**⋅**mol^−1^ **⋅**cm^−1^), highlighting the radiant power of the presented compounds.[^47^, ^48^]

**Figure 3:**
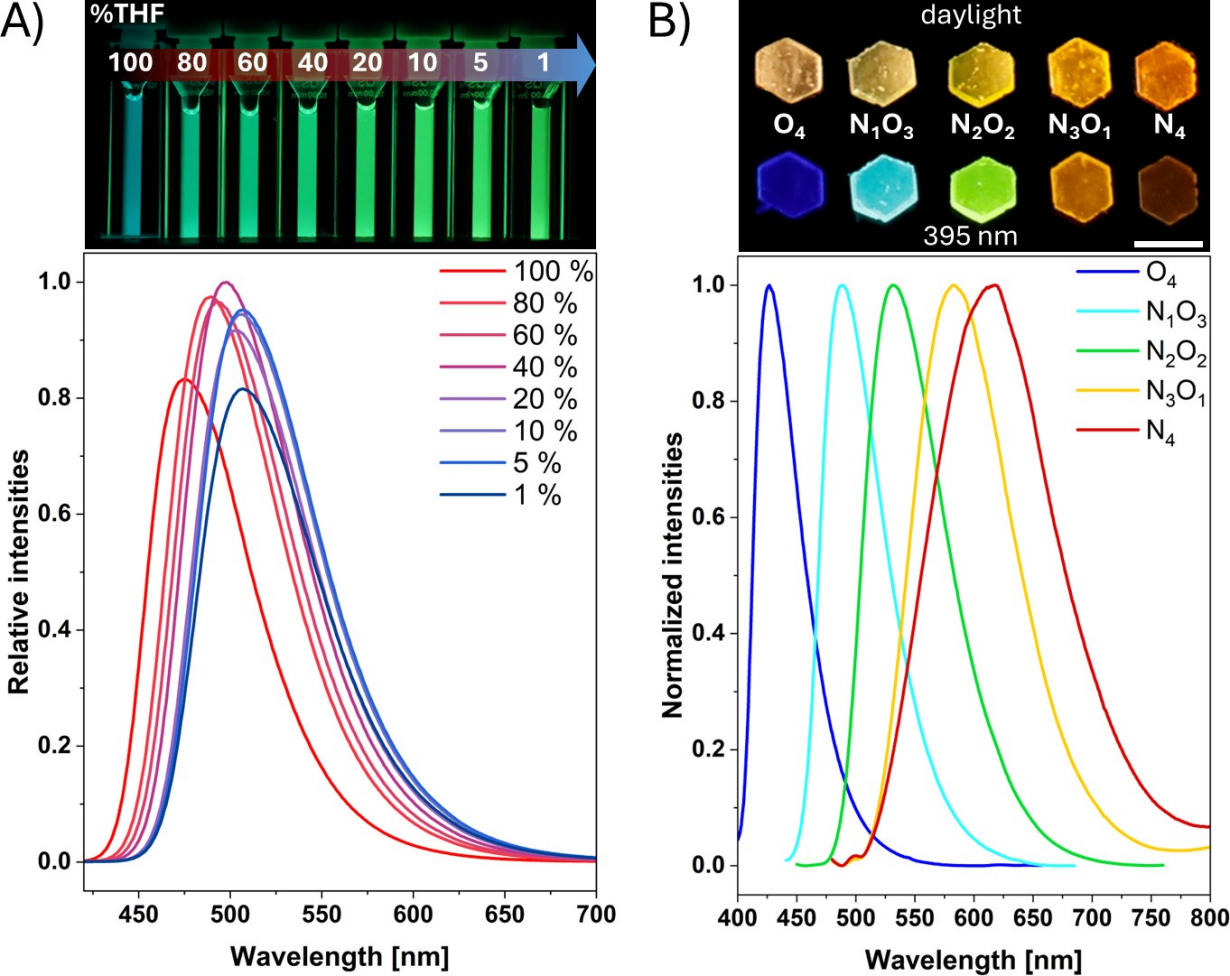
A) Relative emission intensities for an aggregation series of **N_1_O_3_** (each 10 μM, *λ*_ex_=400 nm) in THF/H_2_O with the corresponding pictures taken under 365 nm UV light (percentage corresponds to THF content); B) normalized emission spectra of all compounds (*λ*_ex_ (**O_4_**)=380 nm; *λ*_ex_ (**N_1_O_3_**)=400 nm; *λ*_ex_ (**N_2_O_2_**)=430 nm; *λ*_ex_ (**N_3_O_1_**)=460 nm; *λ*_ex_ (**N_4_**)=460 nm) as 3D‐printed hexagons using SLA (0.1 wt% each). Respective pictures taken under daylight (top) and under 395 nm UV light (bottom). Scale bar: 4 mm.

Except for weak yellow emission of **N_4_**_,_ the observed color series in solution is analogous to those of the amorphous powders (see Figure S23). The quantum yields of all five powders decrease significantly, with only **O_4_** and **N_1_O_3_** showing moderate values above 0.10 (see Table S3). Aggregation‐induced effects were examined based on the low solubility of the compounds in THF/water mixtures to investigate the impact of rigidified molecular surroundings. All compounds, except for **N_1_O_3_**, exhibit a significant relative decrease in their respective fluorescence intensity, indicating their behavior as aggregation‐caused emission weakeners (ACEW) or aggregation‐caused quenchers (ACQ) (see Figure S32). On the other hand, the nearly constant emission of **N_1_O_3_** allows a classification as a solution and solid‐state emitter. Hence, to the best of our knowledge, **N_1_O_3_** presents the first single‐benzene‐based fluorophore able to maintain balanced emission irrespective of the molecular surrounding (Figure 3A).

Distinguished behavior can be observed upon embedding the compounds in different polymeric materials. Thin films of poly(methyl methacrylate) (PMMA) with 1 wt% of compound were prepared using the spin‐coating method. Within these matrices, non‐radiative deactivation pathways are suppressed, reducing *k*_nr_ values and promoting fluorescent decay. Absorption wavelengths for these PMMA films are virtually identical to the DMSO solutions, whereas emission wavelengths are less red shifted, leading to smaller Stokes shifts (Figure S25). Except for **O_4_**, all quantum yield values of the PMMA films are above 0.49, indicating the high applicability of these dye‐incorporated matrices. **N_2_O_2_** (*Φ*_PL_=0.76) even surpasses **N_3_O_1_** (*Φ*_PL_=0.65) and **N_1_O_3_** (*Φ*_PL_=0.61, see Table S4).

To further explore potential applications, the compounds were evaluated for their suitability as fluorescent additives in 3D‐printed materials using stereolithography (SLA, Figure 3B). The compounds were dissolved in DCM and incorporated into an acrylate‐based resin (0.1 wt%). After evaporation of the solvent, the resin was photopolymerized (405 nm) to obtain elastomeric hexagons (4 mm x 4 mm). The elastomeric properties can be ascribed to the hydrogen‐bonding abilities of the used monomer 2‐[[(butylamino)carbonyl]oxy]ethyl acrylate (BCOE).^[49]^ This high‐polar surrounding leads to a solvatochromic effect compared to PMMA‐based materials, as indicated by the red‐shift in absorption and emission maxima for all compounds (Table S5). All 3D‐printed objects show complete homogeneity, as confirmed by stereo microscopy (Figure S29). As expected, the quantum yields are similar to those of the PMMA films, with the highest values exhibited by **N_1_O_3_** (0.67) and **N_2_O_2_** (0.70), further highlighting the significant potential of SBBFs in materials application.

Moreover, the ability of the compounds to detect pH changes in DCM solutions was evaluated upon adding 100 eq. trifluoroacetic acid (TFA) and afterward 500 eq. triethylamine (TEA, see ESI, Fig. S34). Interestingly, a small increase in relative fluorescence intensity occurred for **O_4_**, whereas **N_1_O_3_** and **N_2_O_2_** did not show a significant effect. For **N_3_O_1_**, the emission decreases in the presence of TFA and is partially recovered upon subsequent neutralization. For **N_4_**, however, a hypsochromically shifted emission band appeared. After prolonged storage, irreversible degradation of the compound was detected (see ESI, Fig. S33).

### White Light Emission (WLE) Experiments

After investigating the photophysical properties of the SBBFs in various molecular environments, the potential to obtain WLE by fabricating materials composed of these compounds was explored. The *Commission Internationale d′Eclairage* (CIE) defines WLE through the CIE 1931 color space diagram with chromaticity coordinates of x=0.33, and y=0.33. The general approach to constructing systems that exhibit WLE involves combining red, green, and blue (RGB) components or utilizing complementary colors such as yellow and blue.^[45]^ However, intermolecular interactions and complex processes such as energy transfers must be considered in these approaches.

Recently, Sarih *et al*. described a method for generating WLE by mixing three fluorophores while considering their relative emission intensities.^[50]^ By adopting this approach, it was anticipated that WLE could be achieved by combining the compounds **O_4_**, **N_2_O_2_**, and **N_3_O_1_**. Adding green (**N_2_O_2_**) and orange (**N_3_O_1_**) emission bands yields yellow emission. Hence, adding the blue emission from **O_4_** was expected to lead to WLE.

**Figure 4:**
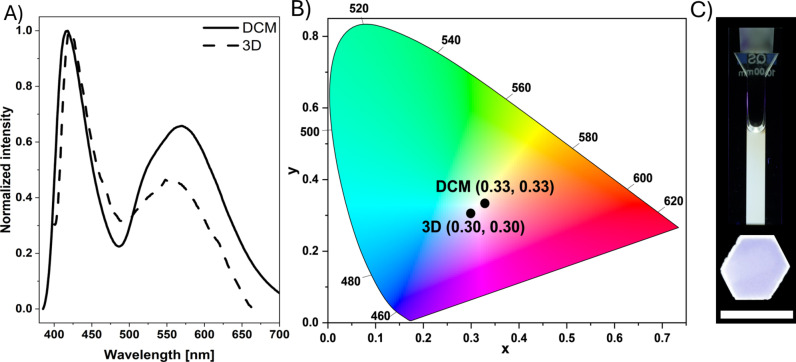
A) Normalized emission spectra (*λ*_ex_=365 nm) of the white light‐emitting mixture (1.141 eq. **O_4_**, 0.385 eq. **N_2_O_2_**, and 1 eq. **N_3_O_1_**) in DCM (solid line) and 3D‐printed hexagon (dashed line); B) corresponding CIE 1931 chromaticity plot; C) respective pictures taken under 365 nm UV light. Scale bar: 4 mm.

Thus, the emission intensities of these three compounds in DCM solution were correlated after being excited at 365 nm. The relative emission intensity of **N_3_O_1_** compared to **N_2_O_2_** was measured to be 0.385. Accordingly, the yellow component was generated by mixing one equivalent of **N_3_O_1_** with 0.385 equivalents of **N_2_O_2_**. Given the relative emission intensity of **N_3_O_1_** compared to **O_4_** at 0.571, twice the equivalents of **O_4_** (1.142 eq.) were combined with the yellow component. As a result, perfect chromaticity coordinates of x=0.33 and y=0.33 were attained (Figure 4A; see Figure S35 for further details).

After extended irradiation, the observed WLE quickly decomposed to a blue color due to rapid photodegradation, most likely due to ^1^O_2_‐mediated photooxidation (Figure S36). To enhance the photostability of our WLE system and suppress oxidation processes, we envisioned incorporating the WLE mixture into a 3D‐printed material. Although integrating WLE into additively manufactured materials is promising, the number of studies in this area is scarce,[^51^, ^52^] and the reported examples are mostly metal‐based.[^53^, ^54^]

Initially, the same ratios used in the DCM solution were opted for a 3D‐printed object, with 0.1 wt% of the compounds incorporated into the resin. However, the yellow component was dominant, resulting in a yellow‐emissive hexagon. To address this issue, the amount of **O_4_** was doubled to 2.284 eq., while the ratios of **N_2_O_2_** and **N_3_O_1_** were maintained. The resultant mixture was utilized for 3D‐printing the hexagon, which exhibited WLE characterized by x and y coordinates of 0.30 and 0.30, closely approximating white light (Figure 4B). This marks the first instance of a white light‐emitting 3D‐printed object derived from SBBFs. Notably, immobilizing the mixture within the polymeric material significantly reduced the extent of photodegradation. After ten minutes of irradiation with a 365 nm LED, the x and y coordinates shifted to 0.25 and 0.26, respectively. This reduced degradation can be attributed to the enhanced shielding from triplet dioxygen and its slower diffusion into the material (Figure S38).

### X‐ray Diffractometric Analysis

**Figure 5:**
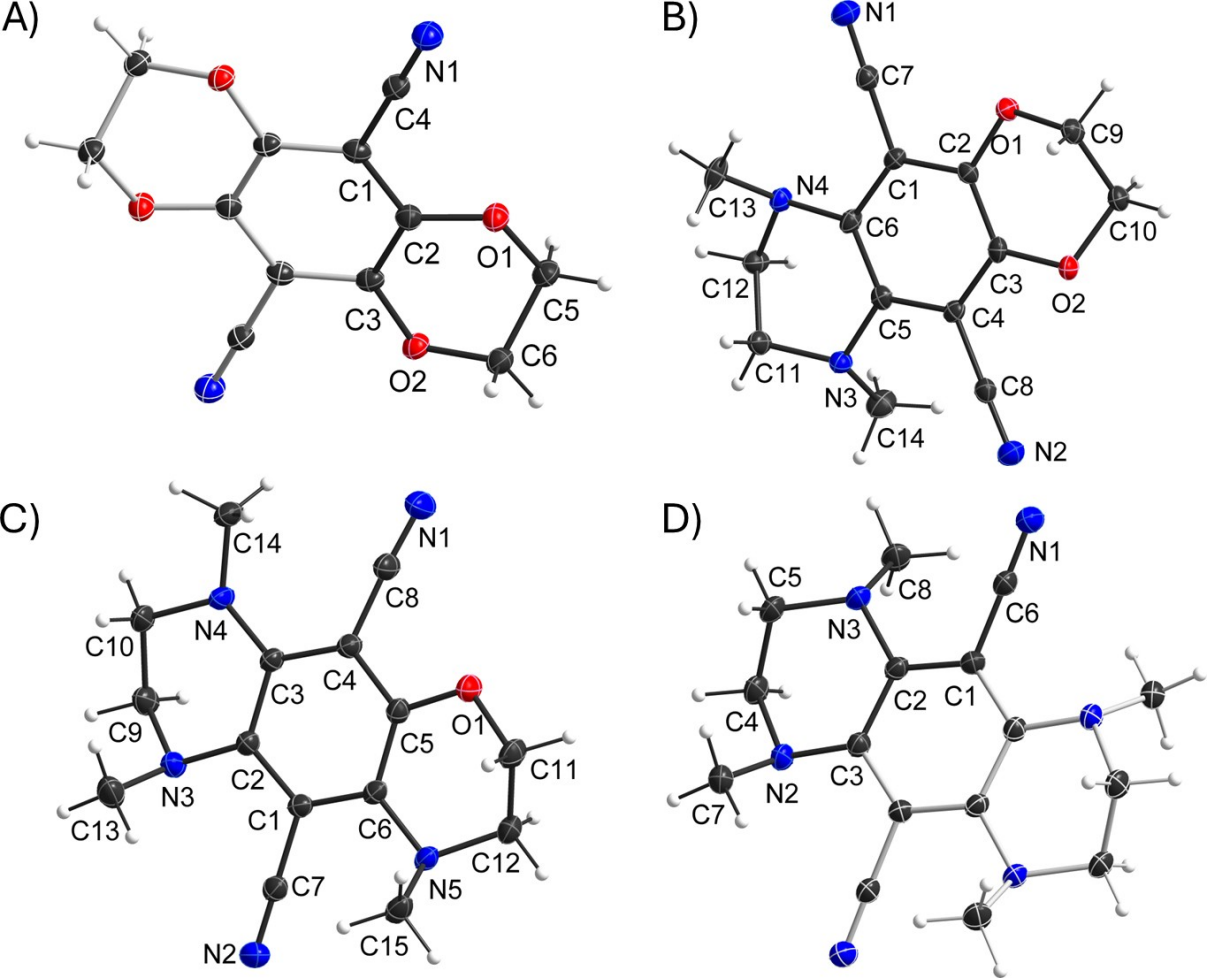
Molecular structures in the crystals of the compounds A) **O_4_**; B) **N_2_O_2_**; C) **N_3_O_1_**; D) **N_4_**. Displacement ellipsoids displayed at 50 % probability levels. Parts generated by symmetry are displayed in pale colors.

To verify the molecular structure, crystals of four compounds were grown and measured (Figure 5) using X‐ray diffraction (Deposition Numbers CCDC 2393405–2393408 contain the crystallographic data in this study).^[55]^ Despite numerous tries, no suitable crystals of **N_1_O_3_** could be obtained due to poor scattering.

**O_4_** and **N_4_** crystallize with the molecule on a special position (center of inversion) in the monoclinic space group *P*2_1_/*n* and the triclinic space group *P* $\bar{1}$ , respectively. Consequently, the central phenyl ring is perfectly planar. Compound **N_2_O_2_**, which crystallizes in the orthorhombic space group *Pnma*, is disordered over a mirror plane. Thus, quantitative results should be carefully assessed. **N_3_O_1_** crystallizes in the monoclinic space group *P*2_1_/*c*. As can be seen from the deviations from the best plane of the central phenyl ring (Table 2), the core moiety of the molecules including the heteroatomic substituents is planar. In all compounds, the ethylene bridges adopt staggered conformations. Except for one group in **N_3_O**_1_, all N−Me carbon atoms are located significantly off the best plane, suggesting sp^3^ hybridization of the nitrogen atoms and only partial contribution of their lone pairs to the conjugated systems.

**Table 2:** Deviations from the best plane of the central phenyl ring [Å]. [a] Due to symmetry defined by only three atoms. [b] Low reliability of the data due to disorder.

	substituent O/N	methyl@N	nitrile N	r.m.s. from best plane
**O_4_**	−0.026(3) 0.0042(16)	—	0.010(3)	0^[a]^
**N_2_O_2_** ^[b]^	−0.061(5) (O) −0.021(13) (O) −0.001(12) (N) −0.09(3) (N)	1.10(3) −1.152(11)	−0.023(16) 0.196(9)	0.0113
**N_3_O_1_**	0.040(2) (O) 0.012(3) (N) 0.025(3) (N) −0.073(2) (N)	1.309(3) 0.462(3) 1.121(3)	−0.439(3) −0.197(3)	0.0246
**N_4_**	0.0180(16) 0.116(3)	1.234(2) −1.005(5)	0.548(3)	0^[a]^

This steric strain caused by the N−Me groups pushes the nitrile groups out of‐plane (deviation of up to 0.548(3) Å in **N_4_** compared to 0.101(3) Å in **O_4_**). In addition, the steric strain of *N*‐methyl amino groups leads to an in‐plane push of the nitrile groups towards the oxygen‐bridged side, as evident by the difference in the bonding angles at the *ipso* carbon atom of the central ring (**N_3_O_1_**: 8.8(3)°, **N_2_O_2_**: approx. 4°).

**Figure 6:**
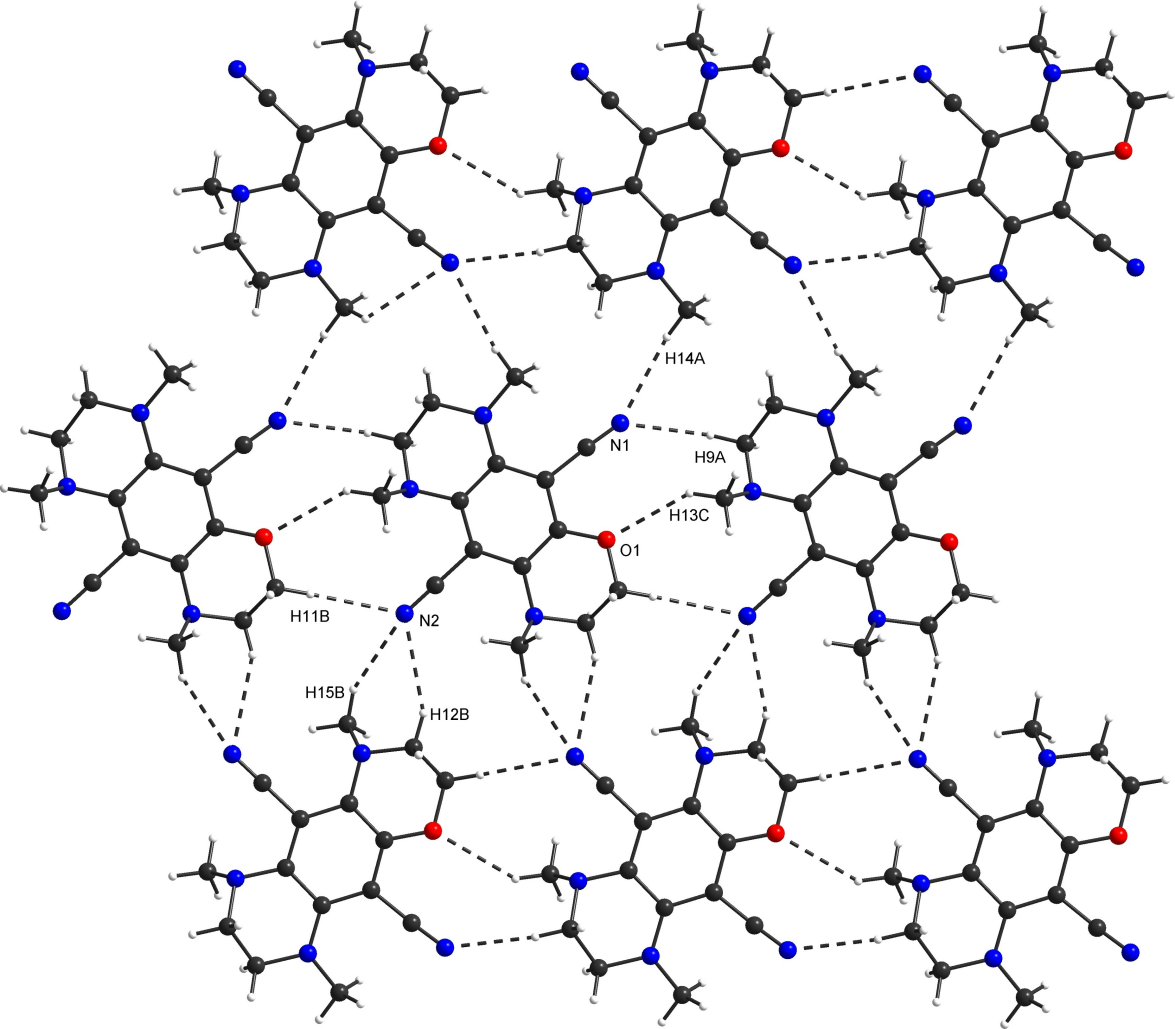
Layer parallel to $\left(\bar{1}\bar{0}\bar{1}\right)$ in the packing of **N_3_O_1_**.

The intermolecular contacts in the crystal structures were analyzed to identify the most relevant interactions in the crystal packing. **N_3_O_1_** is governed by non‐classical hydrogen bonds that lead to a layer formation parallel to $\left(\bar{1}\bar{0}\bar{1}\right)$ . Non‐classical hydrogen bonds and CH⋅⋅⋅π interactions connect these layers (Figure 6). It is important to note that low displacement from the central ring of one of the N−Me groups allows a significantly closer approach of the layers (3.83 Å) and thus increases the packing density. Moreover, the sp^2^ hybridization of the corresponding nitrogen atom leads to a larger conjugation, which results in improved acceptor properties for CH⋅⋅⋅π interactions. These effects possibly compensate the distortion of the molecular geometry. The packing of **N_4_** is similar; however, the inter‐layer distance is larger due to all N−Me groups oriented of the central plane (4.39 Å). Since the N−Me groups are all sp^3^‐hybridized, only the central ring‘s π system – including the nitrile groups – remains as an acceptor for CH⋅⋅⋅π interactions. In **N_4_**, the layers are arranged parallel to (110). The fundamental packing motif of **O_4_** is determined by chains formed by non‐classical hydrogen bonds. These are stacked, resulting in layers connected by π⋅⋅⋅π of the nitrile groups and the central phenyl ring. The layers are bonded by non‐classical hydrogen bonds (see Figures S59‐S60). The stacking directions within neighboring layers are not in parallel.

Furthermore, the interactions of the four compounds in the crystal packings were quantitatively evaluated using Hirshfeld surface (HS) analysis from the *CrystalExplorer17* program.^[56]^ The number of O⋅⋅⋅H interactions in the crystal decreases with the number of oxygens contained in the structure (**O_4_**: 26 %; **N_3_O_1_**: 5 %, see Table S15). This trend does not apply to the number of nitrogen atoms, as, *i. e*., insignificant differences of N⋅⋅⋅H interactions are present for **O_4_** (33 %) and **N_4_** (35 %). This can be explained since the nitrogen atoms of the nitrile groups are the major donor part for these interactions (N⋅⋅⋅H−C). As a result, the crystal packing of **O_4_** is dominated by O⋅⋅⋅H and N⋅⋅⋅H contacts, leading to fewer H⋅⋅⋅H interactions (**O_4_**: 13 %; **N_3_O_1_**: 46 %). This becomes evident in the HS of **O_4_** mapped with the normalized contact distance (*d*_norm_, see Figure S62). **O_4_** displays a slipped π‐π stacking. Without any oxygen atoms, the crystal packing of **N_4_** is almost completely based on N⋅⋅⋅H and H⋅⋅⋅H contacts. It must be noted that C⋅⋅⋅H interactions increase for this compound while C⋅⋅⋅C contacts do not occur, indicating that the high steric hindrance induced by the N−Me groups impedes π‐π stacking of the benzene cores. This is evident by the HS of **N_4_** mapped with curvedness (see Figure S64). In conclusion, these four crystal structures are dominantly determined by non‐classical hydrogen bonding interactions (O⋅⋅⋅H−C, N⋅⋅⋅H−C), quantitatively controlled by the number of oxygen atoms present in the core.

### Quantum Chemical Calculations

Quantum chemical calculations using *Gaussian 16* ^[57]^ were performed to gain further insight into the photophysical properties. The geometry optimization of the electronic ground states S_0_ was performed using the density functional theory (DFT); the geometrical parameters of the excited singlet states S_1_ were calculated using the time‐dependent density functional theory (TD‐DFT).^[58]^ The functional PBE0^[59]^ with the TZVP^[60]^ basis set was employed. Grimme's dispersion correction (GD3BJ) was applied for the geometry optimizations in the S_0_ states.^[61]^ To consider solvent effects, the calculations were conducted using the polarizable continuum model (PCM), which included the presence of DMSO.

Since the N−Me groups can adopt axial or equatorial orientations, the energies of the optimized conformers (all‐axial or all‐equatorial) were investigated. As expected from the obtained crystal structures, the all‐axial position is energetically more favored in the electronic ground state compared to the all‐equatorial position with an increasing number of N−Me groups, most prominent for **N_4_** (*Δ*E=8.2 kcal**⋅**mol^−1^). However, it must be assumed that these conformers can convert into each other.

**Figure 7:**
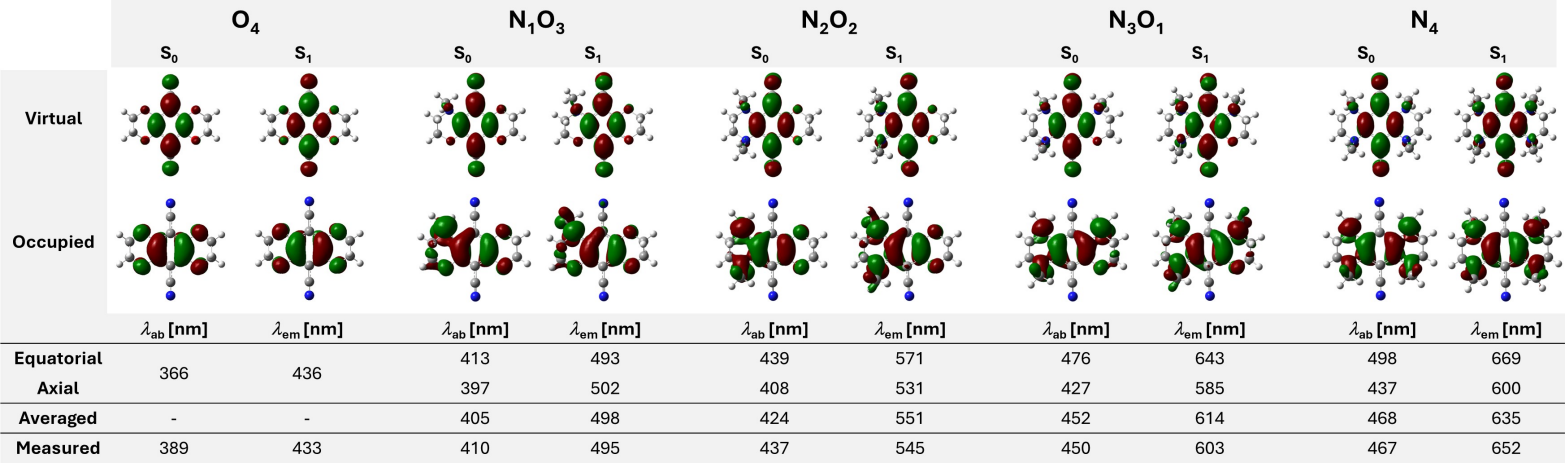
Top: calculated optimized geometries in the S_0_ and S_1_ states for the all‐axial oriented conformers, along with the respective NTO pairs. Bottom: calculated absorption and emission wavelengths for all‐axial and all‐equatorial conformers, averaged values and measured wavelengths in DMSO. Calculations were performed using PBE0(DMSO)‐GD3BJ/TZVP for the S_0_ states and TD‐PBE0(DMSO)/TZVP for the S_1_ states.

The character of the lowest excited singlet states is mainly dominated by a monoelectronic excitation involving the highest occupied molecular orbitals (HOMOs) to the lowest unoccupied molecular orbitals (LUMOs). Natural transition orbital (NTO) pairs, which qualitatively describe electronic excitation processes, were calculated for the all‐axial conformers. The LUMOs are mainly located in the vertical axis elongated over the electron‐withdrawing nitrile groups (Figure 7). On the other hand, the HOMOs are mainly distributed over the horizontal axis due to the electron‐donating oxy/amino groups. Calculations of electron density differences suggest intramolecular charge transfer (ICT) processes. These are indicated by a reduction in the electron density at the donating bridging atoms upon absorption and transferred to the nitrile groups (see Figures S65‐S79). The absence of a strong solvatochromic effect can be ascribed to the small dipole moments *μ* (<5 D), which do not change significantly in the excited states.

The measured absorption wavelengths are approximately comparable with the average values of the calculated absorption wavelengths for the all‐axial and all‐equatorial oriented conformers (Figure 7). The same trend can be observed for the theoretical emission wavelengths. The calculated averaged values nearly match the measured emission wavelengths. Hence, the experimental trend can be well correlated with the calculated absorption and emission wavelengths. These results demonstrate the facile applicability of calculating the electronic properties of SBBFs.

### Cell Assays and Fluorescence Microscopy

**Figure 8:**
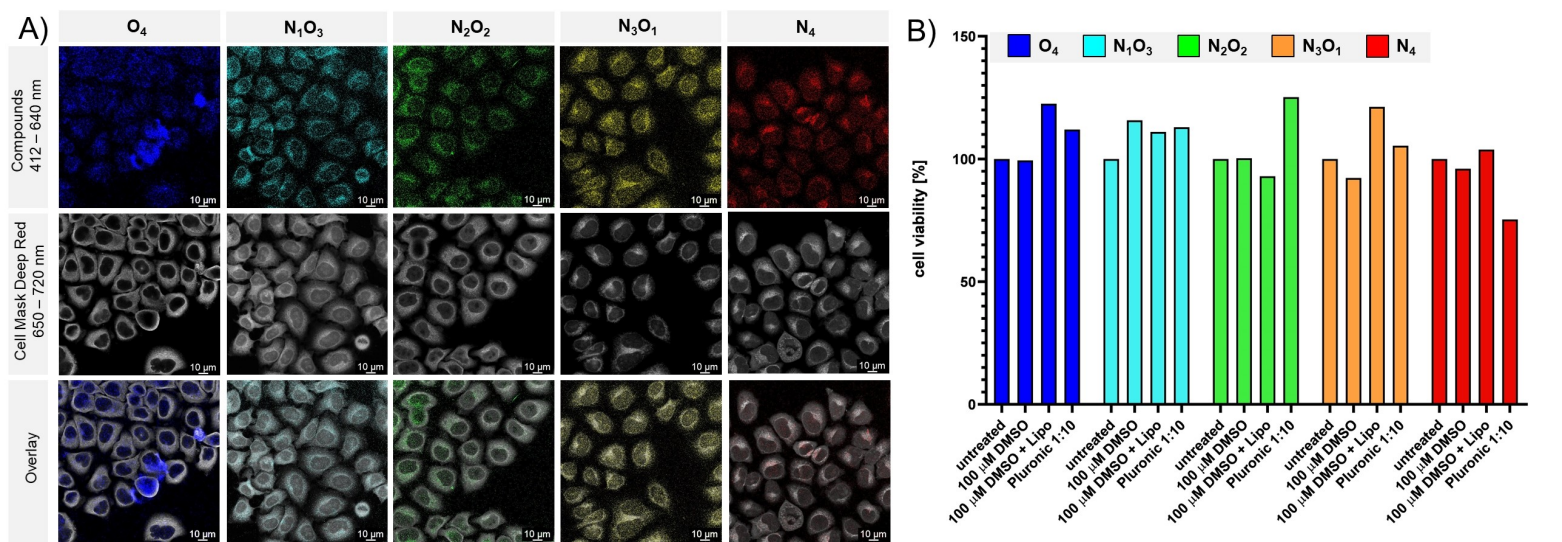
A) Internalization of Pluronic^®^ F‐127 micelle‐enclosed compounds (1 : 10 dilution in a total volume of 200 μL) in HeLa Kyoto cells imaged by confocal laser scanning microscopy after 24 hours of treatment. Cells were imaged after fixation and staining with HCS CellMask™ Deep Red (shown in grey). Compounds in Pluronic^®^ F‐127 micelles are shown in the color of their actual emission wavelength. Contrast was adjusted by 20–40 % to optimize the visibility of the compounds. Scale bar: 10 μm. B) Toxicity studies of compounds in HeLa Kyoto cells after 24 hours of treatment (100 μM in 100 % DMSO, 100 μM in 100 % DMSO preincubated with 1 μL of Lipofectamine™ 2000 and in a ratio of 1 : 10 solved in Pluronic^®^ F‐127. The CellTiter® Aqueous One Solution Kit was used to determine cell viability, and absorbance at 490 nm was measured according to the instructions of the manufacturer (Promega). Cell viability is determined in % after blank subtraction followed by normalization to the negative control of each series.

Ultimately, the potential of the novel SBBFs was assessed by performing cell viability assays and confocal laser scanning microscopy (CLSM) of HeLa Kyoto cells. Briefly, cells were fixed and stained for analysis of cellular uptake using *CellMask* ^*®*^ *Deep Red* (em: 450–600 nm) to enable unambiguous identification of single cells due to the full‐color emission range of the investigated compounds. The samples were excited with a 405 nm laser, and the emission was detected between 412–640 nm depending on the respective emission maxima of the individual compounds, as indicated (Figure 8). Since the compounds are insoluble in water, concentrated DMSO stock solutions (40 mM) were prepared and incubated with the cells for 24 h. Unfortunately, only negligible cellular internalization of the aggregated compounds was observed. Efforts to promote cellular uptake by enhancing the DMSO content or adding 1 μL of Lipofectamine^TM^ 2000, the readily available gold standard for lipofection experiments, failed (see Figures S80‐S81). Therefore, the compounds were encapsulated in Pluronic^®^ F‐127 nanoparticles, which are regularly utilized due to their biocompatibility and surfactant properties.[^22^, ^62^] By applying a protocol from Gallavardin *et al*.^[63]^ doped nanoparticles were generated in water, confirmed by the strongly luminescent nature (see Figure S30), whereas the aforementioned aggregates were less emissive (Figure S32). Surprisingly, **N_4_** showed nearly negligible yellow fluorescence, although displaying a distinctive orange color under daylight (see Figure S31). This is possible either due to the high concentration and near‐proximity of compounds inside the micelles leading to an aggregation‐caused quenching effect or due to the presence of water inside the micelle, which is often responsible for the loss of emission.^[64]^ Besides showing the Tyndall effect (see Figure S31), the size of the nanoparticles was verified by dynamic light scattering (DLS) measurements, revealing hydrodynamic radii of 4–25 nm.

The Pluronic^®^ F‐127 nanoparticles were then incubated with the cells for 24 h. Here, internalization in vesicular morphologies inside the cytoplasm can be observed (Figure 8A). These results demonstrate that Pluronic^®^ F‐127 can be used as an uptake facilitator. In addition, HeLa Kyoto cells were treated with the respective compounds in the indicated concentrations to investigate their effect on cell viability over 24 h (Figure 8B). Compounds in DMSO were again either directly added to the culture medium, preincubated with Lipofectamine™ 2000, or enclosed in Pluronic^®^ F‐127. All solvents were likewise used as controls.

Remarkably, all compounds revealed only marginal toxicity, even at higher concentrations. Decreased aggregation in Pluronic nanoparticles resulted in a higher bioavailability, as evident from the microscopic analyses where the compounds were evenly distributed in the cytoplasm of the cells. Although this resulted in a slightly increased cytotoxicity, and we are well aware that the bio‐applicability of our compounds is still amendable, these novel emitters might prove to be valuable tools for future biomedical applications.

## Conclusions

In conclusion, this study reports on the comprehensive investigation of the distinctive attributes comprising five novel single‐benzene‐based fluorophores. These maximum two‐step‐synthesis accessible compounds differ in the amount of alkyloxy and amino bridging groups, which affect the photophysical properties. Consequently, the SBBFs cover a broad range of full‐color emission. Several molecular surroundings were analyzed spectroscopically, revealing unusually high Stokes shifts in DMSO and impressive quantum yield values in PMMA films. Measurements of amorphous powders and aggregates demonstrated the classification of **N_1_O_3_** as a rare solution‐ and solid‐state emitter with a single‐benzene‐based core. In addition, these SBBFs were successfully incorporated into 3D‐printed objects for the first time, demonstrating their potential as materials for technological applications. By carefully controlling the ratio of the compounds, white light emission was achieved in solution and in an additively manufactured material. This opens the possibility of designing and creating white light emitting devices using stereolithography in the future. X‐ray diffractometric analysis and quantum chemical calculations enabled discussions of geometrical conformations and packing rearrangements, explaining the stacking behavior and allowing interpretation of the experimental data. Finally, using Pluronic^®^ F‐127 as a facilitator of cellular internalization was investigated, enabling possibilities for bioimaging of these SBBFs, particularly if water‐solubilizing groups or moieties for bioconjugation are inserted. This study highlights the importance of comprehensive investigations to inspire novel findings regarding the modular interplay of functional groups in minimalized fluorophores. These results contribute to the growing collection of SBBFs and dyes exhibiting SSSE and establish a connection between these nascent research areas.

## Supporting Information

The Supporting Information contains detailed synthetic procedures, analytical data, comprehensive characterization of photophysical properties, X‐ray diffractometric analysis, crystal packing analysis, quantum chemical calculations and cell experiments. Additional references have been cited within the Supporting Information.[^65^, ^66^, ^67^, ^68^]

## Author Contributions

A. Huber: conceptualization, formal analysis, methodology, visualization, investigation, writing – original draft, writing – review & editing; L. Schmidt: formal analysis, investigation, visualization, writing – original draft; T. Gatz: investigation, visualization; J. Bublitz: investigation, visualization; T. Rex: investigation, formal analysis; S. T. N. Sailaja: methodology, writing ‐ review & editing; E. Verheggen: investigation; L. Höfmann: formal analysis; C. Wölper: Data curation, writing – original draft; C. A. Strassert: resources, writing ‐ review & editing; S. K. Knauer: resources, writing ‐ review & editing; J. Voskuhl: conceptualization, resources, visualization, supervision, writing – review & editing.

## Conflict of Interests

The authors declare no conflict of interest.

1

## Supporting information

As a service to our authors and readers, this journal provides supporting information supplied by the authors. Such materials are peer reviewed and may be re‐organized for online delivery, but are not copy‐edited or typeset. Technical support issues arising from supporting information (other than missing files) should be addressed to the authors.

Supporting Information

## Data Availability

The data that support the findings of this study are available from the corresponding author upon reasonable request.
