# Supplementary material for: Stepwise Modulation of Bridged Single‐Benzene‐Based Fluorophores for Materials Science
Source: Chemistry. 2025 Jan 22;31(14):e202404263. doi: 10.1002/chem.202404263 (PMC11886771; doi:10.1002/chem.202404263)
Supplement: Supplementary file 1 — Supporting Information [file CHEM-31-e202404263-s001.pdf]

Chemistry–A European Journal

Supporting Information

Stepwise Modulation of Bridged Single-Benzene-Based Fluorophores for Materials Science

Alexander Huber, Laura Schmidt, Tim Gatz, Jana Bublitz, Tobias Rex, Sidharth Thulaseedharen Nair Sailaja, Elisabeth Verheggen, Lea Höfmann, Christoph Wölper, Cristian A. Strassert, Shirley K. Knauer, and Jens Voskuhl*

Supporting Information

Stepwise Modulation of Bridged Single-Benzene-Based Fluorophores for Materials Science

Alexander Huber[a], Laura Schmidt[b], Tim Gatz[a], Jana Bublitz[a], Tobias Rex[c], Sidharth Thulaseedharen Nair Sailaja[a], Elisabeth Verheggen[a], Lea Höfmann[a], Christoph Wölper[d], Cristian A. Strassert[c], Shirley K. Knauer[b], Jens Voskuhl[a],*

[a] M. Sc. A. Huber, B. Sc. T. Gatz, B. Sc. J. Bublitz, Dr. S. T. N. Sailaja, E. Verheggen, M. Sc. L. Höfmann, Prof. Dr. J. Voskuhl, Faculty of Chemistry (Organic Chemistry), CENIDE and Center of Medical Biotechnology (ZMB), University of Duisburg-Essen, Universitätsstraße 7, 45141 Essen, Germany, *corresponding author, E-Mail: jens.voskuhl@uni-due.de.

[b] M. Sc. L. Schmidt, Prof. Dr. Shirley K. Knauer, Department of Molecular Biology II, Center of Medical Biotechnology (ZMB), University of Duisburg-Essen, Universitätsstraße 2, 45141 Essen, Germany.

[c] M. Sc. T. Rex, Prof. Dr. C. A. Strassert, Institut für Anorganische und Analytische Chemie, CeNTech, CiMIC, SoN, Universität Münster, Heisenbergstraße 11, 48149 Münster (Germany)

[d] Dr. C. Wölper, Faculty of Chemistry (Inorganic Chemistry), University of Duisburg-Essen, Universitätsstraße 7, 45141 Essen, Germany.

CONTENT

1 General information and concept 3

2 Synthetic procedures 6

General procedures 6

General procedure (GP1): Microwave-assisted nucleophilic aromatic substitution 6

Overview of performed syntheses 6

Synthetic procedures 7

NMR spectra 11

HPLC chromatograms 17

3 Photophysical properties 19

UV/Vis absorption spectra 19

Photoluminescence in DMSO 21

Solvatochromism 23

Photoluminescence in powders 25

Photoluminescence in PMMA films 27

Photoluminescence in 3D-printed hexagons 29

Photoluminescence in Pluronic® F-127 nanoparticles 31

Aggregation series 32

Acidochromism 34

White Light Emission experiments 36

In DCM 36

In 3D-printed hexagon 37

Summary of the photophysical properties 38

Lifetime reports 39

4 X-ray diffractometric analysis on single crystals 49

5 CrystalExplorer 52

6 Quantum chemical calculations 56

Cartesian coordinates of the optimized geometries 56

Natural Transition Orbitals and Electron density differences 63

7 Cell assays and microscopy 68

8 Literature 70

1 GENERAL INFORMATION AND CONCEPT

CHEMICALS AND SYNTHESIS

Commercially available chemicals were purchased from Deutero, TCI, Sigma Aldrich, Acros Organics, and Fisher Scientific and used without further purification. Caesium carbonate was dried under vacuum. Anhydrous THF was dried over sodium and distilled. Triethylamine was dried over molecular sieves (3 Å). *n*-Pentane used for chromatography was of technical grade and distilled before use. MilliQ water was obtained through purification by *MicroPure ultrapure*-System from TKA.

Sensitive reactions were performed under an argon atmosphere using dried solvents and flame-dried glassware. Microwave reactions were performed using the *Microwave Accelerated Reaction System*, Model MARS®, from CEM Corporation. For this, the *ramp-to-temperature* method was selected using 400 W at the given temperature and time. Sonications were conducted using *Sonorex SUPER RK 514 BH* from Bandelin Electronics. Freeze dryings were performed on *ALPHA 1-2* from Christ. For this, compounds were dispersed in distilled water and frozen in liquid nitrogen under rotation.

CHROMATOGRAPHY

Thin-layer chromatography (TLC) was used for reaction monitoring on *POLYGRAM® SIL G/UV254* plates (0.2 mm) from *Macherey-Nagel*. Spots were visualized by a UV-handlamp (254 nm, 365 nm and 395 nm) by *Herolab* or *AHOME*. Flash-column chromatography was carried out under argon on silica gel *MN 60 M* (40–63 µm) from *Macherey-Nagel*. For this, the crude materials were concentrated over *Celite® 545* from *Sigma Aldrich*. The purity was determined by analytical high performance liquid chromatography (HPLC) equipped with a *NUCLEODUR 100-5* normal phase column (inner diameter 4.6 mm, length 250 mm, silica gel, particle size 5 µm) by *Macherey Nagel*. The setup consists of a *Gastorr* AG-32 degasser, binary *Waters 1525 pump* with a flow of 1 mL/min, a *Waters 717plus autosampler* and a *Waters 2487 UV/Vis detector* (270 nm). Chromatograms were analyzed using the software *Breeze* (3.20) by *Waters*. HPLC-grade *n*-hexane and ethyl acetate were used with the following gradient run: *n*-hexane/ethyl acetate 90/10 → 0/100 over 30 min, holding 0/100 for 5 minutes. The samples were dissolved in DCM (1 mM) and filtered using a syringe filter (0.22 µM, PTFE).

GENERAL ANALYTICAL METHODS

FT-IR spectra were measured on *a FT/IR-4600* (*Jasco*). For this, concentrated DCM solutions of the compounds were drop-casted. High resolution mass spectra were recorded on a *Bruker maXis 4G* (*Q-TOF*) *via* electrospray-ionization. Samples were dissolved in dichloromethane and injected *via* flow-injection. Melting points were determined by using a *BÜCHI B-540.* NMR spectra were recorded at room temperature on a *AVNEO400* (^{1}H: 400 MHz, ^{13}C: 101 MHz, ^{19}F: 376 MHz) spectrometer from *Bruker* and analysed using *MestReNova* v15.01-35756. Analytical data are given with the respective frequency, solvent, temperature, chemical shift δ [ppm], multiplicity, integral, and assignment. The abbreviation of the fine structure is: s = singlet, d = doublet, t = triplet, q = quartet, m = multiplet, dd = doublet of doublet, tt = triplet of triplet, br = broad. Coupling constants $^{n}J_{XY}$ are given in Hertz [Hz], with *n* describing the number of bonds between coupling nuclei X and Y. For internal referencing, the residual proton signals of $CDCl_3$ (^{1}H: 7.26 ppm, ^{13}C: 77.16 ppm) or DMSO-d_6 (^{1}H: 2.50 ppm, ^{13}C: 39.52 ppm) were used. Signals were assigned using conventional 2D-NMR methods (HSQC, HMBC, COSY). ζ-potential and dynamic light scattering (DLS) were measured using a *Zetasizer Nano-ZS* (*Malvern Pananalytical* with HeNe-Laser (633 nm) at room temperature. Samples were measured in folded capillary cells (DTS1070) in triplicate.

X-RAY DIFFRACTOMETRY

Crystals of the compounds were grown by slow evaporation of concentrated DCM solutions (**O_4**), partially overlayered with cyclohexane (**N_3O_1**), methanol (**N_4**), or ethanol (**N_2O_2**). The crystals were mounted on nylon loops in inert oil. Data were collected on a *Bruker AXS D8 Venture* diffractometer

with *Photon II* detector (mono-chromated $Cu_{K\alpha}$ radiation, λ = 1.54178 Å, microfocus source) at 100(2) K. The structures were solved by *Direct Methods* (*SHELXS-2013*)[1] and refined anisotropically by full-matrix least-squares on F2 (SHELXL-2017).[2–4] Absorption corrections were performed semi-empirically from equivalent reflections on basis of multi-scans (*Bruker AXS APEX3*). Hydrogen atoms were refined using a riding model or rigid methyl groups. The molecule in **N_2O_2** is disordered over a mirror plane. Two atoms are only slightly off the mirror and lead to unrealistic displacement parameters when placed on the special position. To overcome this, the local symmetry was ignored in the refinement (negative PART) and the whole molecule was refined as disordered over the special position with an occupancy of 50%. Lowering the symmetry by removing the mirror plane (resulting in space group $Pna2_1$) does not resolve the disorder. RIGU restraints were applied to all displacement parameter to avoid correlations. Considering the vast disorder quantitative results should be carefully assessed and may be unreliable. CCDC 2393405-2393408 contain the supplementary crystallographic data for this paper. These data can be obtained free of charge from the Cambridge Crystallographic Data Centre via http://www.ccdc.cam.ac.uk/data_request/cif.

OPTICAL SPECTROSCOPY

UV-Vis spectra were measured on a *Jasco V-550* spectrophotometer with baseline correction. Excitation and emission spectra were recorded on a RF-6000 spectrometer from Shimadzu Corporation. Diluted samples were prepared in the *Semi-Micro* quartz cuvettes (1.4 mL, 10x4 mm) from *Hellma Analytics*. Solid samples were placed between two quartz glass slides in 45° orientation to the excitation and emission slits. Appropriate long-pass filters were used to suppress the lamp signals. Measurements were conducted at room temperature. The compounds **N_3O_1** and **N_4** showed photodegradation upon irradiation in dilute solutions. Hence, all solutions were purged with argon for 10 minutes before respective measurements. Absolute photoluminescence (PL) quantum yields were measured with the stand-alone absolute PL quantum yield measurement system C9920-02 by *Hamamatsu Photonics* equipped with an integrating sphere, an L9799-01 CW Xe light source (150 W), a monochromator, and a C7473 photonic multi-channel analyzer. The data were analyzed using the U6039-05 software (*Hamamatsu Photonics*, Ltd., Shizuoka, Japan). Steady-state excitation and emission spectra were recorded on a *FluoTime 300* spectrometer from *PicoQuant* equipped with a 300 W ozone-free Xe lamp (250-900 nm), a 10 W Xe flash-lamp (250-900 nm, pulse width ca. 1 µs) with repetition rates of 0.1 – 300 Hz, double grating excitation monochromators (Czerny-Turner type, grating with 1200 lines/mm, blaze wavelength: 300 nm), diode lasers (pulse width < 80 ps) operated by a computer-controlled laser driver PDL-828 “Sepia II” (repetition rate up to 80 MHz, burst mode for slow and weak decays), two double-grating emission monochromators (Czerny-Turner, selectable gratings blazed at 500 nm with 2.7 nm/mm dispersion and 1200 lines/mm, or blazed at 1200 nm with 5.4 nm/mm dispersion and 600 lines/mm) with adjustable slit width between 25 µm and 7 mm, Glan-Thompson polarizers for excitation (after the Xe-lamps) and emission (after the sample). Different sample holders (Peltier-cooled mounting unit ranging from -15 to 110 °C or an adjustable front-face sample holder), along with two detectors (namely a PMA Hybrid-07 from *PicoQuant* with transit time spread FWHM < 50 ps, 200 – 850 nm, or a H10330C-45-C3 NIR detector with transit time spread FWHM 0.4 ns, 950-1400 nm from *Hamamatsu*) were used. Steady-state spectra and photoluminescence lifetimes were recorded in TCSPC mode by a *PicoHarp 300* (minimum base resolution 4 ps) or in MCS mode by a *TimeHarp 260* (where up to several ms can be traced). Emission and excitation spectra were corrected for source intensity (lamp and grating) by standard correction curves. For samples with lifetimes in the ns order, an instrument response function calibration (IRF) was performed using a diluted *Ludox*® dispersion. Lifetime analysis was performed using the commercial *EasyTau 2* software (*PicoQuant*). The quality of the fit was assessed by minimizing the reduced chi squared function (χ^2) and visual inspection of the weighted residuals and their autocorrelation. All solvents used were of spectrometric grade (*Uvasol*®, *Merck*). CIE coordinates were constructed using an *OriginLab*^{TM} Plugin (Chromaticity Diagram, File Version 1.20, Licence: Free).

PHOTOGRAPHS

Photographs of the samples were taken with a *Canon EOS R10* with a *Canon 18-150* lens*.* The crystals were photographed using a *Olympus SZX16* microscope equipped with a *Olympus KL 1500 LCD* lamp, a *Bresser MikroCam II Full HD HSP* camera, and UV light from *StarLight Opto-Electronics* (type *TC 250*).

POLYMETHYLMETHACRYLATE (PMMA) FILMS

PMMA films were prepared with the spin-coating method using the *SCV-10* by *Schaefer-Tec* and the *LABOPORT® N96* pump by *KNF*. First, 120 mg PMMA were dissolved in 1.2 mL DCM. 1.1 mL of this solution were added to 1.1 mg compound and sonicated. 0.5 mL of this solution were added quickly with a Hamilton-syringe to a glass slide (50x50x1.55 mm) placed on the spin-coater table, which accelerated in 3 s to 10 rps. After 20 s, another 0.5 mL of the PMMA-compound-solution were added, and the process repeated. Afterwards, the PMMA films were dried at ambient conditions overnight.

3D-PRINTING (STEREOLITHOGRAPHY, SLA)

For the 3D-printed materials, resins were prepared consisting of 1 wt% Diphenyl(2,4,6-trimethylbenzoyl)phosphine oxide as the initiator, 30 wt% Poly(ethylenglycol)-dimethacrylat as a linker, and 69 wt% of 2-[[(Butylamino)carbonyl]oxy]ethylacrylat as the monomer. To these resins, 0.1 wt% compound dissolved in a minimal amount of DCM was added and thoroughly mixed. Afterwards, DCM was evaporated and the resins photopolymerized using a *Photon Mono 4K* by *Anycubic*. The parameters applied were 23 s normal exposure time, 1 s off time, 23 s bottom exposure time, 0.1 mm layer thickness, and 1 bottom layer. Then, the printed objects were washed with isopropanol.

COMPUTATIONAL DETAILS

Theoretical calculations were performed using *Gaussian 16*.[5] The compound geometries in the energetic ground states were calculated by using PBE0/TZVP[6,7] with the Grimme dispersion correction (GD3BJ).[8] For this, the methods were modified with *opt=tight* and *int=(ultrafine)* keywords to ensure that the four convergence criteria of *Gaussian* are fulfilled. Due to the large number of potential conformers (equatorial or axial position of the methyl groups), the optimized structures of all-axial and all-equatorial conformers were compared. Frequency analysis revealed no imaginary frequencies, indicating that the obtained structures are stationary points. The polarizable continuum model (PCM) was applied with dimethyl sulfoxide as the solvent. To simulate UV/Vis-spectra, the first 40 transitions were calculated using the *TD* keyword.[9] The optimized first excited state geometries were obtained by using TD-PBE0/TZVP. Natural transition orbital (NTO) pairs and electron-density differences were calculated by means of PBE0/TZVP.

2 SYNTHETIC PROCEDURES

GENERAL PROCEDURES

GENERAL PROCEDURE (GP1): MICROWAVE-ASSISTED NUCLEOPHILIC AROMATIC SUBSTITUTION

In an argon-flushed 30 mL microwave-vessel, difluoro-electrophile (1 eq.), nucleophile (1.25 eq.) and caesium carbonate (2 eq.) were suspended in anhydrous *N,N*-dimethylformamide. The vessel was placed in the microwave and stirred at 120 °C until full consumption of the electrophile (TLC-control). Then, the mixture was transferred into a round-bottom flask and concentrated over Celite®. The residue was purified by column chromatography and lyophilized.

OVERVIEW OF PERFORMED SYNTHESES

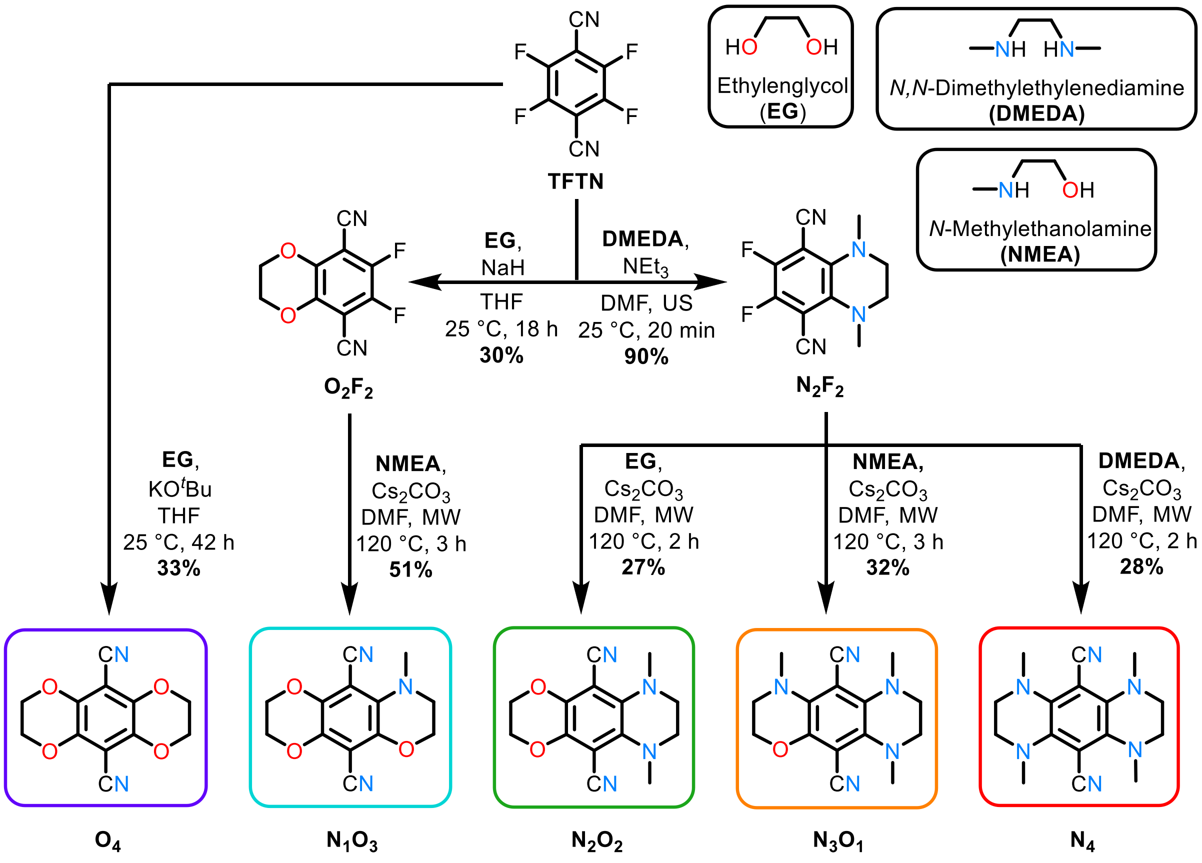

Figure S1: Synthesis of the target compounds.

SYNTHETIC PROCEDURES

6,7-DIFLUORO-2,3-DIHYDRO-1,4-BENZODIOXIN-5,8-DICARBONITRILE (O_2F_2)

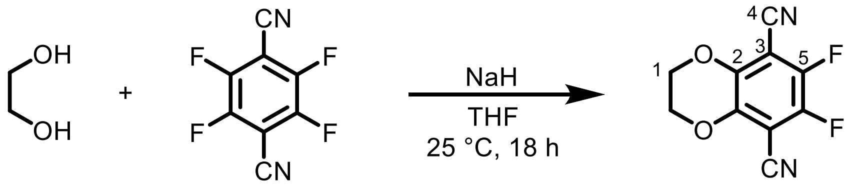

O_2F_2

In a flame-dried Schlenk flask, ethylene glycol (0.622 g, 10.0 mmol, 1 eq.) was dissolved in 50 mL of anhydrous tetrahydrofuran under argon atmosphere. Sodium hydride (60% in mineral oil, 0.900 g, 22.5 mmol, 2.2 eq.) was added and the mixture stirred for 30 minutes. Then, tetrafluoroterephthalonitrile (**TFTN**, 2.00 g, 10.0 mmol, 1 eq.) was added and the mixture stirred overnight at room temperature. Full consumption of **TFTN** was observed by TLC (DCM/*n*-pentane 1/1). The reaction was terminated by careful addition of brine (100 mL) and the crude product extracted with ethyl acetate three times. The organic layers were combined, dried over magnesium sulfate, filtered and concentrated over Celite®. Column chromatography (6x15 cm, DCM/*n*-pentane 2/1) yielded the product **O_2F_2** as a white solid (0.674 g, 3.03 mmol, 30.3%), which eluted as the third band with dark blue fluorescence under irradiation with 365 nm.

M($C_{10}H_4F_2N_2O_2$): 222.15 g/mol.

Although **O_2F_2** is literature known,[10] no NMR characterization has yet been reported.

Notes: **O_2F_2** sublimes *in vacuo* (5 mbar, 60 °C).

^{1}H-NMR (400 MHz, $CDCl_3$, 298 K): δ [ppm] = 4.48 (s, 4H, C*H*$_2$).

^{13}C{^{1}H}-NMR (101 MHz, $CDCl_3$, 298 K): δ [ppm] = 144.8 (dd, $^1J_{C\text{-}F}$ = 258.7 Hz, $^2J_{C\text{-}F}$ = 16.0 Hz, *C*5), 142.4 (t, $^3J_{C\text{-}F}$ = 3.4 Hz, *C*2), 108.7 (t, $^3J_{C\text{-}F}$ = 2.4 Hz, *C*4), 97.2 (dd, $^2J_{C\text{-}F}$ = 11.4 Hz, $^3J_{C\text{-}F}$ = 7.4 Hz, *C*3), 65.1 (*C*1).

^{19}F-NMR (376 MHz, $CDCl_3$, 298 K): δ [ppm] = -137.41.

HR-MS (ESI-pos, 70 eV): m/z = 223.0314 [M + H]$^+$, calculated for [$C_{10}H_4F_2N_2O_2$ + H]$^+$ = 223.0314; 245.0133 [M + Na]$^+$, calculated for [$C_{10}H_4F_2N_2O_2$ + Na]$^+$ = 245.0133;

IR: $\tilde{\nu}$ [cm^{-1}] = 3655, 2981, 2878, 2238, 1496, 1476, 1460, 1379, 1371, 1319, 1281, 1247, 1154, 1102, 1062, 965, 951, 888, 854, 811, 764, 749, 702, 651, 626, 595, 539, 482, 435, 411.

6,7-DIFLUORO-1,2,3,4-TETRAHYDRO-1,4-DIMETHYL-5,8-QUINOXALINEDICARBONITRILE (N_2F_2)

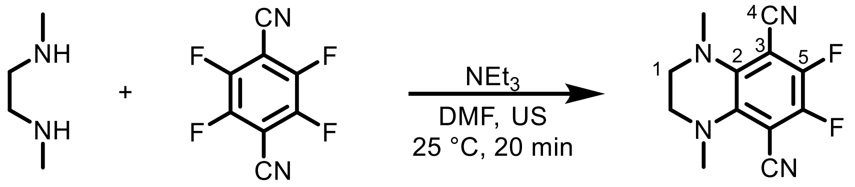

N_2F_2

In a Schlenk flask, tetrafluoroterephthalonitrile (**TFTN**, 3.00 g, 15.0 mmol, 1 eq.) was dissolved in 80 mL of anhydrous *N,N*-dimethylformamide under argon atmosphere. *N,N*-Dimethylethylenediamine (1.61 mL, 1.30 g, 14.8 mmol, 1 eq.) and triethylamine (4.18 mL, 3.03 g, 30.0 mmol, 2 eq.) were added and the mixture stirred for 20 minutes in an ultrasonic bath (37 kHz, 200 W). Full consumption of **TFTN** was observed by TLC (DCM). The reaction mixture was concentrated *in vacuo*. Column chromatography (6x35 cm, DCM/*n*-pentane 3/1) yielded the product **N_2F_2** as a yellow solid (3.36 g, 13.6 mmol, 90.0%).

M($C_{12}H_{10}F_2N_4$): 248.24 g/mol.

^{1}H-NMR (400 MHz, DMSO-*d*$_6$, 298 K): δ [ppm] = 3.28 (s, 4H, C*H$_2$*), 3.20 (s, 6H, C*H$_3$*).

^{19}F-NMR (376 MHz, DMSO-*d*$_6$, 298 K): δ [ppm] = -147.18.

Spectroscopic data match literature values.[11]

2,3,7,8-TETRAHYDROBENZO[1,2-B:4,5-B']BIS([1,4]DIOXINE)-5,10-DICARBONITRILE (O$_4$)

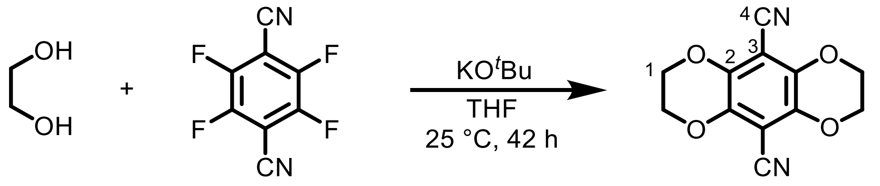

O$_4$

In a flame-dried Schlenk flask, ethylene glycol (0.111 g, 1.79 mmol, 1 eq.) was dissolved in 20 mL of anhydrous tetrahydrofuran under argon atmosphere. Potassium *tert*-butoxide (1.30 g, 10.7 mmol, 6 eq.) was added and the mixture stirred for 30 minutes. Then, tetrafluoroterephthalonitrile (**TFTN**, 0.352 g, 1.76 mmol, 1 eq.) was added and the mixture stirred for 42 h at room temperature. Full consumption of **TFTN** was observed by TLC (DCM). The reaction mixture was concentrated *in vacuo*. Column chromatography (4.7x20 cm, DCM/*n*-pentane 2/1 → 100/0) yielded the product **O$_4$** as an off-white solid (0.0710 g, 0.291 mmol, 33.0%) after freeze-drying.

M($C_{12}H_8N_2O_4$): 244.21 g/mol.

Melting point: > 400 °C (decomposition).

^{1}H-NMR (400 MHz, DMSO-*d*$_6$, 298 K): δ [ppm] = 4.41 (s, 8H, C*H$_2$*).

^{13}C{^{1}H}-NMR (101 MHz, DMSO-*d*$_6$, 298 K): δ [ppm] = 139.3 (*C*2), 111.7 (*C*4), 94.0 (*C*3), 64.9 (*C*1).

HR-MS (ESI-pos, 70 eV): m/z = 245.0558 [M + H]$^+$, calculated for [$C_{12}H_8N_2O_4$ + H]$^+$ = 245.0557; 267.0377 [M + Na]$^+$, calculated for [$C_{12}H_8N_2O_4$ + Na]$^+$ = 267.0376.

IR: $\tilde{\nu}$ [cm^{-1}] = 2981, 2970, 2889, 2884, 1758, 1473, 1381, 1274, 1267, 1258, 1251, 1241, 1151, 953, 750.

9-METHYL-3,7,8,9-TETRAHYDRO-2H-[1,4]DIOXINO[2',3':4,5]BENZO[1,2-B][1,4]OXAZINE-5,10-DICARBONITRILE (N$_1$O$_3$)

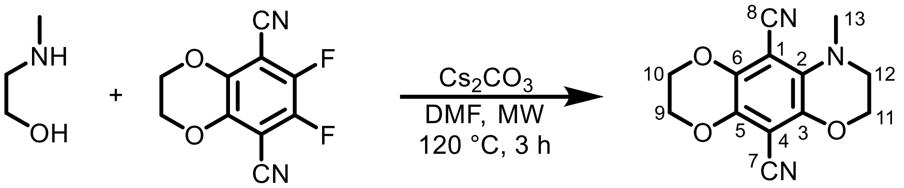

N$_1$O$_3$

N$_1$O$_3$ was synthesized according to **GP1** using **O$_2$F$_2$** (0.521 g, 2.35 mmol, 1 eq.), *N*-methylethanolamine (0.937 mL, 0.220 g, 2.93 mmol, 1.25 eq.), caesium carbonate (1.91 g, 5.85 mmol, 2 eq.) and anhydrous *N,N*-dimethylformamide (20 mL). Column chromatography (6x40 cm, DCM/acetone 100/0 → 100/0.5 → 100/1) yielded the product **N$_1$O$_3$** as a yellow solid (0.308 g, 1.20 mmol, 51.0%) after freeze-drying.

M($C_{13}H_{11}N_3O_3$): 257.25 g/mol.

Melting point: 292 °C.

^{1}H-NMR (400 MHz, CDCl$_3$, 298 K): δ [ppm] = 4.36 (s, 4H, *H*9, *H*10), 4.24 (m, 2H, H*11*), 3.24 (m, 2H, *H*12), 3.12 (s, 3H, *H*13).

$^{13}C\{^1H\}$-NMR (101 MHz, CDCl$_3$, 298 K): δ [ppm] = 143.2 (*C*3), 140.1 (*C*5/*C*6), 138.5 (*C*6/*C*5), 133.8 (*C*2), 114.1 (*C*8/*C*7), 112.1 (*C*7/*C*8), 96.6 (*C*1/*C*4), 95.3 (*C*4/*C*1), 65.0 (*C*9/*C*10), 64.9 (*C*10/*C*9), 62.4 (*C*11), 49.7 (*C*12), 44.0 (*C*13).

HR-MS (ESI-pos, 70 eV): m/z = 258.0877 $[M + H]^+$, calculated for $[C_{13}H_{12}N_3O_3 + H]^+$ = 258.0873; 280.0697 $[M + Na]^+$, calculated for $[C_{13}H_{12}N_3O_3 + Na]^+$ = 280.0693.

IR: $\tilde{\nu}$ [cm^{-1}] = 3655, 3647, 2994, 2981, 2971, 2915, 2902, 2889, 2884, 2225, 2217, 1770, 1758, 1473, 1462, 1446, 1433, 1380, 1298, 1273, 1247, 1241, 1218, 1155, 1098, 1079, 1059, 1011, 1002, 955, 945, 909, 891, 878, 871, 860, 856, 850, 806, 764, 750, 650, 592, 539, 528, 519, 514, 508.

Spectroscopic data match literature values.[12]

6,9-DIMETHYL-2,3,6,7,8,9-HEXAHYDRO-[1,4]DIOXINO[2,3-G]QUINOXALINE-5,10 DICARBONITRILE (N_2O_2)

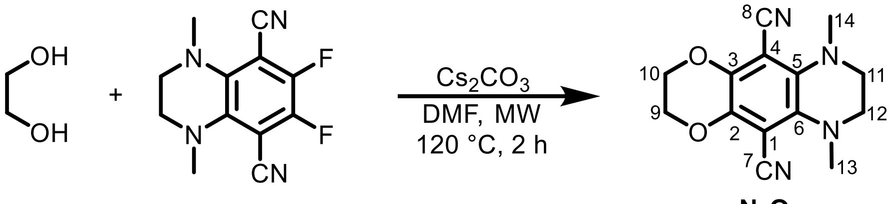

N_2O_2

N_2O_2 was synthesized according to **GP1** using **N_2F_2** (0.600 g, 2.42 mmol, 1 eq.), ethylene glycol (0.168 mL, 0.186 g, 3.00 mmol, 1.25 eq.), caesium carbonate (2.17 g, 6.65 mmol, 2.2 eq.) and anhydrous *N,N*-dimethylformamide (20 mL). Column chromatography (6x40 cm, DCM/acetone 100/0 → 100/1 → 100/2) yielded the product **N_2O_2** as a yellow solid (0.176 g, 0.651 mmol, 26.9%) after freeze-drying.

M($C_{14}H_{14}N_4O_2$): 270.29 g/mol.

Melting point: 239 °C.

^1H-NMR (400 MHz, CDCl$_3$, 298 K): δ [ppm] = 4.35 (s, 4H, *H*9, *H*10), 3.11 (s, 4H, *H*11, *H*12), 3.10 (s, 6H, *H*13, *H*14).

$^{13}C\{^1H\}$-NMR (101 MHz, CDCl$_3$, 298 K): δ [ppm] = 139.0 (*C*2, *C*3), 137.9 (*C*5, *C*6), 114.8 (*C*8, *C*7), 96.5 (*C*1, *C*4), 65.0 (*C*9, *C*10), 46.9 (*C*11, *C*12), 44.5 (*C*13, *C*14).

HR-MS (ESI-pos, 70 eV): m/z = 271.1191 $[M + H]^+$, calculated for $[C_{14}H_{14}N_4O_2 + H]^+$ = 271.1190; 293.1010 $[M + Na]^+$, calculated for $[C_{14}H_{14}N_4O_2 + Na]^+$ = 293.1009.

IR: $\tilde{\nu}$ [cm^{-1}] = 2981, 2971, 2932, 2909, 2902, 2888, 2883, 2221, 1769, 1758, 1474, 1459, 1456, 1445, 1420, 1382, 1300, 1273, 1247, 1241, 1150, 1144, 1137, 1132, 1115, 1093, 1061, 1051, 982, 965, 953, 829, 764, 751, 419.

Spectroscopic data match literature values.[13]

4,6,9-TRIMETHYL-3,4,6,7,8,9-HEXAHYDRO-2H-[1,4]OXAZINO[2,3-G]QUINOXALINE-5,10-DICARBONITRILE (N_3O_1)

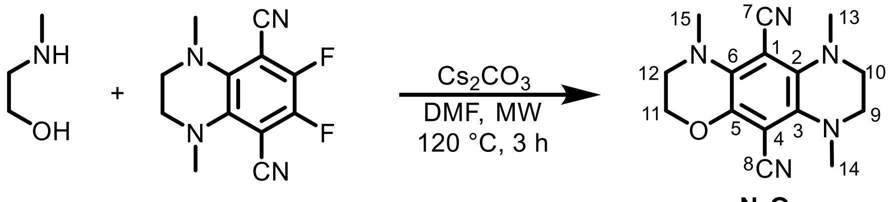

N_3O_1

N_3O_1 was synthesized according to **GP1** using **N_2F_2** (0.583 g, 2.35 mmol, 1 eq.), *N*-methylethanolamine (0.235 mL, 0.220 g, 2.93 mmol, 1.25 eq.), caesium carbonate (1.98 g, 6.09 mmol, 2.1 eq.) and anhydrous *N,N*-dimethylformamide (20 mL). Column chromatography (6x40 cm, DCM/acetone 100/0 → 100/1 → 100/2 → 100/4) yielded the product **N_3O_1** as an orange solid (0.214 g, 0.755 mmol, 32.1%) after freeze-drying.

M($C_{15}H_{17}N_5O$): 283.34 g/mol.

Melting point: 196 °C.

^{1}H-NMR (400 MHz, $CDCl_3$, 298 K): δ [ppm] = 4.24 (m, 2H, *H*11), 3.18 (m, 2H, *H*12), 3.15 (m, 5H, NCH_2, NCH_3), 3.08 (m, 2H, NCH_2), 3.01 (2 s, 6H, NCH_3, NCH_3).

^{13}C{^{1}H}-NMR (101 MHz, $CDCl_3$, 298 K): δ [ppm] =
144.5 (*C*5), 138.9 (*C*2/*C*3), 138.3 (*C*3/*C*2), 131.8 (*C*6), 116.8 (*C*7/*C*8), 115.8 (*C*8/*C*7), 102.0 (*C*4/*C*1), 95.1 (*C*1/*C*4), 61.5 (*C*11), 49.8 (*C*12), 47.1 (CH_2), 46.7 (CH_2), 44.9 (CH_3), 44.7 (CH_3), 44.1 (CH_3).

HR-MS (ESI-pos, 70 eV): m/z = 283.1430 $[M + H]^+$, calculated for $[C_{15}H_{17}N_5O + H]^+$ = 283.1428; 306.1326 $[M + Na]^+$, calculated for $[C_{15}H_{17}N_5O + Na]^+$ = 306.1325.

IR: $\tilde{\nu}$ [cm^{-1}] = 3674, 3669, 3655, 3650, 3636, 2995, 2981, 2971, 2915, 2901, 2889, 2884, 2820, 2218, 1770, 1758, 1554, 1522, 1473, 1463, 1456, 1437, 1426, 1410, 1376, 1310, 1279, 1248, 1241, 1218, 1153, 1138, 1114, 1104, 1088, 1058, 1011, 996, 968, 957, 953, 853, 846, 833, 821, 815, 809, 764, 758, 750, 627, 541, 538, 534, 527, 515, 509, 505, 447, 440, 426, 419, 409.

1,4,6,9-TETRAMETHYL-1,2,3,4,6,7,8,9-OCTAHYDROPYRAZINO[2,3-G]QUINOXALINE-5,10 DICARBONITRILE (N_4)

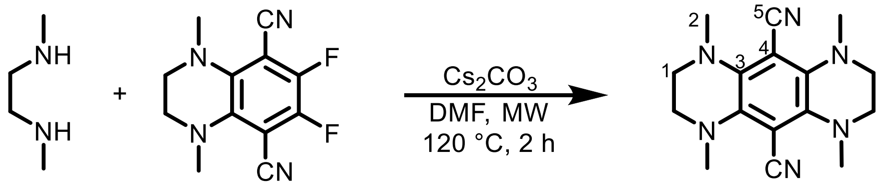

N_4

N_4 was synthesized according to **GP1** using **N_2F_2** (50.0 mg, 0.201 mmol, 1 eq.), *N,N*-Dimethylethylenediamine (108 µL, 88.8 mg, 1.01 mmol, 5 eq.), caesium carbonate (78.0 mg, 0.239 mmol, 1.2 eq.) and anhydrous *N,N*-dimethylformamide (10 mL). Column chromatography (3.3x35 cm, cyclohexane/ethyl acetate 1/1 → 1/2 → 1/4) yielded the product **N_4** as an orange solid (17.0 mg, 0.0574 mmol, 28.5%) after freeze-drying.

M($C_{16}H_{20}N_6$): 296.38 g/mol.

Melting point: 222 °C.

^{1}H-NMR (400 MHz, $CDCl_3$, 298 K): δ [ppm] = 3.11 (s, 8H, CH_2), 3.03 (s, 12H, CH_3).

^{13}C{^{1}H}-NMR (101 MHz, $CDCl_3$, 298 K): δ [ppm] = 138.5 (*C*3), 117.8 (*C*5), 99.8 (*C*4), 46.9 (*C*1), 44.9 (*C*2).

HR-MS (ESI-pos, 70 eV): m/z = 296.1747 $[M + H]^+$, calculated for $[C_{16}H_{20}N_6 + H]^+$ = 296.1744.

IR: $\tilde{\nu}$ [cm^{-1}] = 2981, 2971, 2934, 2931, 2888, 2884, 2212, 1769, 1758, 1473, 1462, 1451, 1440, 1435, 1394, 1382, 1362, 1319, 1309, 1275, 1267, 1251, 1213, 1180, 1155 1137, 1112, 1087, 1073, 1045, 1011, 966, 957, 954, 914, 870, 821, 764, 758, 750, 703, 579, 503, 482, 460, 452, 426, 419, 412.

NMR SPECTRA

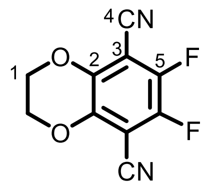

O_2F_2

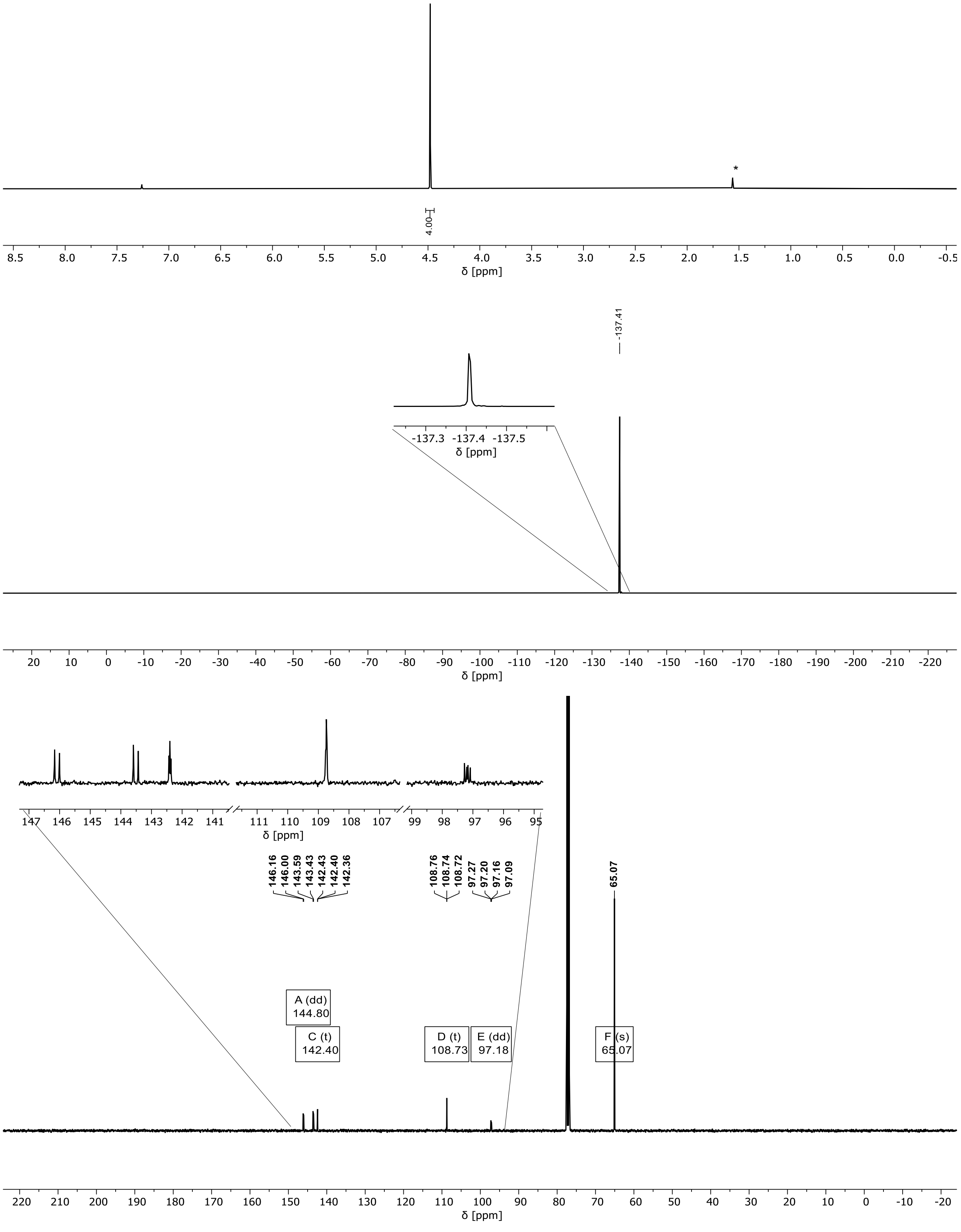

Figure S2: ^{1}H- (top, 400 MHz, $CDCl_3$, 298 K), ^{19}F- (middle, 376 MHz, $CDCl_3$, 298 K), and ^{13}C-NMR spectrum (bottom, 101 MHz, $CDCl_3$, 298 K) of compound **O_2F_2** (* = water).

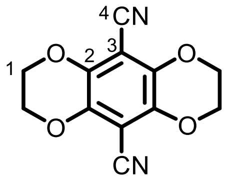

O$_4$

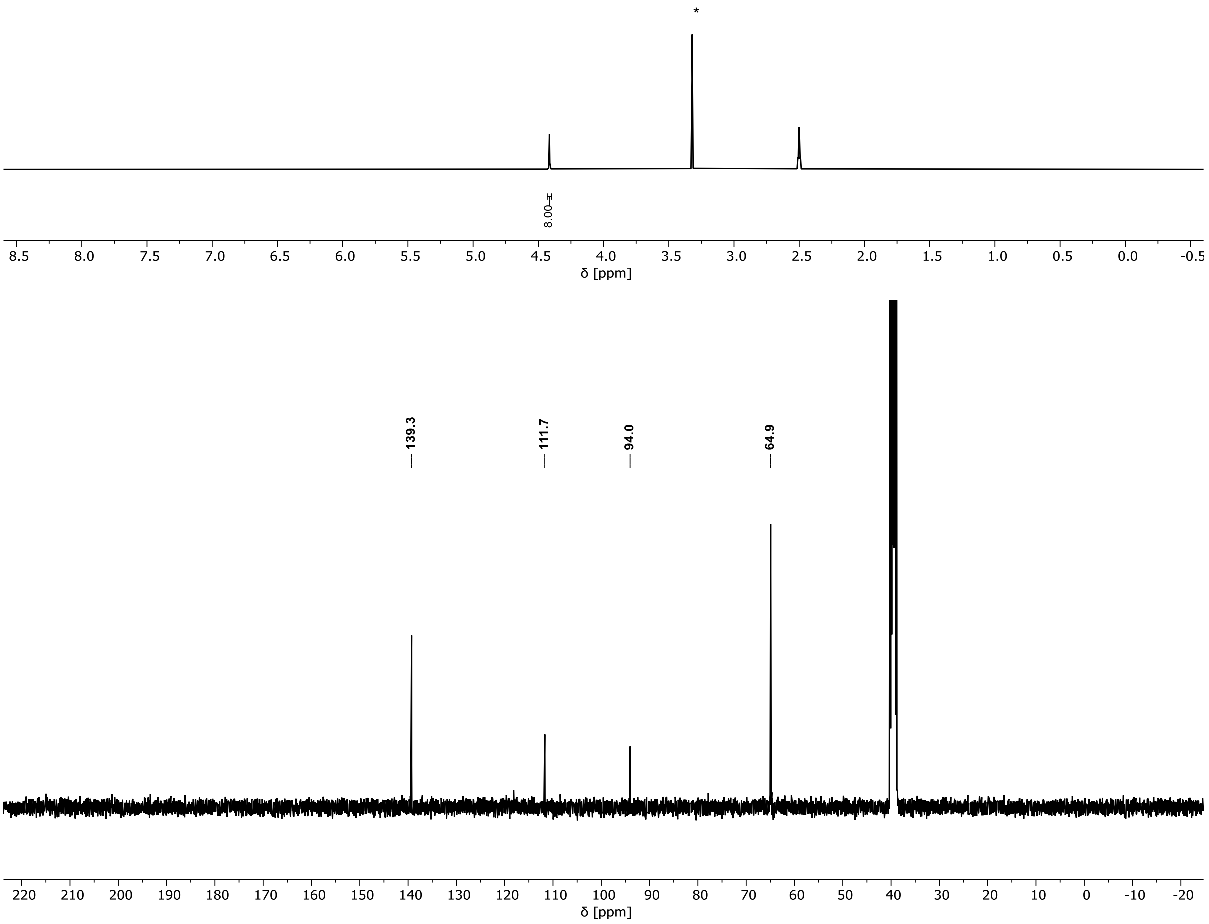

Figure S3: ^{1}H- (top, 400 MHz, DMSO-d$_6$, 298 K) and ^{13}C-NMR spectrum (bottom, 101 MHz, DMSO-d$_6$, 298 K) of compound **O$_4$** (* = water).

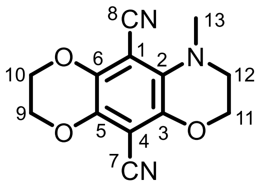

N_1O_3

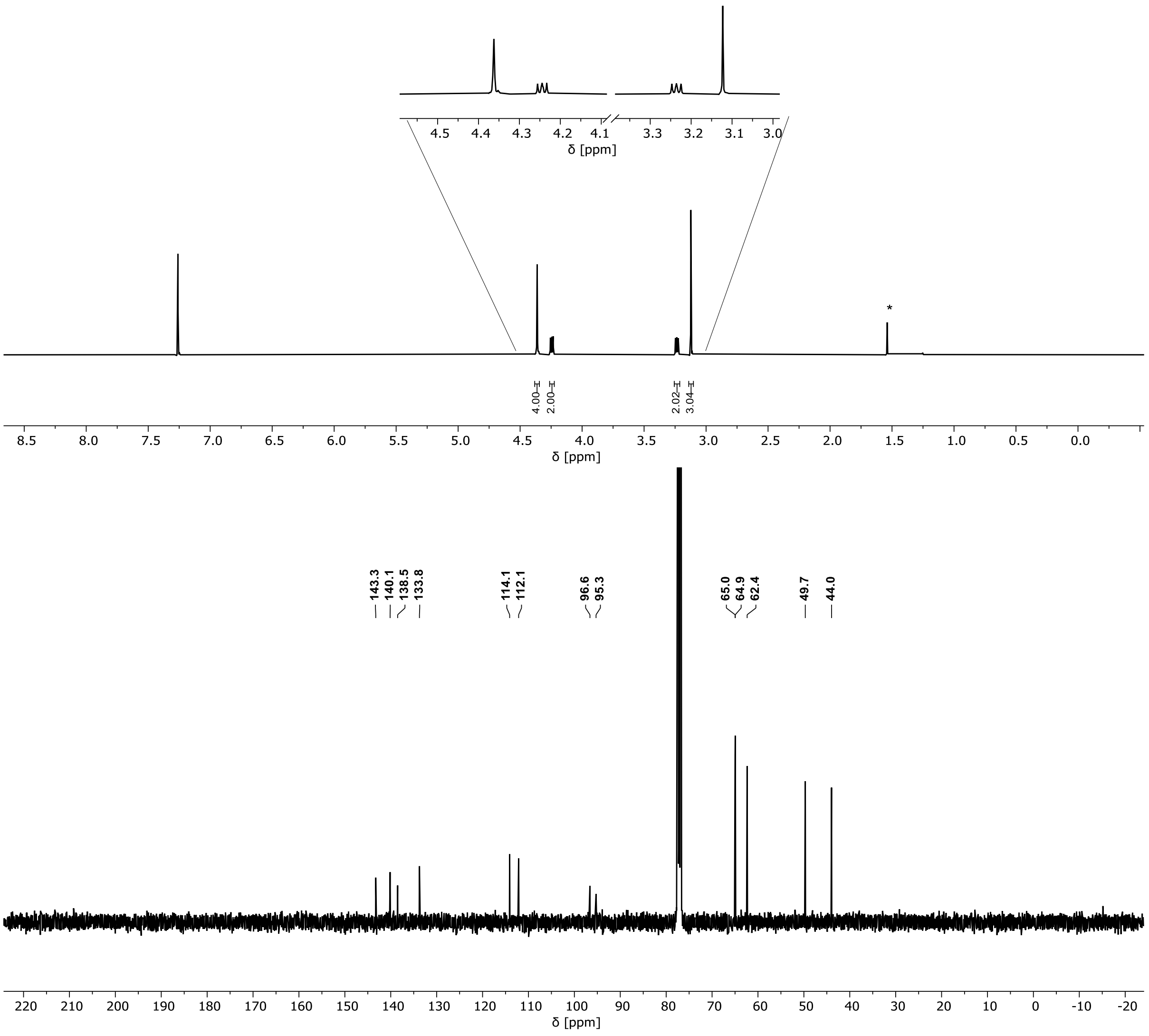

Figure S4: ^{1}H- (top, 400 MHz, $CDCl_3$, 298 K) and ^{13}C-NMR spectrum (bottom, 101 MHz, $CDCl_3$, 298 K) of compound **N_1O_3** (* = water).

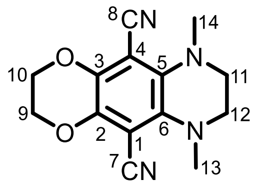

N_2O_2

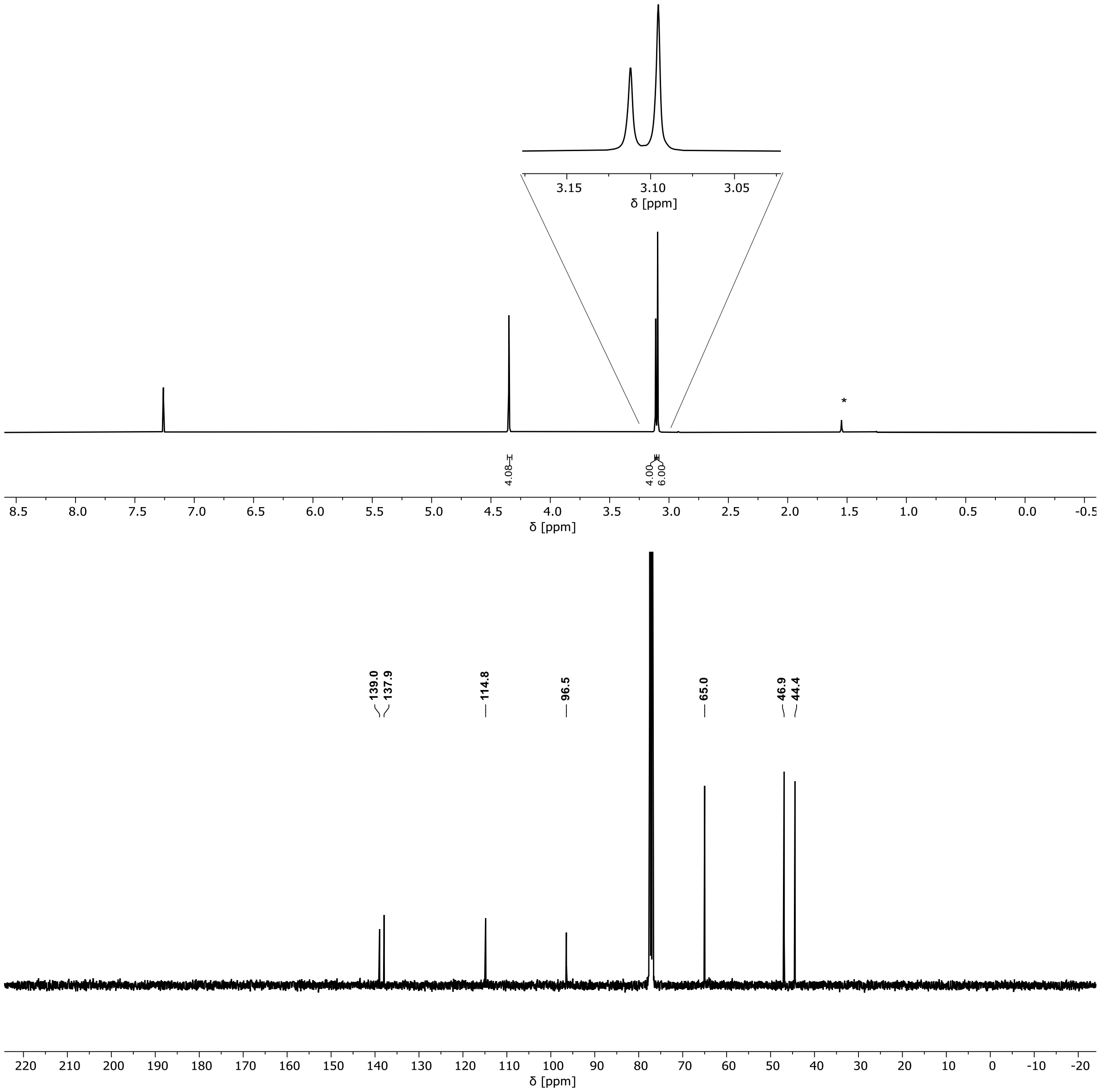

Figure S5: ^{1}H- (top, 400 MHz, $CDCl_3$, 298 K) and ^{13}C-NMR spectrum (bottom, 101 MHz, $CDCl_3$, 298 K) of compound **N_2O_2** (* = water).

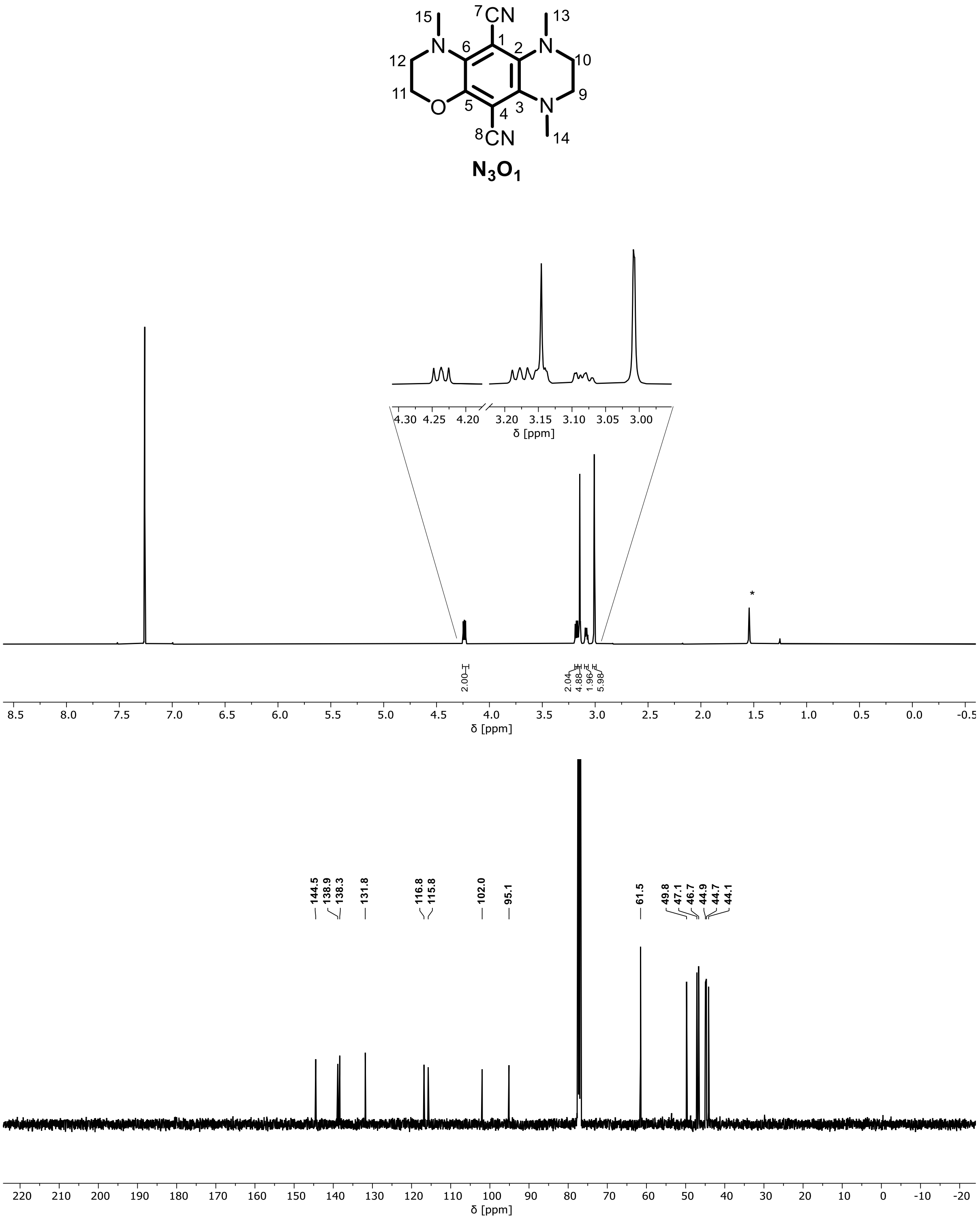

Figure S6: ^{1}H- (top, 400 MHz, $CDCl_3$, 298 K) and ^{13}C-NMR spectrum (bottom, 101 MHz, $CDCl_3$, 298 K) of compound **N_3O_1** (* = water).

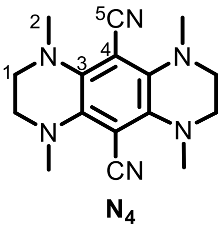

N4

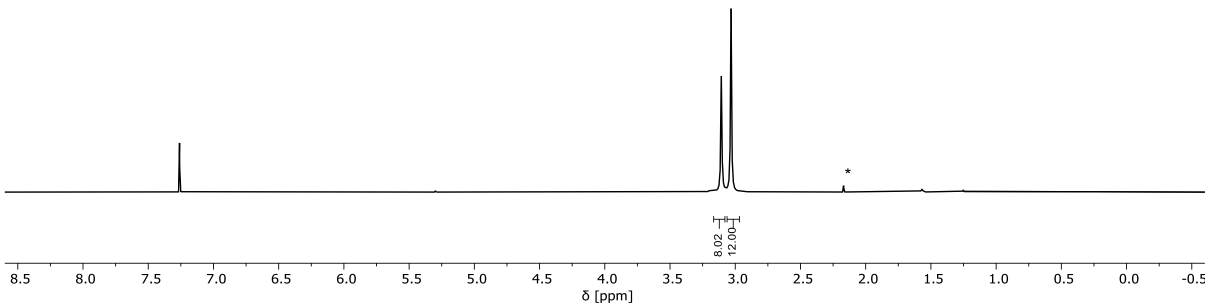

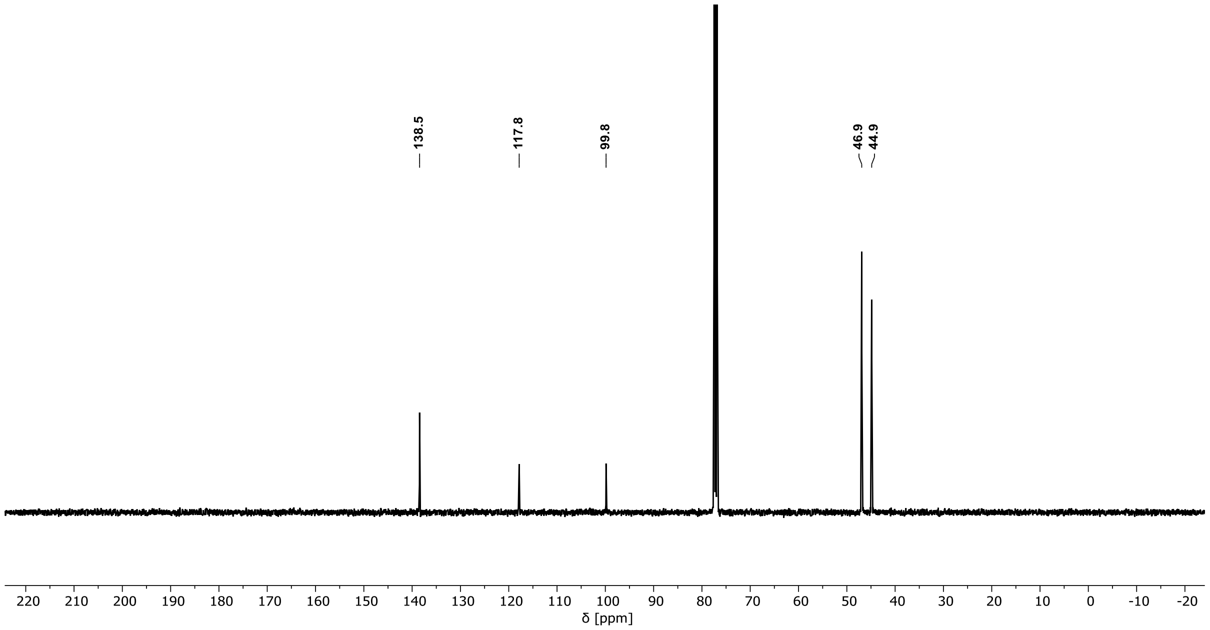

Figure S7: ^{1}H- (top, 400 MHz, $CDCl_3$, 298 K) and ^{13}C-NMR spectrum (bottom, 101 MHz, $CDCl_3$, 298 K) of compound **N4** (* = acetone).

HPLC CHROMATOGRAMS

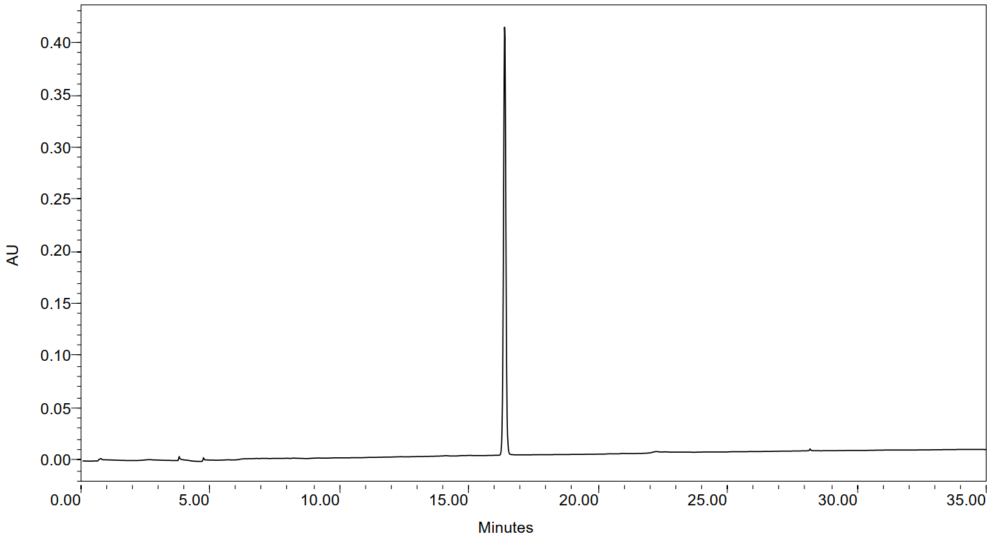

Figure S8: HPLC chromatogram of **O_4** (*n*-hexane/ethyl acetate 90/10 → 0/100 over 35 min).

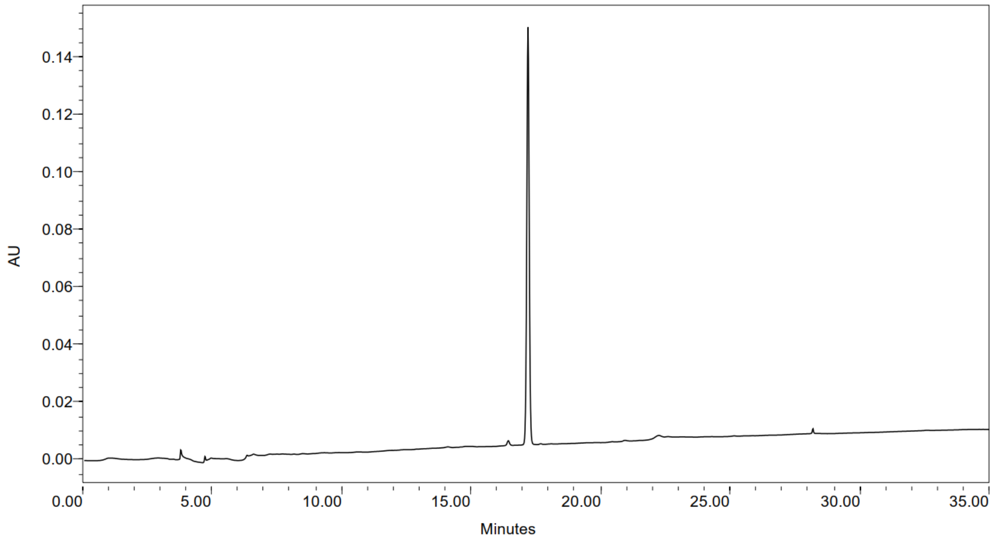

Figure S9: HPLC chromatogram of **N_1O_3** (*n*-hexane/ethyl acetate 90/10 → 0/100 over 35 min).

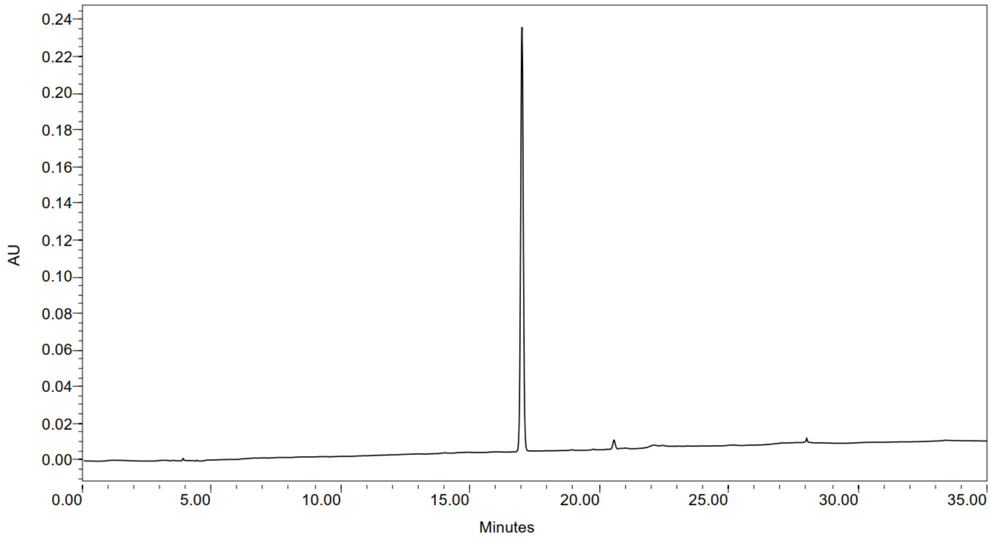

Figure S10: HPLC chromatogram of **N_2O_2** (*n*-hexane/ethyl acetate 90/10 → 0/100 over 35 min).

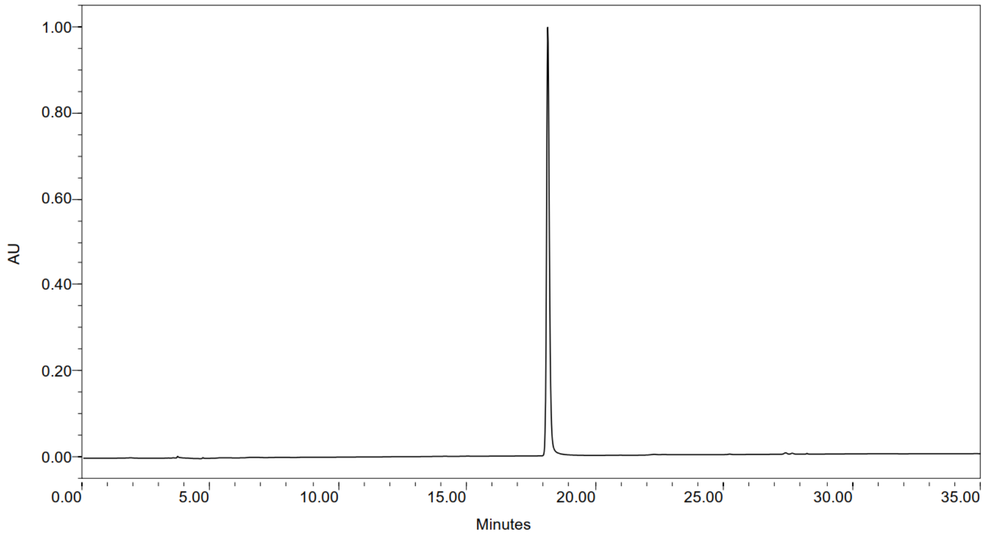

Figure S11: HPLC chromatogram of **N_3O_1** (*n*-hexane/ethyl acetate 90/10 → 0/100 over 35 min).

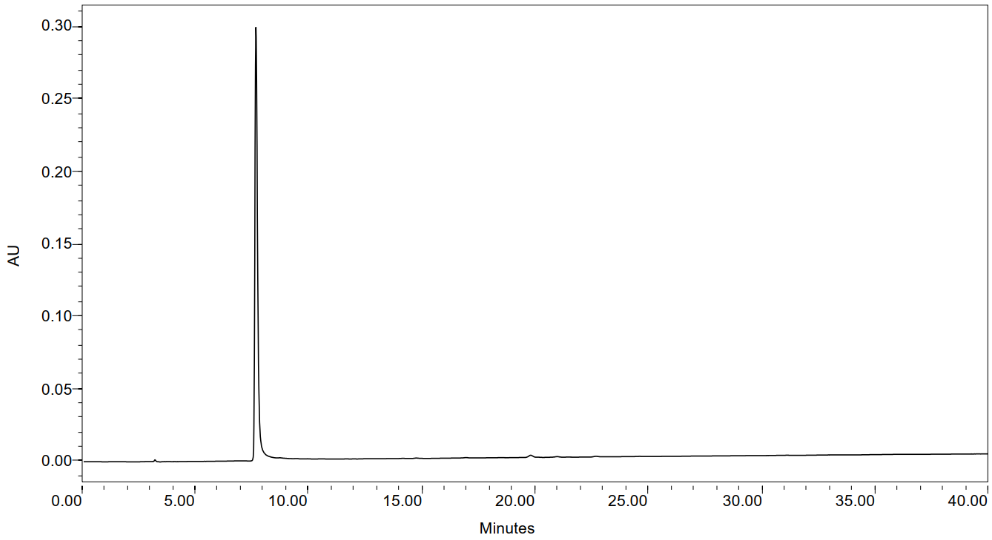

Figure S12: HPLC chromatogram of **N_4** (*n*-hexane/ethyl acetate 50/50 → 0/100 over 40 min).

3 PHOTOPHYSICAL PROPERTIES

UV/VIS ABSORPTION SPECTRA

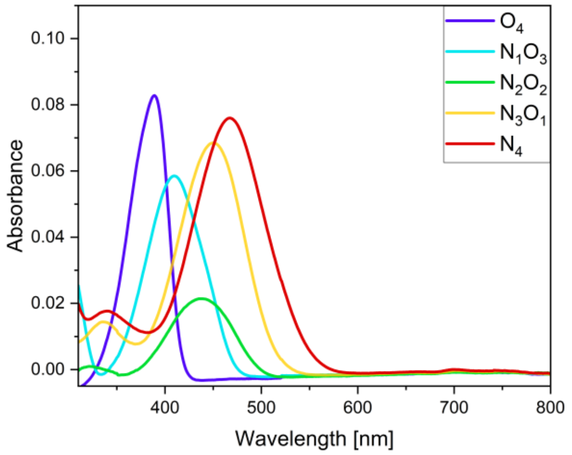

Figure S13: UV/Vis absorption spectra of all compounds in DMSO (10 µM).

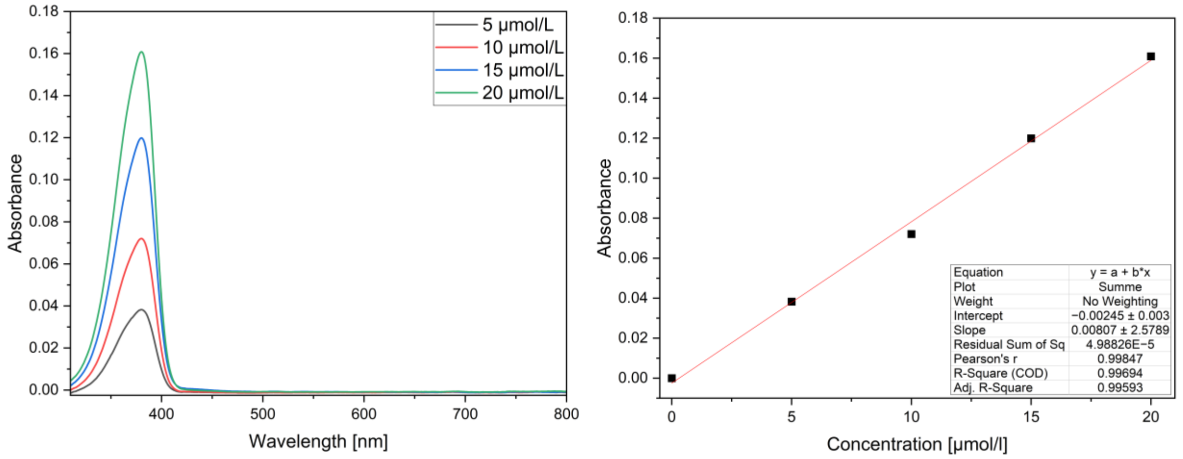

Figure S14: UV/Vis absorption spectra of $\mathbf{O_4}$ in THF at different concentrations (left) and regression analysis (right).

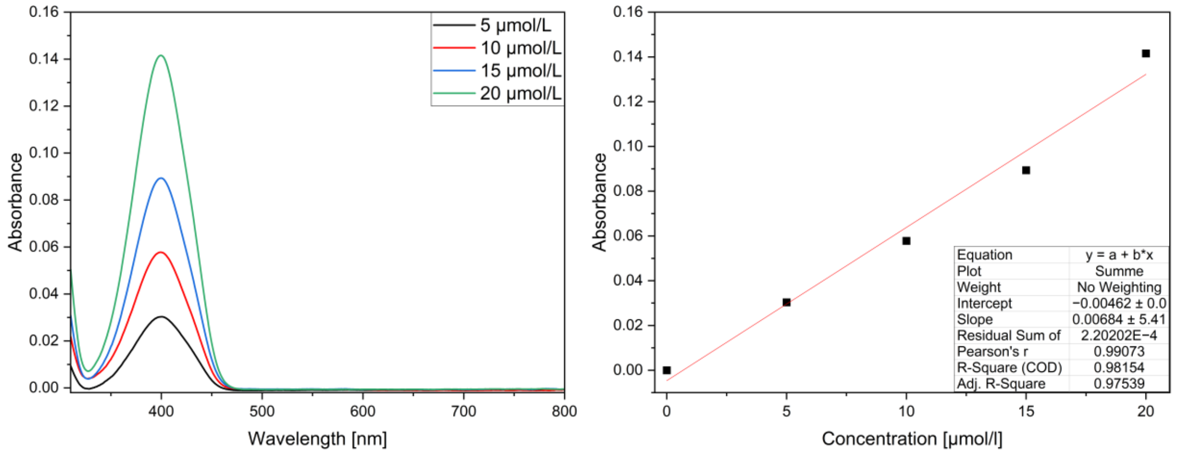

Figure S15: UV/Vis absorption spectra of $\mathbf{N_1O_3}$ in THF at different concentrations (left) and regression analysis (right).

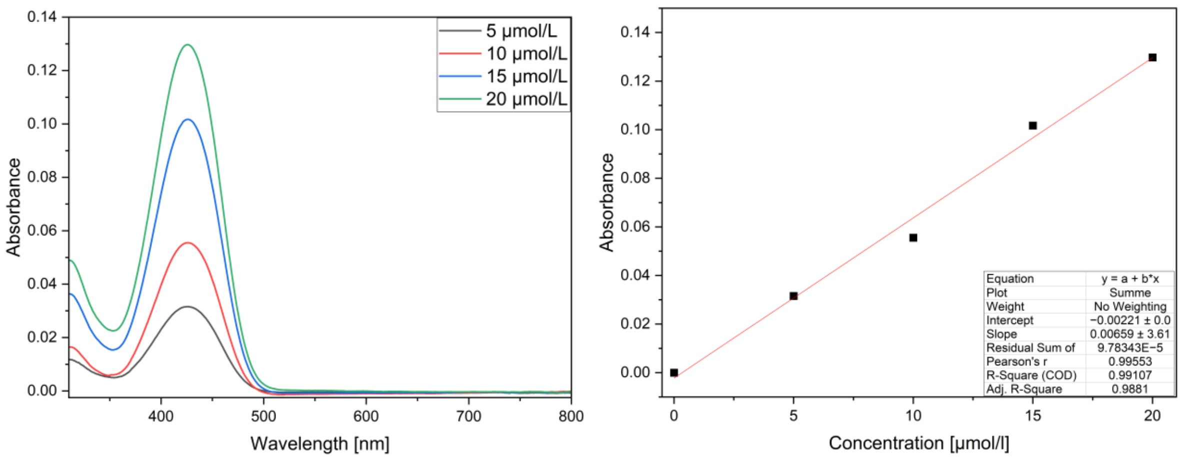

Figure S16: UV/Vis absorption spectra of $\mathbf{N_2O_2}$ in THF at different concentrations (left) and regression analysis (right).

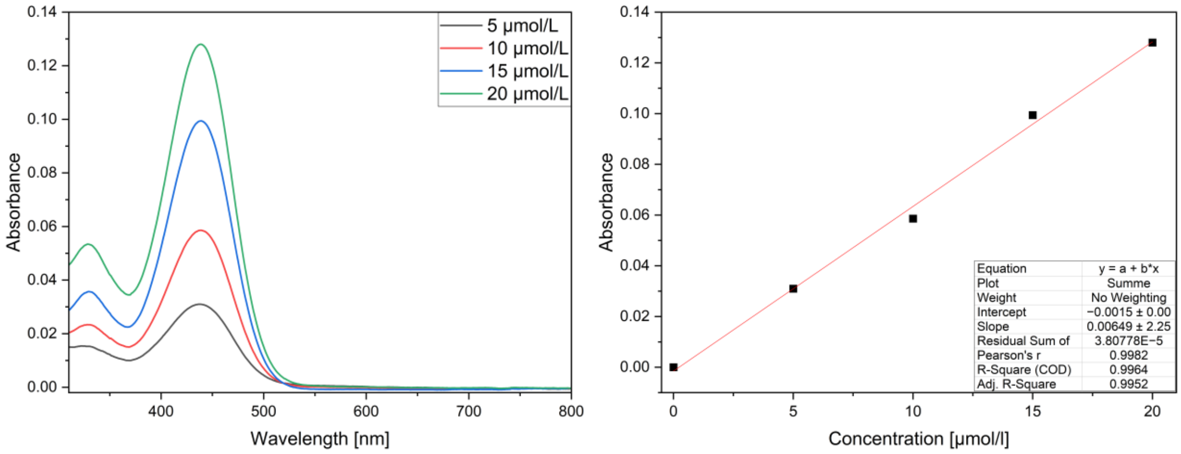

Figure S17: UV/Vis absorption spectra of $\mathbf{N_3O_1}$ in THF at different concentrations (left) and regression analysis (right).

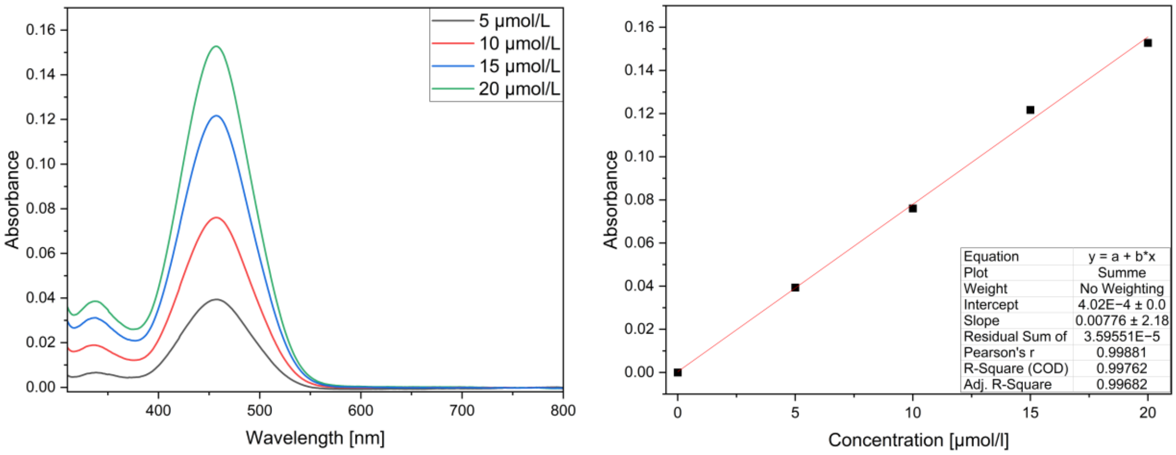

Figure S18: UV/Vis absorption spectra of $\mathbf{N_4}$ in THF at different concentrations (left) and regression analysis (right).

PHOTOLUMINESCENCE IN DMSO

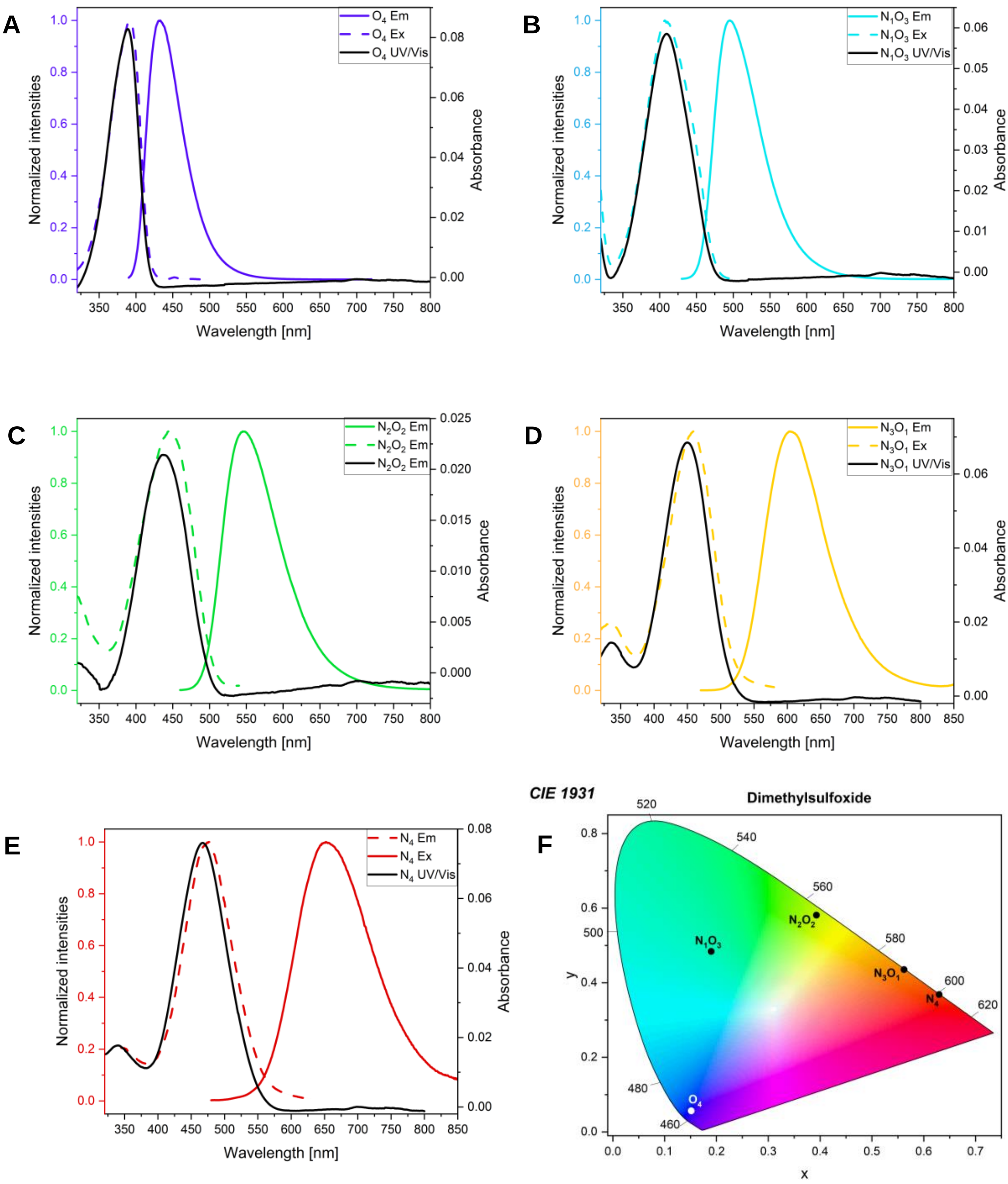

Figure S19: UV/Vis absorption (black solid line), normalized excitation (colored dotted line) and normalized emission (colored solid line) spectra (each 10 µM) for **A**) **O_4** (emission at λ_{ex} = 370 nm, excitation at λ_{em} = 520 nm); **B**) **N_1O_3** (emission at λ_{ex} = 410 nm, excitation at λ_{em} = 520 nm); **C**) **N_2O_2** (emission at λ_{ex} = 440 nm, excitation at λ_{em} = 560 nm); **D**) **N_3O_1** (emission at λ_{ex} = 450 nm, excitation at λ_{em} = 600 nm); **E**) **N_4** (emission at λ_{ex} = 470 nm, excitation at λ_{em} = 650 nm); CIE 1931 plot for all compounds in DMSO (**F**).

Table S1: Summary of the photophysical properties in DMSO (measured absorption ab and emission em wavelengths λ: Stokes shifts in nm ($\Delta\lambda$) and cm^{-1} (Δv), absolute photoluminescence quantum yields Φ_{PL}; brightness determination ($B = \Phi_{PL} \cdot \varepsilon$); amplitude-weighted average fluorescence lifetimes τ_{AvAmp} [ns] as well as average radiative and radiationless deactivation rate constants (k_r and k_{nr}). The photophysical parameters of **N_1O_3** and **N_2O_2** have been described in parts beforehand but were redetermined in this study to ensure completeness and instrumental comparability.

	**O_4**	**N_1O_3**	**N_2O_2**	**N_3O_1**	**N_4**
λ_{ab} [nm]	389	410	437	450	467
λ_{ex} [nm]	392	407	444	459	477
λ_{em} [nm]	433	495	545	603	652
Stokes shift $\Delta\lambda_{em\text{-}ab}$ [nm]	44	85	108	153	185
Stokes shift $\Delta v_{ab\text{-}em}$ [cm^{-1}]	2612	4188	4535	5638	6076
Φ_{PL}	0.23 ± 0.02	0.78 ± 0.03	0.69 ± 0.03	0.50 ± 0.03	0.14 ± 0.02
B [L·mol^{-1}·cm^{-1}]	1856	5335	4547	3245	1086
τ_{AvAmp} [ns]	3.237 ± 0.003	13.98 ± 0.03	13.38 ± 0.09	12.67 ± 0.02	4.057 ± 0.009
k_r [s^{-1}]	0.71 ± 0.06	0.56 ± 0.02	0.52 ± 0.02	0.39 ± 0.02	0.35 ± 0.05
k_{nr} [s^{-1}]	2.38 ± 0.06	0.16 ± 0.01	0.23 ± 0.02	0.39 ± 0.02	2.12 ± 0.05

SOLVATOCHROMISM

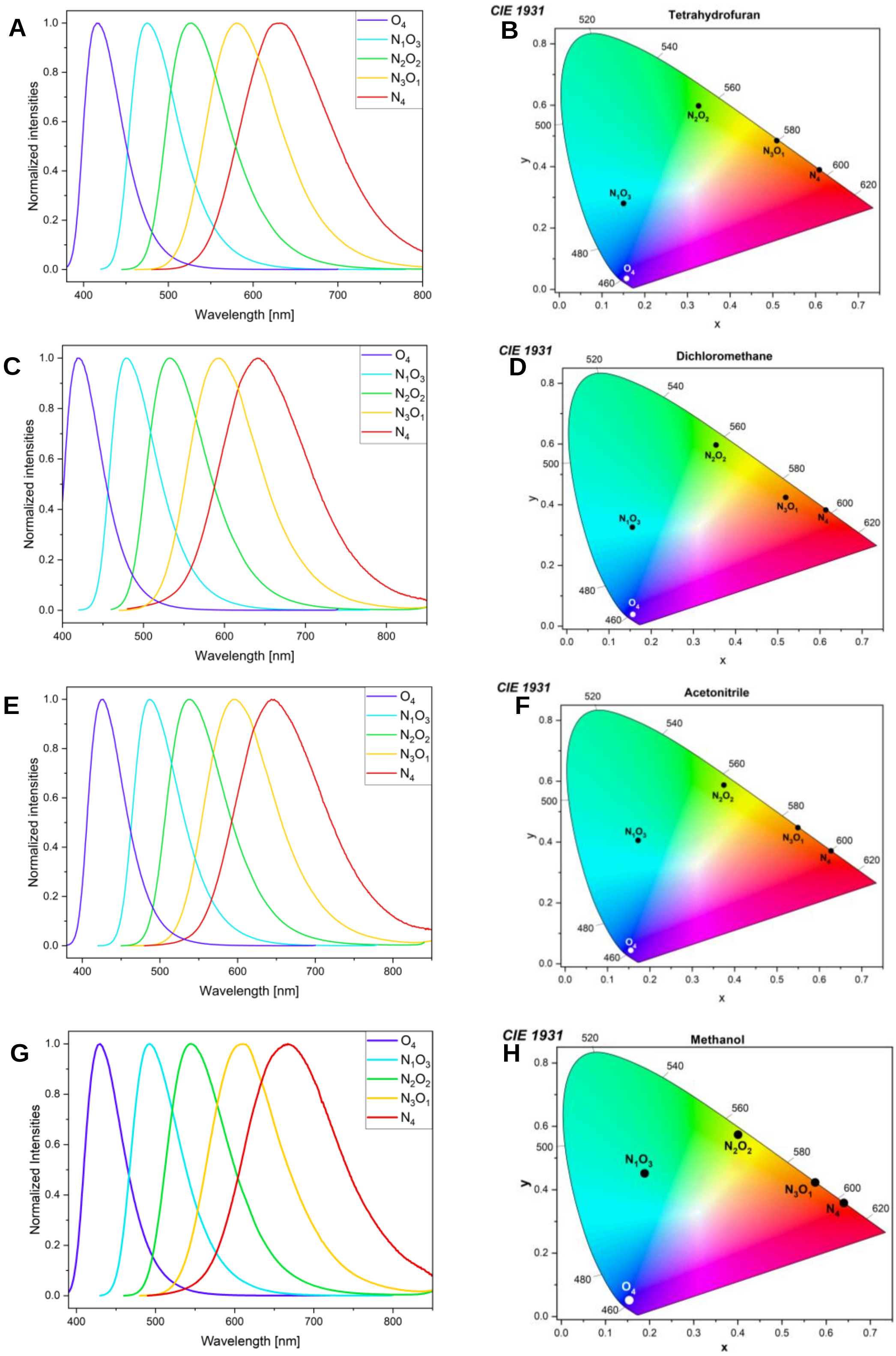

Figure S20: Normalized emission spectra of all compounds in **A**) THF, **C**) DCM, **E**) acetonitrile, and **G**) MeOH with corresponding CIE 1931 plots **B**), **D**), **F**), and **H**). $\mathbf{O_4}$ (emission at λ_{ex} = 370 nm, excitation at λ_{em} = 520 nm); **B**) $\mathbf{N_1O_3}$ (emission at λ_{ex} = 410 nm, excitation at λ_{em} = 520 nm); **C**) $\mathbf{N_2O_2}$ (emission at λ_{ex} = 440 nm, excitation at λ_{em} = 560 nm); **D**) $\mathbf{N_3O_1}$ (emission at λ_{ex} = 450 nm, excitation at λ_{em} = 600 nm); **E**) $\mathbf{N_4}$ (emission at λ_{ex} = 470 nm, excitation at λ_{em} = 650 nm).

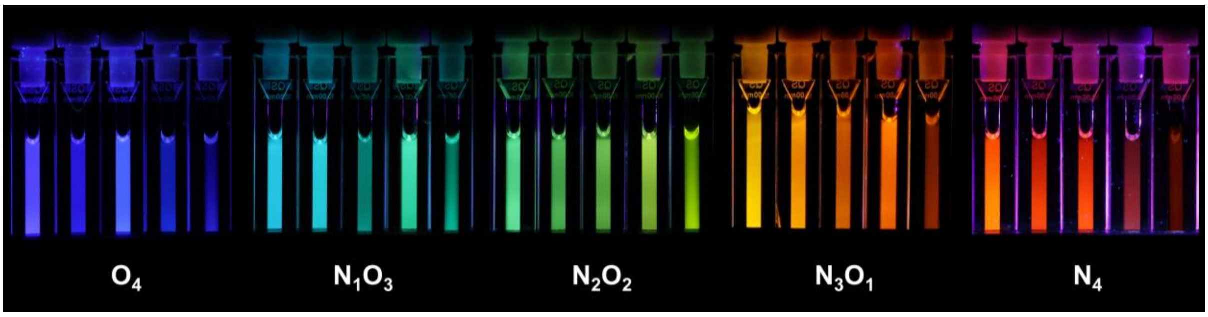

Figure S21: Pictures of all compounds in different solvents (f.l.t.r.: THF, DCM, ACN, DMSO, MeOH) under 395 nm UV light.

Table S2: Summary of the photophysical properties in THF, DCM, ACN, MeOH (measured and calculated absorption $_{ab}$ and emission $_{em}$ wavelengths λ, molar absorption coefficient ε (THF) and Stokes shifts in nm ($\Delta\lambda$) and cm^{-1} (Δv).

		O_4	N_1O_3	N_2O_2	N_3O_1	N_4
THF	ε [$l \cdot mol^{-1} \cdot cm^{-1}$]	8070	6840	6590	6490	7760
	λ_{ab} [nm]	380	400	426	439	457
	λ_{ex} [nm]	380	402	435	446	462
	λ_{em} [nm]	416	475	527	580	632
	$\Delta\lambda_{em\text{-}ab}$ [nm]	36	75	101	141	175
	$\Delta v_{ab\text{-}em}$ [cm^{-1}]	2277	3947	4499	5538	6059
DCM	λ_{ab} [nm]	382	403	435	445	462
	λ_{ex} [nm]	386	405	443	452	465
	λ_{em} [nm]	421	478	533	594	641
	$\Delta\lambda_{em\text{-}ab}$ [nm]	39	75	98	149	179
	$\Delta v_{ab\text{-}em}$ [cm^{-1}]	2312	3893	4227	5637	6044
ACN	λ_{ab} [nm]	383	404	431	443	461
	λ_{ex} [nm]	387	404	443	450	465
	λ_{em} [nm]	427	487	539	594	647
	$\Delta\lambda_{em\text{-}ab}$ [nm]	44	83	108	151	186
	$\Delta v_{ab\text{-}em}$ [cm^{-1}]	2690	4219	4703	5789	6236
MeOH	λ_{ex} [nm]	386	402	467	462	480
	λ_{em} [nm]	430	492	545	611	667

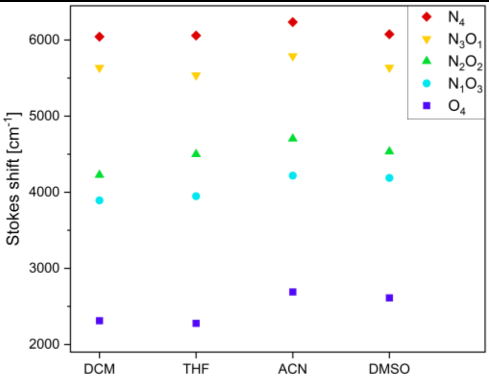

Figure S22: Overview of Stokes shifts [cm^{-1}] in each solvent.

PHOTOLUMINESCENCE IN POWDERS

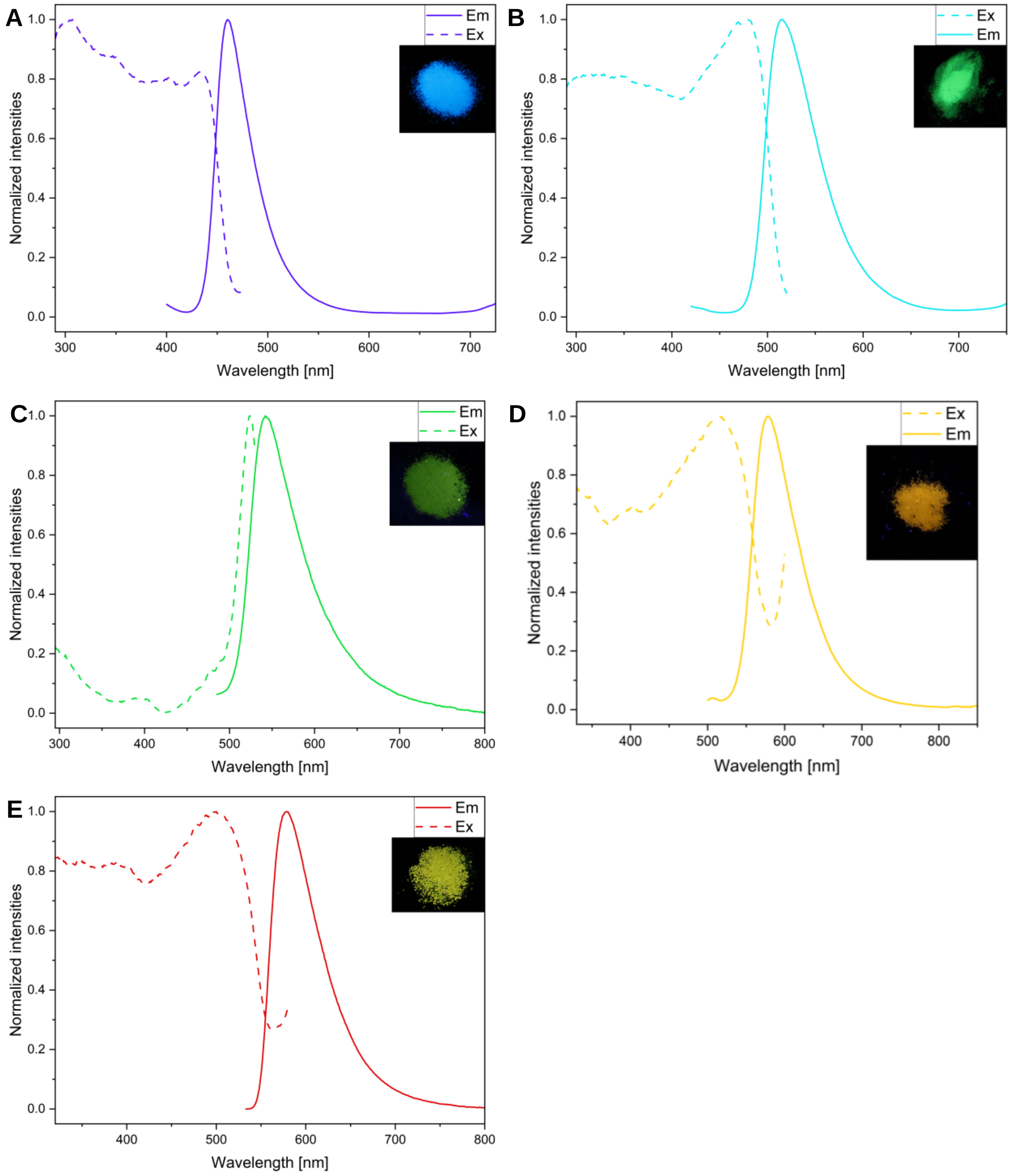

Figure S23: Excitation (dotted line) and emission (solid line) spectra for **A**) **O_4** (emission at λ_{ex} = 380 nm, excitation at λ_{em} = 500 nm); **B**) **N_1O_3** (emission at λ_{ex} = 400 nm, excitation at λ_{em} = 520 nm); **C**) **N_2O_2** (emission at λ_{ex} = 430 nm, excitation at λ_{em} = 550 nm); **D**) **N_3O_1** (emission at λ_{ex} = 480 nm, excitation at λ_{em} = 620 nm); **E**) **N_4** (emission at λ_{ex} = 460 nm, excitation at λ_{em} = 600 nm); in the solid-state as a powder with corresponding pictures taken under UV-light (395 nm).

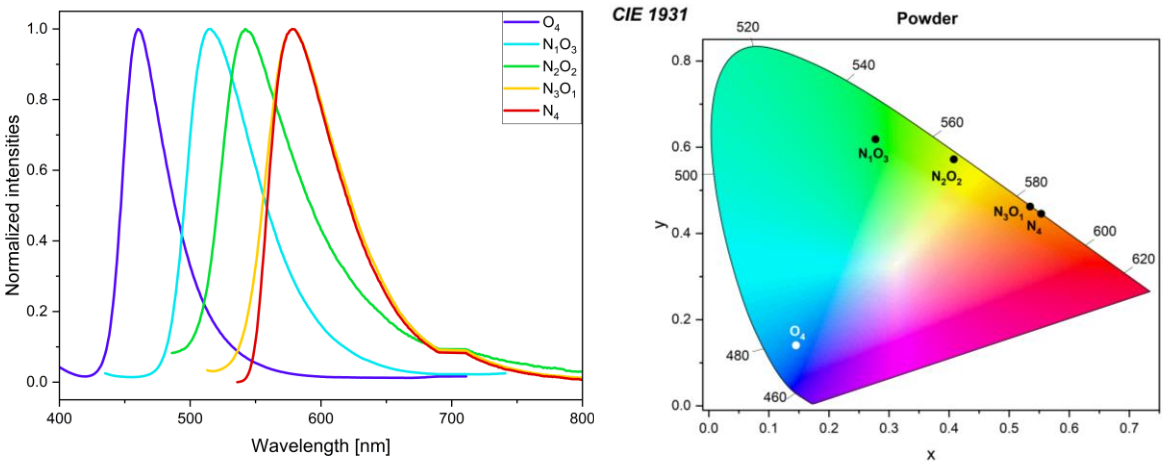

Figure S24: Normalized emission spectra of all compounds in the solid-state (powder, left) with the corresponding CIE 1931 plot (right).

Table S3: Summary of the photophysical properties in the solid-state (powder, measured excitation $_{ex}$ and emission $_{em}$ wavelengths λ: Stokes shifts in nm ($\Delta\lambda$) and cm^{-1} ($\Delta\nu$), absolute photoluminescence quantum yields Φ_{PL}; amplitude-weighted average fluorescence lifetimes τ_{AvAmp} [ns] as well as average radiative and radiationless deactivation rate constants (k_r and k_{nr}); * = calculated for excitation wavelengths since no absorption wavelengths were determined).

	O_4	N_1O_3	N_2O_2	N_3O_1	N_4
λ_{ex} [nm]	434	479	524	513	500
λ_{em} [nm]	460	515	542	578	578
$\Delta\lambda_{em-ex}$ [nm]*	26	36	18	65	78
$\Delta\nu_{ex-em}$ [cm^{-1}]*	1302	1459	634	2192	2699
Φ_{PL}	0.13 ± 0.02	0.32 ± 0.02	0.01 ± 0.02	0.03 ± 0.02	<0.01 ± 0.02
τ_{AvAmp} [ns]	4.26 ± 0.05	12.78 ± 0.04	5.20 ± 0.29	1.19 ± 0.05	0.480 ± 0.002
k_r [s^{-1}]	0.31 ± 0.05	0.25 ± 0.02	0.02 ± 0.04	0.25 ± 0.18	0.21 ± 0.42
k_{nr} [s^{-1}]	2.04 ± 0.06	0.53 ± 0.02	1.90 ± 0.09	8.15 ± 0.33	20.64 ± 0.45

PHOTOLUMINESCENCE IN PMMA FILMS

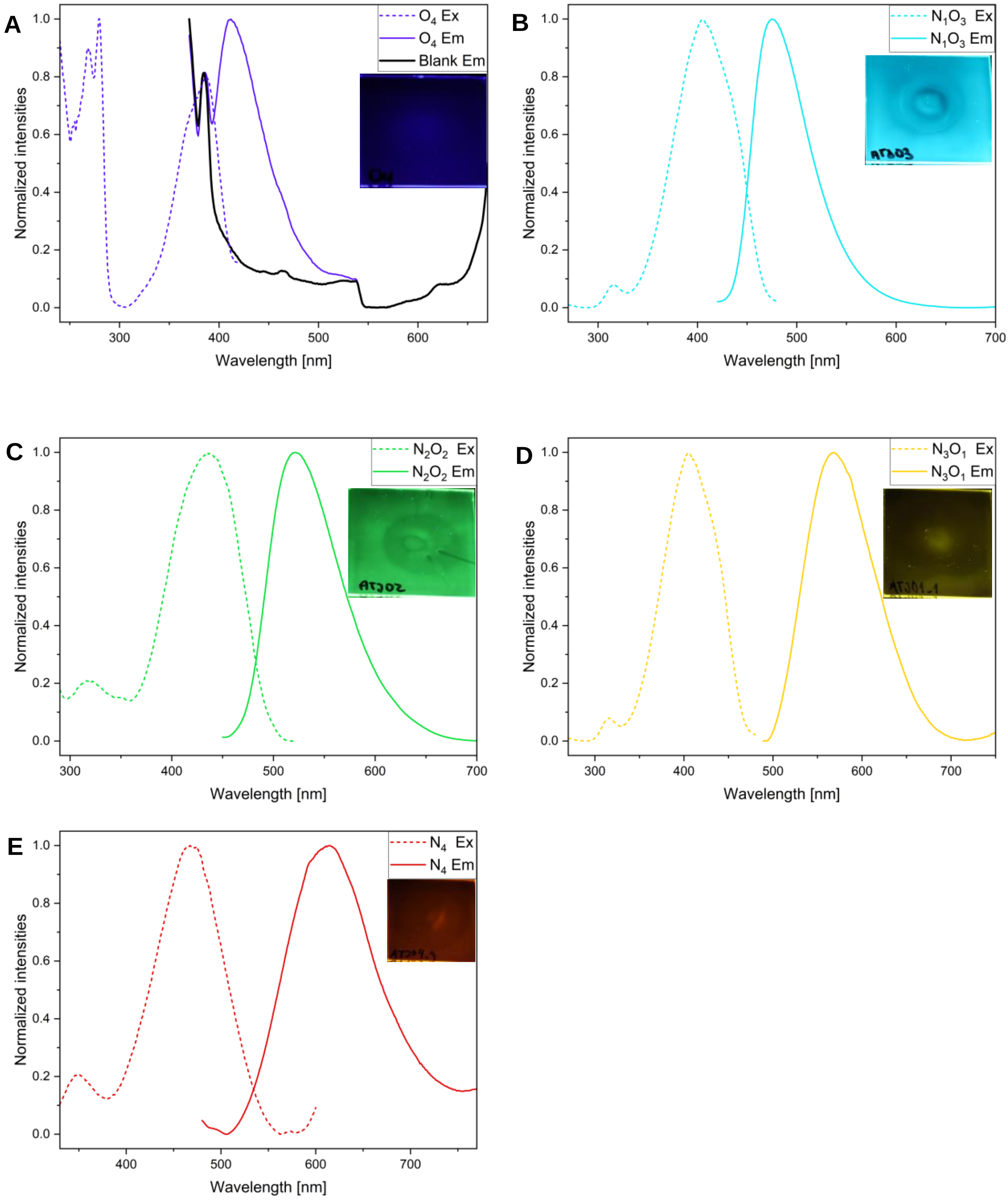

Figure S25: Excitation (dotted line) and emission (solid line) spectra for **A)** $\mathbf{O_4}$ (emission at λ_{ex} = 350 nm, excitation at λ_{em} = 440 nm); **B)** $\mathbf{N_1O_3}$ (emission at λ_{ex} = 400 nm, excitation at λ_{em} = 500 nm); **C)** $\mathbf{N_2O_2}$ (emission at λ_{ex} = 430 nm, excitation at λ_{em} = 540 nm); **D)** $\mathbf{N_3O_1}$ (emission at λ_{ex} = 460 nm, excitation at λ_{em} = 580 nm); **E)** $\mathbf{N_4}$ (emission at λ_{ex} = 450 nm, excitation at λ_{em} = 620 nm); in PMMA films with corresponding pictures taken under UV-light (395 nm); **A)** is showing the emission spectrum of the blank material without added dye, where an emission maximum at 390 nm is detected.

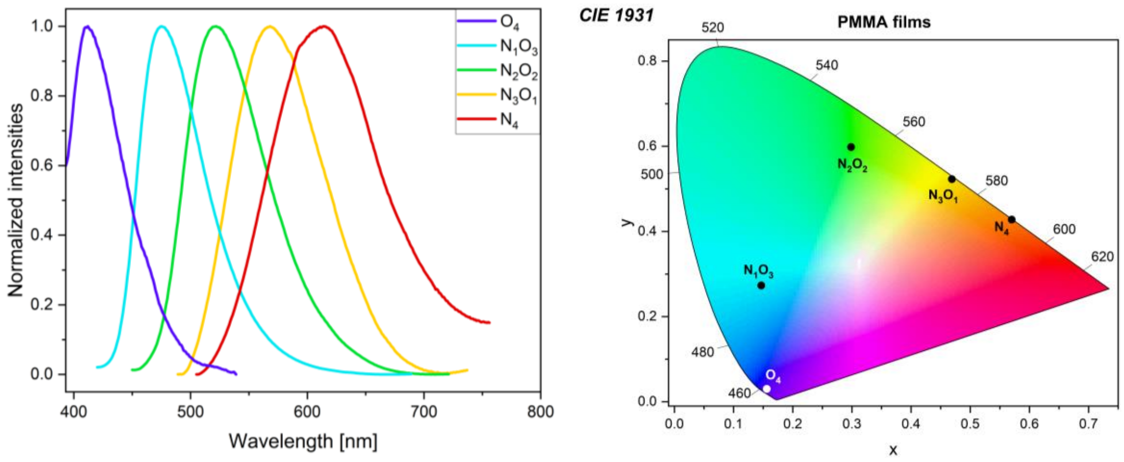

Figure S26: Normalized emission spectra of all compounds in the PMMA films (left) with the corresponding CIE 1931 plot (right).

Table S4: Summary of the photophysical properties in PMMA films (measured excitation ex and emission em wavelengths λ: Stokes shifts in nm ($\Delta\lambda$) and cm^{-1} (Δv), absolute photoluminescence quantum yields Φ_{PL}; amplitude-weighted average fluorescence lifetimes τ_{AvAmp} [ns] as well as average radiative and radiationless deactivation rate constants (k_r and k_{nr}); * = calculated for excitation wavelengths since no absorption wavelengths were determined).

	O_4	N_1O_3	N_2O_2	N_3O_1	N_4
λ_{ex} [nm]	387	405	435	453	467
λ_{em} [nm]	410	480	510	560	605
$\Delta\lambda_{em-ex}$ [nm]*	23	75	75	107	138
Δv_{ex-em} [cm^{-1}]*	1450	3858	3381	4218	4884
Φ_{PL}	0.19 ± 0.02	0.61 ± 0.03	0.76 ± 0.03	0.65 ± 0.03	0.49 ± 0.02
τ_{AvAmp} [ns]	2.10 ± 0.03	10.51 ± 0.30	12.93 ± 0.19	12.82 ± 0.17	12.28 ± 0.20
k_r [s^{-1}]	0.90 ± 0.11	0.58 ± 0.04	0.59 ± 0.02	0.51 ± 0.02	0.40 ± 0.02
k_{nr} [s^{-1}]	3.86 ± 0.12	0.37 ± 0.02	0.19 ± 0.02	0.27 ± 0.02	0.42 ± 0.02

PHOTOLUMINESCENCE IN 3D-PRINTED HEXAGONS

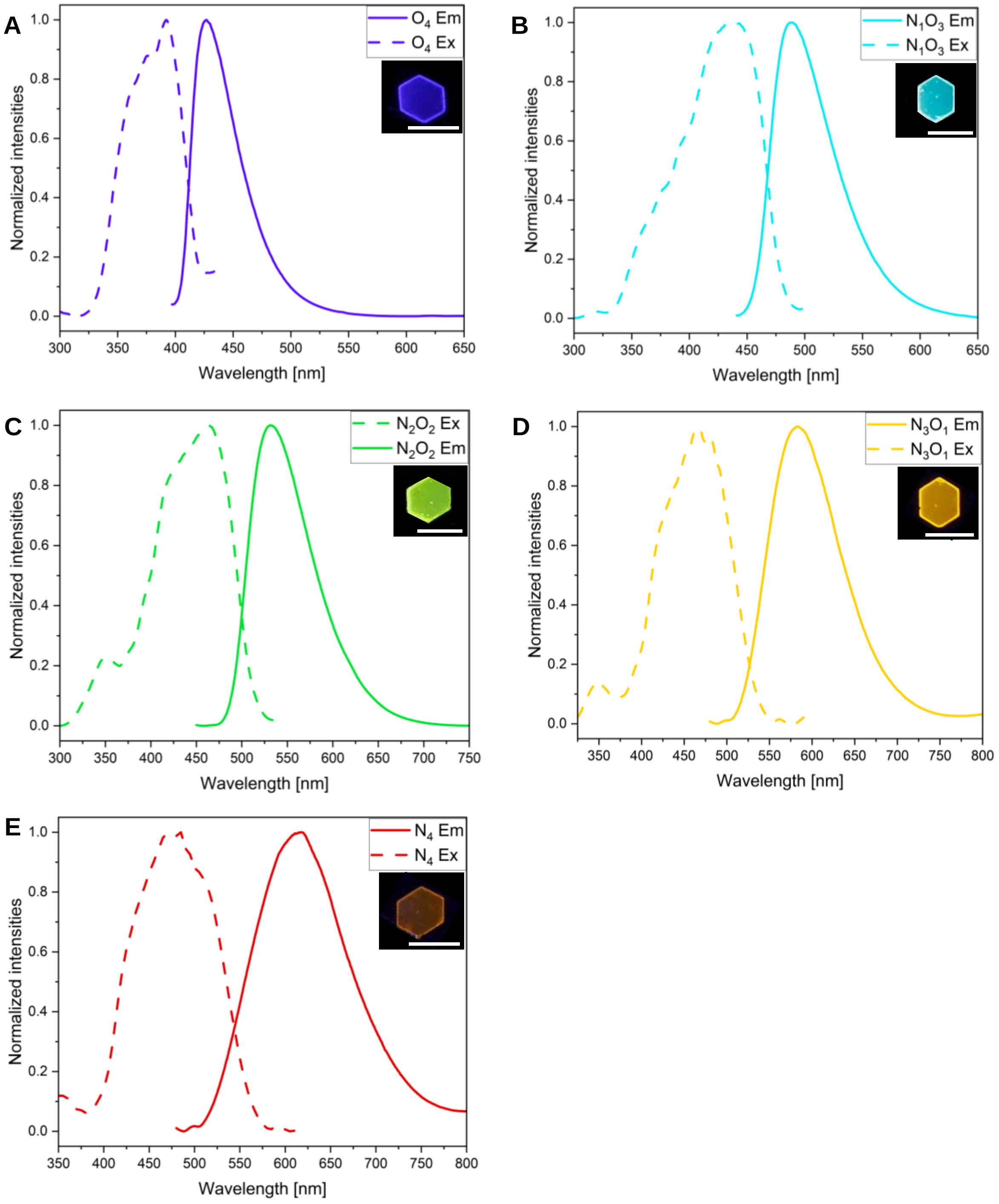

Figure S27: Excitation (dotted line) and emission (solid line) spectra for **A**) **O_4** (emission at λ_{ex} = 380 nm, excitation at λ_{em} = 460 nm); **B**) **N_1O_3** (emission at λ_{ex} = 400 nm, excitation at λ_{em} = 520 nm); **C**) **N_2O_2** (emission at λ_{ex} = 430 nm, excitation at λ_{em} = 560 nm); **D**) **N_3O_1** (emission at λ_{ex} = 460 nm, excitation at λ_{em} = 610 nm); **E**) **N_4** (emission at λ_{ex} = 460 nm, excitation at λ_{em} = 640 nm); in 3D-printed hexagons with corresponding pictures taken under UV-light (395 nm). Scale bar: 4 mm.

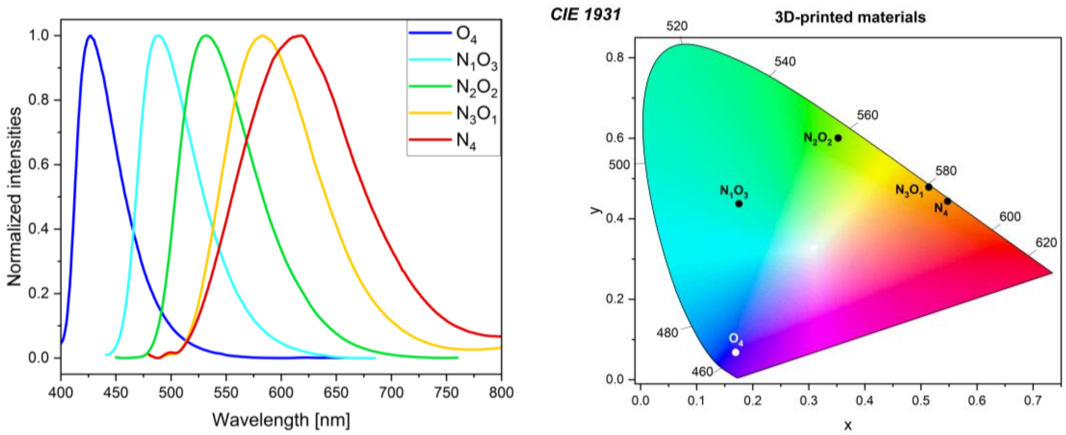

Figure S28: Normalized emission spectra of all compounds in the 3D-printed hexagons (left) with the corresponding CIE 1931 plot (right).

Table S5: Summary of the photophysical properties in 3D-printed hexagons (measured excitation $_{ex}$ and emission $_{em}$ wavelengths λ: Stokes shifts in nm ($\Delta\lambda$) and cm^{-1} (Δv), absolute photoluminescence quantum yields Φ_{PL}; amplitude-weighted average fluorescence lifetimes τ_{AvAmp} [ns] as well as average radiative and radiationless deactivation rate constants (k_r and k_{nr}); * = calculated for excitation wavelengths since no absorption wavelengths were determined).

	O_4	N_1O_3	N_2O_2	N_3O_1	N_4
λ_{ex} [nm]	392	435	462	467	485
λ_{em} [nm]	427	489	531	582	617
$\Delta\lambda_{em\text{-}ex}$ [nm]*	35	54	69	115	132
$\Delta v_{ex\text{-}em}$ [cm^{-1}]*	2091	2539	2813	4231	4411
Φ_{PL}	0.09 ± 0.02	0.67 ± 0.03	0.70 ± 0.03	0.48 ± 0.02	0.26 ± 0.02
τ_{AvAmp} [ns]	2.592 ± 0.006	13.39 ± 0.01	14.61 ± 0.04	13.13 ± 0.07	9.93 ± 0.04
k_r [s^{-1}]	0.35 ± 0.08	0.50 ± 0.02	0.48 ± 0.02	0.37 ± 0.02	0.26 ± 0.02
k_{nr} [s^{-1}]	3.51 ± 0.08	0.25 ± 0.02	0.21 ± 0.01	0.40 ± 0.02	0.75 ± 0.02

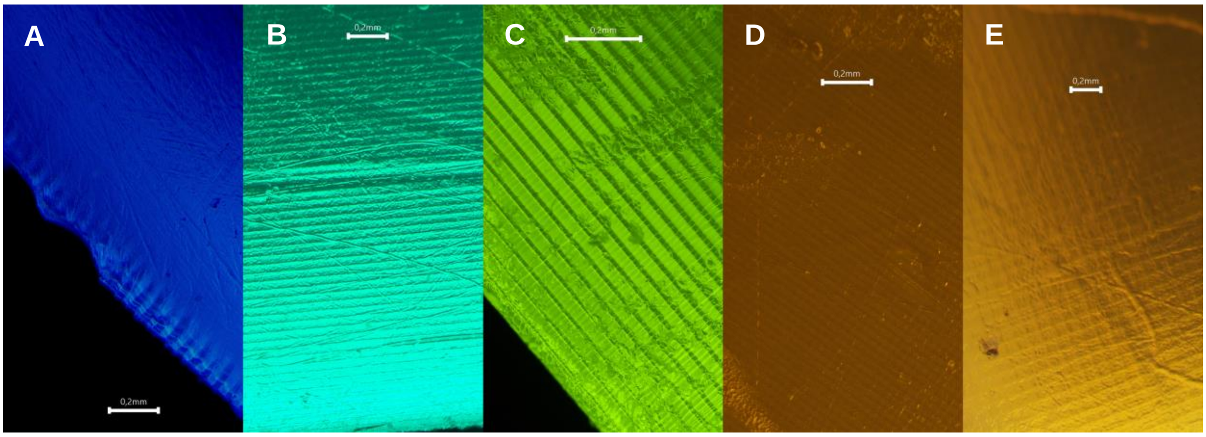

Figure S29: Microscopic pictures of the 3D-printed objects under UV-light (365 nm), showing full homogeneity (**A**) **O_4**; **B**) **N_1O_3**; **C**) **N_2O_2**; **D**) **N_3O_1**; **E**) **N_4**); scale bar: 0.2 mm each.

PHOTOLUMINESCENCE IN PLURONIC® F-127 NANOPARTICLES

Pluronic® F-127 nanoparticles with incorporated dyes were prepared according to a slightly modified protocol of Gallavardin *et al*.[14] For this, 50 mg of Pluronic® F-127 was dissolved in water (5 mL, 1 wt%) and 2 mg of dye was dissolved in DCM (100 µL). Both solutions were combined, and the resulting suspension stirred for 15 minutes, before sonicating for 15 minutes (150 W). After evaporating residual DCM *via* rotovap (50 °C, 850 mbar, 30 minutes), the dispersion was filtrated using a nylon-based syringe filter (200 nm pore size), yielding the corresponding aqueous nanoparticle solution (100 mg/L).

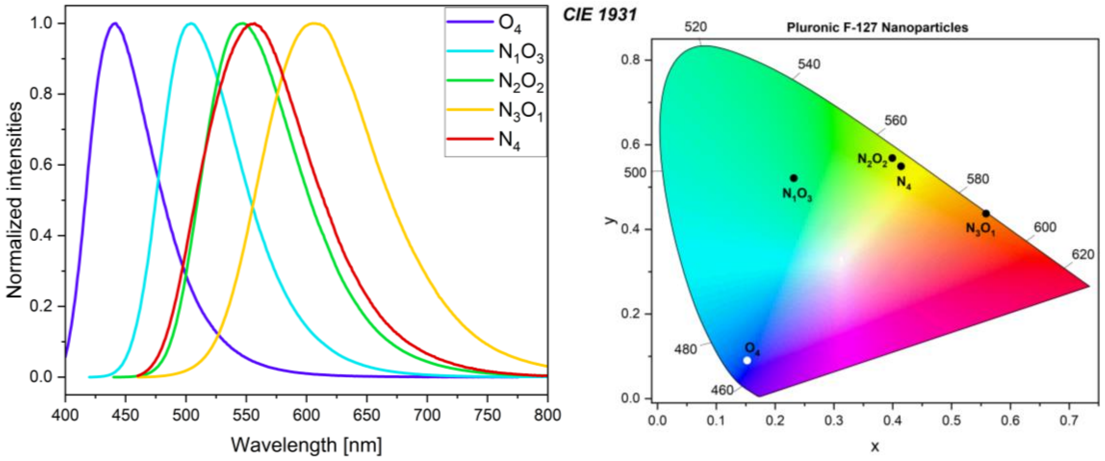

Figure S30: Normalized emission spectra of all compounds in Pluronic® F-127 nanoparticles (left) with the corresponding CIE 1931 plot (right). **O_4** (emission at λ_{ex} = 350 nm); **B**) **N_1O_3** (emission at λ_{ex} = 400 nm); **C**) **N_2O_2** (emission at λ_{ex} = 410 nm); **D**) **N_3O_1** (emission at λ_{ex} = 440 nm); **E**) **N_4** (emission at λ_{ex} = 440 nm);

Table S6: Summary of the photophysical properties in Pluronic® F-127 nanoparticles (measured excitation $_{ex}$ and emission $_{em}$ wavelengths λ: Stokes shifts in nm ($\Delta\lambda$) and cm^{-1} ($\Delta\nu$); * = calculated for excitation wavelengths since no absorption wavelengths were determined).

	O_4	N_1O_3	N_2O_2	N_3O_1	N_4
λ_{ex} [nm]	388	399	443	458	458
λ_{em} [nm]	442	504	547	605	557
$\Delta\lambda_{em-ex}$ [nm]*	54	105	104	147	99
$\Delta\nu_{ex-em}$ [cm^{-1}]*	3149	5221	4292	5305	3881

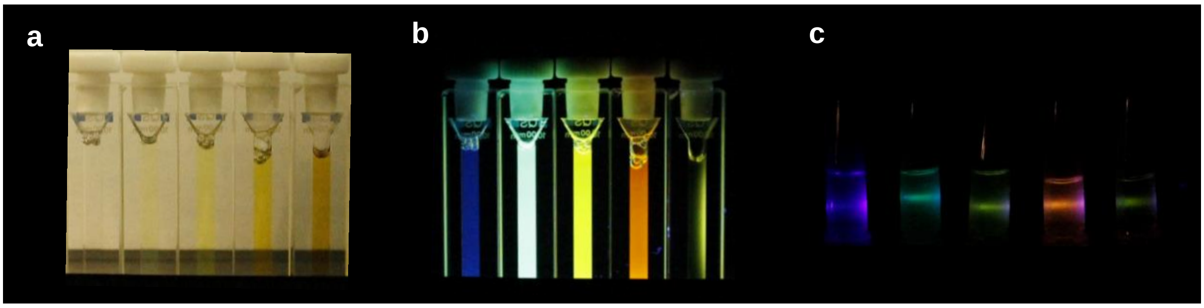

Figure S31: Pictures of the compounds in Pluronic® F-127 nanoparticles taken under daylight (**a**), under UV light (365 nm, **b**) and upon irradiation with a 405 nm laser (**c**).

AGGREGATION SERIES

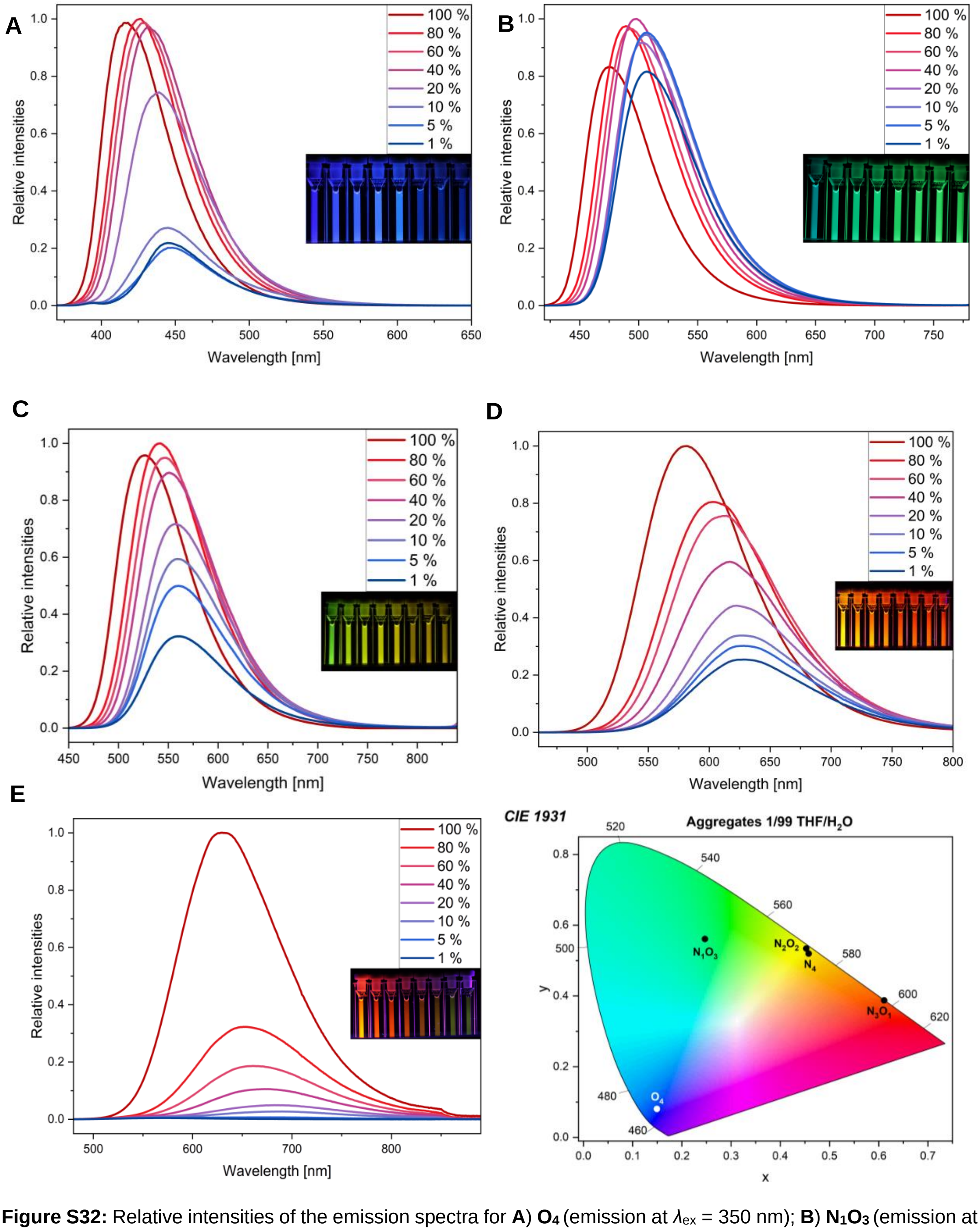

Figure S32: Relative intensities of the emission spectra for **A**) **O_4** (emission at λ_{ex} = 350 nm); **B**) **N_1O_3** (emission at λ_{ex} = 400 nm); **C**) **N_2O_2** (emission at λ_{ex} = 430 nm); **D**) **N_3O_1** (emission at λ_{ex} = 440 nm); **E**) **N_4** (emission at λ_{ex} = 460 nm) in mixtures of THF/H_2O from 100% THF (red) to 99% H_2O (blue line) with corresponding pictures taken under UV-light (395 nm); **F**) CIE 1931 plot for the aggregates at THF/ H_2O 1/99.

Table S7: Relative intensities of the compounds in mixtures of THF and H_2O (highest respective intensity normalized to 1).

% THF	O_4	N_1O_3	N_2O_2	N_3O_1	N_4
**100**	0.987	0.833	0.958	1.000	1.000
**80**	1.000	0.975	1.000	0.805	0.323
**60**	0.986	0.966	0.949	0.756	0.186
**40**	0.969	1.000	0.897	0.596	0.106
**20**	0.744	0.917	0.717	0.443	0.049
**10**	0.272	0.944	0.594	0.339	0.027
**5**	0.202	0.953	0.500	0.302	0.007
**1**	0.219	0.816	0.323	0.253	0.005

Table S8: Calculated Stokes shifts in cm^{-1} for the mixtures of the compounds in THF and H_2O (* = no data due to low values).

% THF	O_4	N_1O_3	N_2O_2	N_3O_1	N_4
**100**	2393	3947	4499	5597	6009
**80**	2842	4550	5024	6195	6568
**60**	3060	4716	8042	6492	6730
**40**	3221	4920	5325	6572	7067
**20**	3537	5159	5521	6702	7219
**10**	3844	5276	5585	6805	7389
**5**	3995	5276	5617	6855	7133
**1**	4044	5276	5680	6855	n.d.*

ACIDOCHROMISM

General method: Stock solutions of the compounds were prepared at a concentration of 1 mM in 1 mL of DCM and then diluted to 15 µM in 1000 µL DCM. 116 µL of TFA were dissolved in 11.6 mL DCM (129 mM) for the acid stock solution. From this solution, 11.6 µL were added to the compound solution, corresponding to 100 eq. of acid. 104 µL of triethylamine (TEA) were dissolved in 396 µL DCM (1.5 M) for the base stock solution. From this solution, 5 µL were added to the compound solution afterwards, corresponding to 500 eq. of base.

For $\mathbf{N_4}$, the appearance of a blue-shifted emission band was observed. Hence, the protonation in the presence of TFA was measured after 18 h, revealing a new green emission band with a maximum at 540 nm, which is intensified upon neutralization. Hence, NMR spectroscopy was used to investigate the resulting mixture, which indicates acid-promoted degradation to unidentified products.

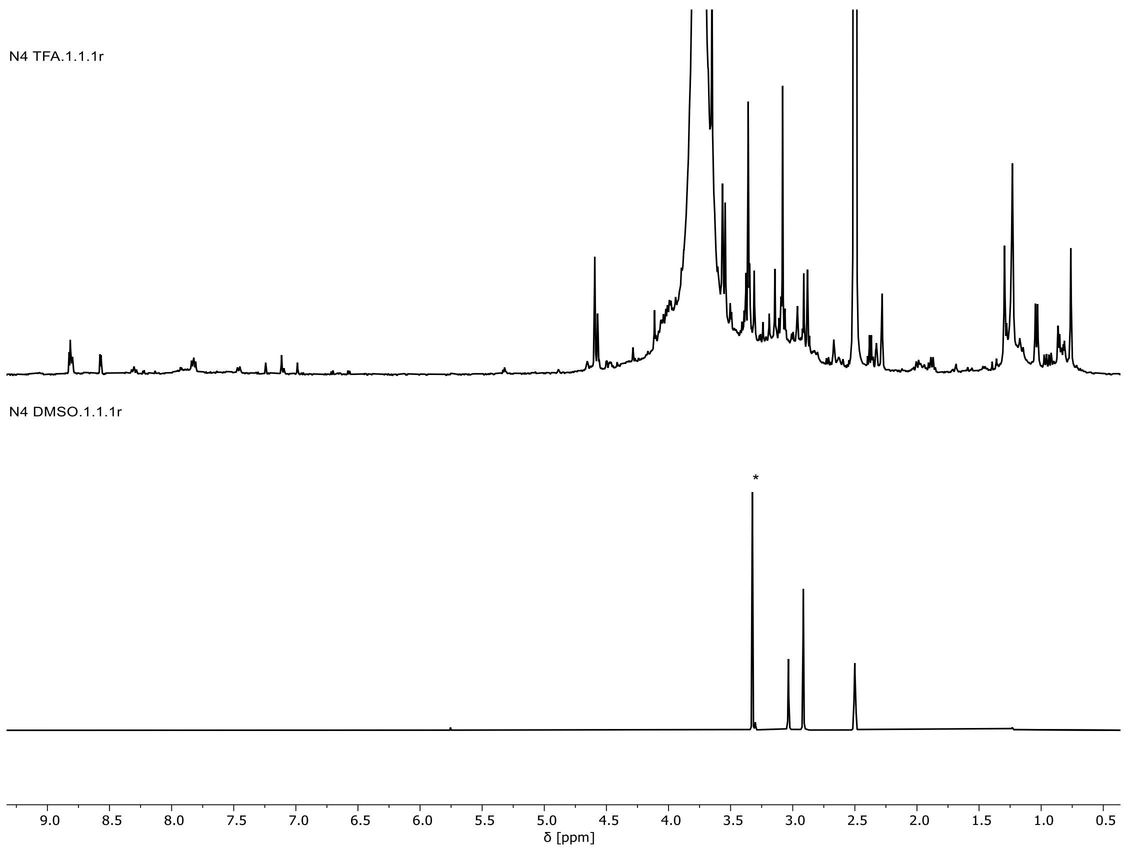

Figure S33: ^{1}H-NMR spectra (400 MHz, DMSO-d_6, 298 K) of compound $\mathbf{N_4}$ (* = water). Bottom: before addition of TFA; top: after addition of 100 eq. TFA and subsequent concentration *in vacuo*.

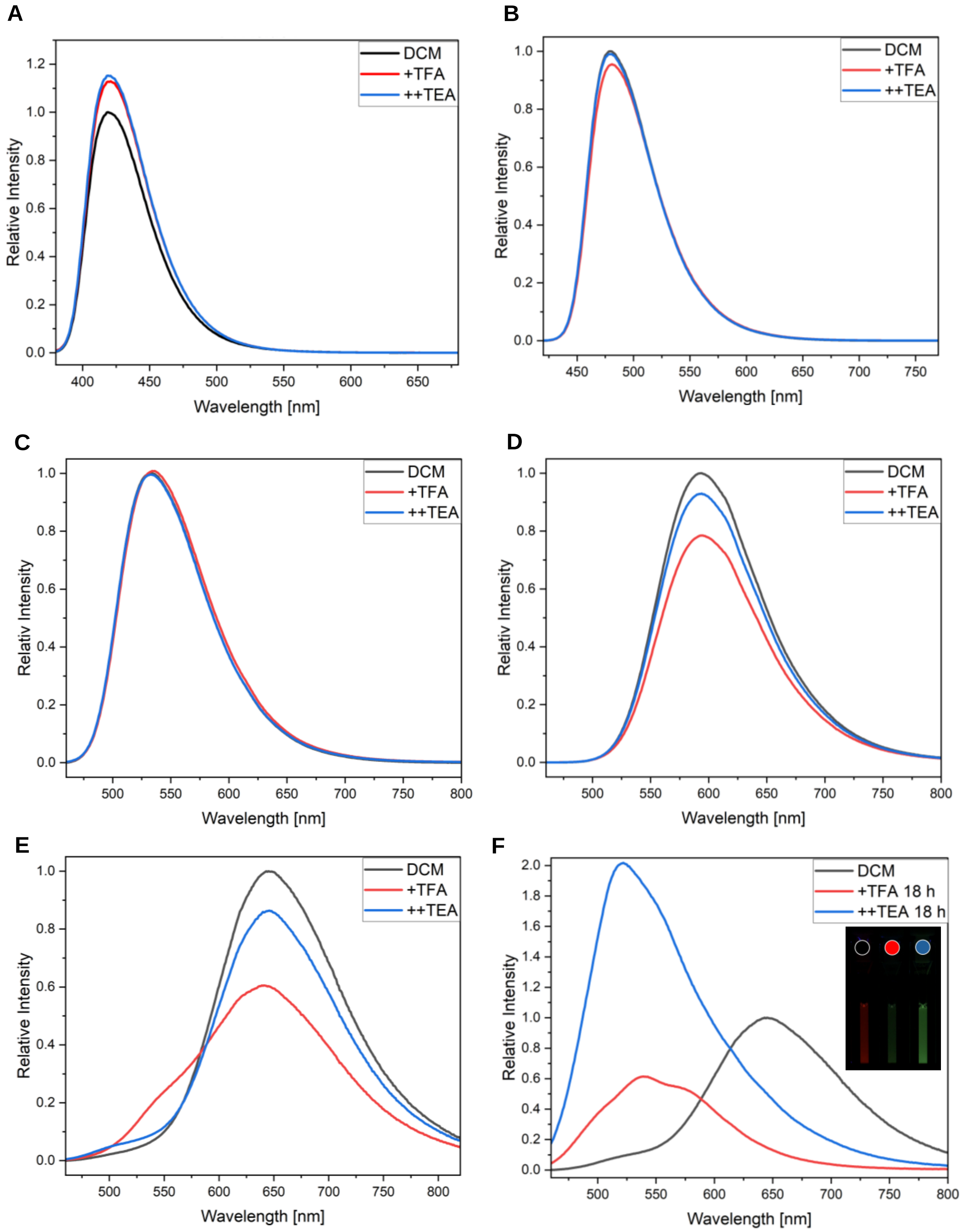

Figure S34: Relative intensities of emission spectra of **A**) **O_4** (emission at λ_{ex} = 360 nm); **B**) **N_1O_3** (emission at λ_{ex} = 400 nm); **C**) **N_2O_2** (emission at λ_{ex} = 440 nm); **D**) **N_3O_1** (emission at λ_{ex} = 440 nm); **E**) **N_4** (emission at λ_{ex} = 440 nm) and **F**) **N_4** (emission after 18 h storage in acid at λ_{ex} = 440 nm). Compounds in DCM (15 µM, black line), after addition of 100 eq. TFA (red line) and after addition of 500 eq. TEA (blue line) with corresponding pictures taken under UV light (365 nm).

White Light Emission experiments

In DCM

For the White-Light Emission (WLE) experiments, a mixture of compounds **O_4**, **N_2O_2** and **N_3O_1** was chosen because one approach to achieve WLE is by combining blue and yellow emission. Thus, 100 µM solutions in DCM were prepared and excited with 365 nm. This allowed to determine the relative emission intensities of **O_4** (1.75x) and **N_2O_2** (2.6x) compared to **N_3O_1**. Considering that **N_2O_2** and **N_3O_1** yield yellow emission, 0.385 eq. of **N_2O_2** and 1.0 eq. **N_3O_1** were mixed. This solution was combined with 1.142 eq. of **O_4** as the blue component to achieve WLE in DCM.

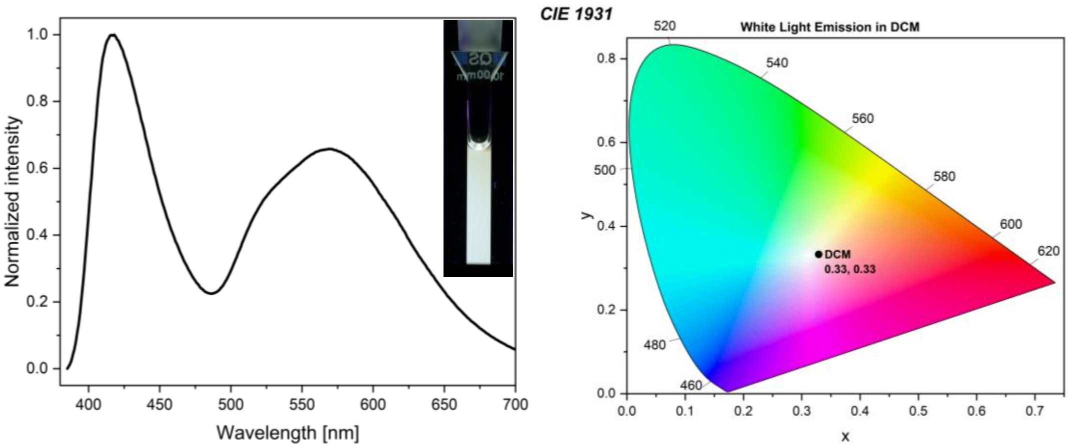

Figure S35: Normalized emission intensity (left, λ_{ex} = 365 nm) of the WLE mixture consisting of 1.142 eq. **O_4**, 0.385 eq. **N_2O_2** and 1.0 eq. **N_3O_1** prepared from 100 µM solutions in DCM with a photograph taken under 365 nm UV light; corresponding CIE 1931 plot (right).

Upon irradiation, we observed a photodegradation for the WLE experiments in DCM. Thus, a freshly prepared sample was irradiated with 365 nm and measured after certain time intervals.

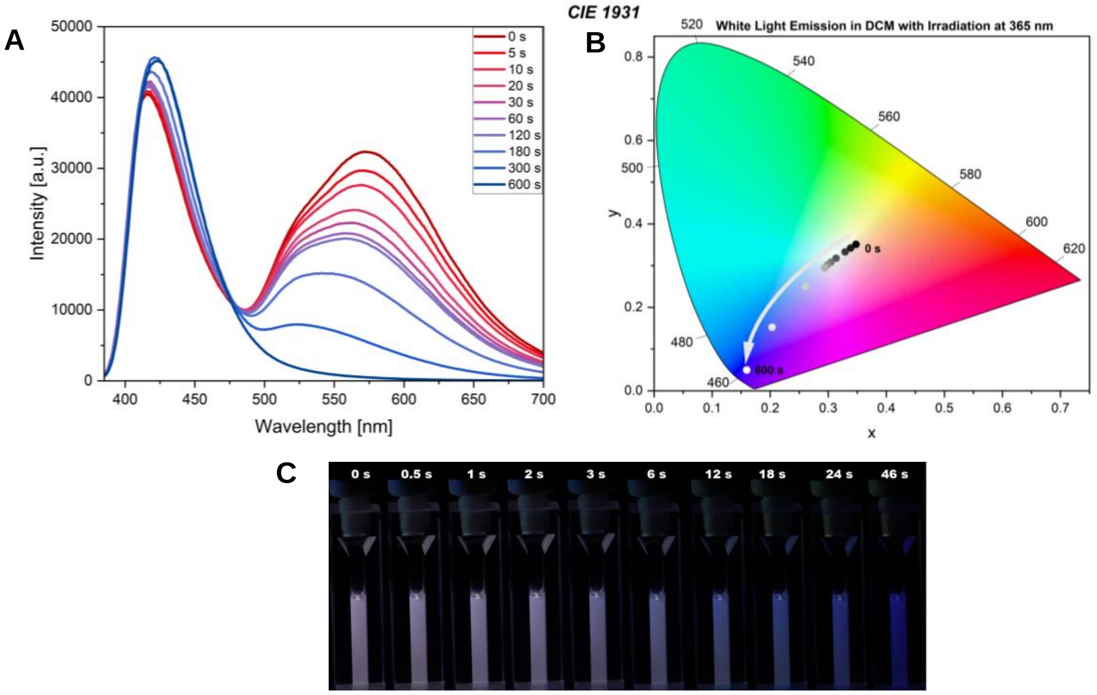

Figure S36: A) Observed photodegradation for the WLE experiment upon irradiation with 365 nm in the fluorescence spectrometer (λ_{ex} = 365 nm); B) corresponding CIE plot; C) pictures taken upon irradiation with a LZ1-10UV0R-0000 LED by *OSRAM*® with 700 mA power source.

IN 3D-PRINTED HEXAGON

To achieve WLE in a 3D-printed hexagon, we initially chose the same ratios as for the DCM solutions to obtain a total of 0.1 wt% in 1 g of resin. However, the final 3D-printed hexagon showed yellow emission. Hence, we increased the blue component to a total of 2.284 eq. of $\mathbf{O_4}$, 0.385 eq. of $\mathbf{N_2O_2}$ and 1.0 eq. $\mathbf{N_3O_1}$ to yield the 3D-printed hexagon showed in Figure S37.

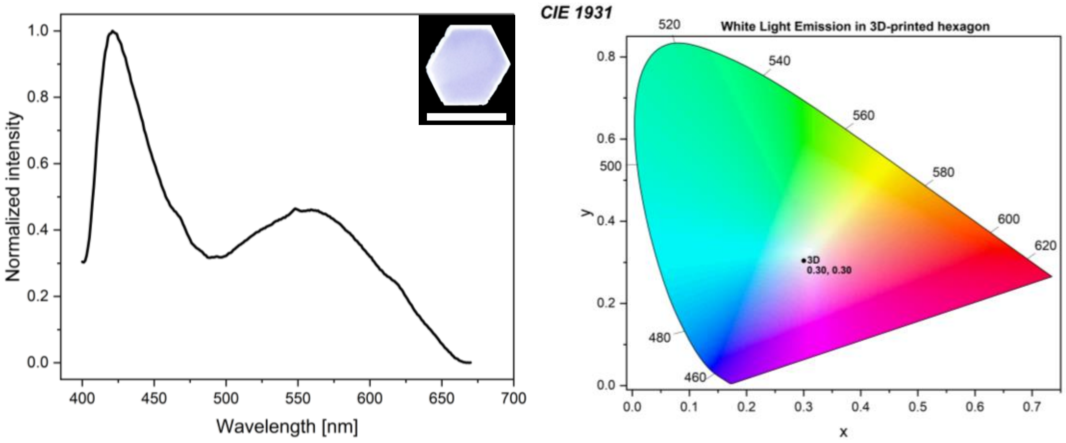

Figure S37: Normalized emission intensity (left, λ_{ex} = 365 nm) of the 3D-printed hexagon consisting of 2.284 eq. $\mathbf{O_4}$, 0.385 eq. $\mathbf{N_2O_2}$ and 1.0 eq. $\mathbf{N_3O_1}$ with a photograph taken under 365 nm UV light; corresponding CIE 1931 plot (right). Scalebar: 4 mm.

Then, the photostability in the 3D-printed hexagon was tested by irradiating with a 365 nm LED (LZ1-10UV0R-0000 LED by *OSRAM*®) with 700 mA power source for 600 s (Figure S38).

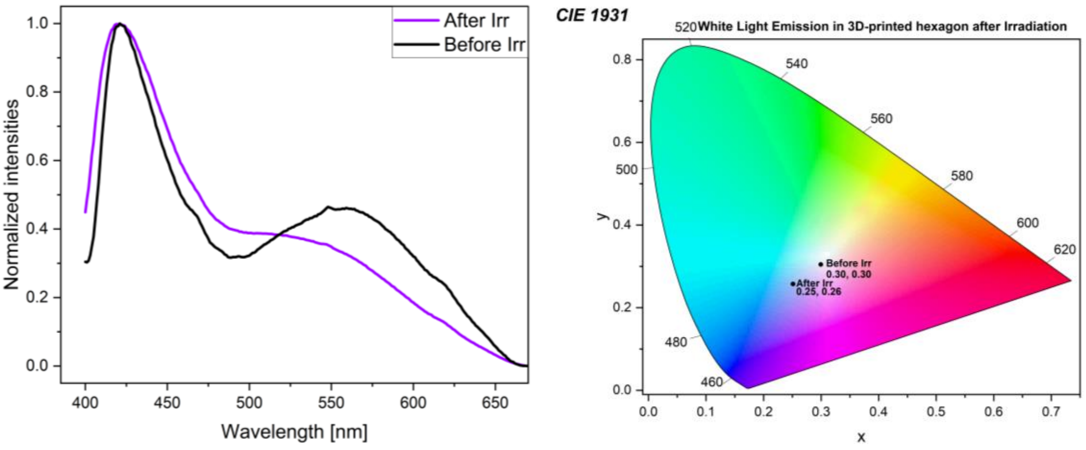

Figure S38: Comparison of normalized emission intensities (left, λ_{ex} = 365 nm) of the 3D-printed hexagon before (black line) and after (purple line) 600 s of irradiation with a 365 nm LED; corresponding CIE 1931 plot (right).

SUMMARY OF THE PHOTOPHYSICAL PROPERTIES

Table S9: Summary of the photophysical properties.

Property	Medium	O_4	N_1O_3	N_2O_2	N_3O_1	N_4
λ_{ab} / λ_{ex}^{*} [nm]	DMSO	389	410	437	450	467
	PMMA*	387*	405*	435*	453*	467*
	3D-object*	392*	435*	462*	467*	485*
	Powder*	434*	479*	524*	513*	500*
λ_{em} [nm]	DMSO	433	495	545	603	652
	PMMA	410	480	510	560	605
	3D-object	427	489	531	582	617
	Powder	460	515	542	578	578
Φ_{PL}	DMSO	0.23 ± 0.02	0.78 ± 0.04	0.69 ± 0.03	0.50 ± 0.03	0.14 ± 0.02
	PMMA	0.19 ± 0.02	0.61 ± 0.03	0.76 ± 0.04	0.65 ± 0.03	0.49 ± 0.03
	3D-object	0.09 ± 0.02	0.67 ± 0.03	0.70 ± 0.03	0.48 ± 0.03	0.26 ± 0.02
	Powder	0.13 ± 0.02	0.32 ± 0.02	0.01 ± 0.02	0.03 ± 0.02	<0.01 ± 0.02
τ_{AvAmp} [ns]	DMSO	3.237 ± 0.003	13.98 ± 0.03	13.38 ± 0.09	12.67 ± 0.02	4.057 ± 0.009
	PMMA	2.10 ± 0.03	10.51 ± 0.30	12.93 ± 0.19	12.82 ± 0.17	12.28 ± 0.20
	3D-object	2.592 ± 0.006	13.39 ± 0.01	14.61 ± 0.04	13.13 ± 0.07	9.93 ± 0.04
	Powder	4.26 ± 0.05	12.78 ± 0.04	5.20 ± 0.29	1.19 ± 0.05	0.480 ± 0.002

CIE Coordinates

Table S10: Coordinates (x, y values) of the CIE plots in all investigated media.

Medium	O_4	N_1O_3	N_2O_2	N_3O_1	N_4
THF	(0.15, 0.04)	(0.15, 0.28)	(0.33, 0.60)	(0.51, 0.49)	(0.61, 0.39)
DCM	(0.16, 0.04)	(0.16, 0.33)	(0.35, 0.60)	(0.52, 0.42)	(0.61, 0.38)
ACN	(0.15, 0.04)	(0.17, 0.41)	(0.37, 0.59)	(0.55, 0.45)	(0.63, 0.37)
DMSO	(0.15, 0.06)	(0.19, 0.48)	(0.39, 0.58)	(0.56, 0.44)	(0.63, 0.37)
Powder	(0.14, 0.14)	(0.27, 0.62)	(0.41, 0.57)	(0.53, 0.46)	(0.55, 0.45)
3D-objects	(0.17, 0.07)	(0.18, 0.44)	(0.35, 0.60)	(0.51, 0.48)	(0.55, 0.44)
PMMA films	(0.16, 0.03)	(0.15, 0.27)	(0.30, 0.60)	(0.47, 0.52)	(0.57, 0.43)
Pluronic® F-127	(0.15, 0.09)	(0.23, 0.52)	(0.40, 0.57)	(0.56, 0.44)	(0.41, 0.55)
Aggregates	(0.15, 0.08)	(0.25, 0.56)	(0.45, 0.53)	(0.61, 0.39)	(0.46, 0.52)

LIFETIME REPORTS

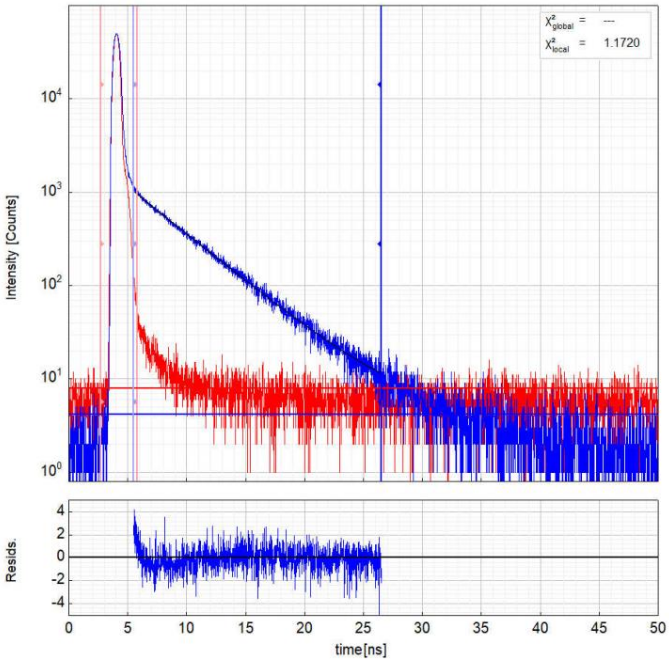

Parameter	Value	Δ	δ
A_1[kCnts/Chnl]	1.434	±0.026	1.8%
τ_1[ns]	4.257	±0.054	1.3%
I_1[kCnts]	381.5	±2.0	0.5%
A_{Rel1}[%]	100.0	---	---
I_{Rel1}[%]	100.0	---	---
$Bkgr_{Dec}$[kCnts]	0.0042	±0.0008	18%
$Bkgr_{IRF}$[Cnts/Chnl]	8.0000	---	---
$Shift_{IRF}$[ps]	0.0000	---	---
τ_{AvInt}[ns]	4.257	±0.054	1.3%
τ_{AvAmp}[ns]	4.257	±0.054	1.3%

Figure S39: Raw time-resolved photoluminescence decay of **O4** in the solid-state (blue) with instrumental response function in red (left), including the residuals (λ_{ex} = 440.0 nm, λ_{em} = 460 nm); fitting parameters including pre-exponential factors and confidence limits (right).

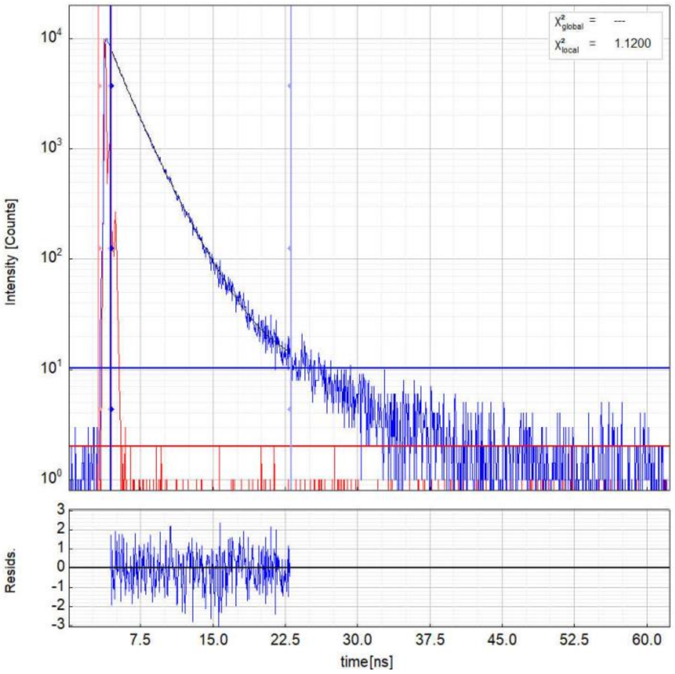

Parameter	Value	Δ	δ
A_1[kCnts/Chnl]	5.2	±1.5	28%
τ_1[ns]	2.71	±0.15	5.4%
I_1[kCnts]	218	±47	21%
A_{Rel1}[%]	47	±13	27%
I_{Rel1}[%]	60	±13	21%
A_2[kCnts/Chnl]	6.0	±1.4	23%
τ_2[ns]	1.58	±0.19	12%
I_2[kCnts]	148	±46	31%
A_{Rel2}[%]	54	±13	23%
I_{Rel2}[%]	41	±13	31%
$Bkgr_{Dec}$[kCnts]	0.0103	±0.0015	14%
$Bkgr_{IRF}$[Cnts/Chnl]	2.0000	---	---
$Shift_{IRF}$[ps]	0.0000	---	---
τ_{AvInt}[ns]	2.247	±0.016	0.7%
τ_{AvAmp}[ns]	2.097	±0.030	1.4%

Figure S40: Raw time-resolved photoluminescence decay of **O4** in the PMMA film (blue) with instrumental response function in red (left), including the residuals (λ_{ex} = 373.0 nm, λ_{em} = 410 nm); fitting parameters including pre-exponential factors and confidence limits (right).

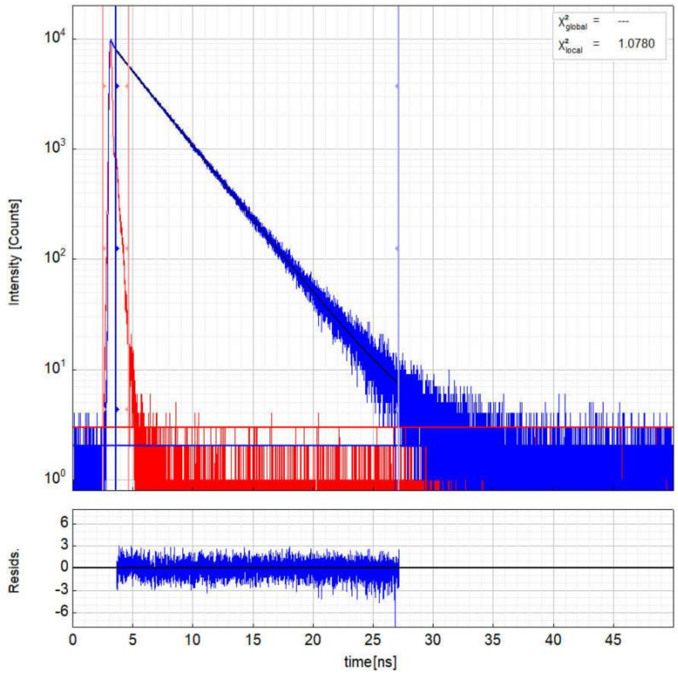

Parameter	Value	Δ	δ
A_1[kCnts/Chnl]	8.688	±0.012	0.1%
τ_1[ns]	3.2369	±0.0032	0.1%
I_1[kCnts]	7 030.3	±4.9	0.1%
A_{Rel1}[%]	100.0	---	---
I_{Rel1}[%]	100.0	---	---
$Bkgr_{Dec}$[kCnts]	0.0021	±0.0003	10%
$Bkgr_{IRF}$[Cnts/Chnl]	3.0000	---	---
$Shift_{IRF}$[ps]	10.0000	---	---
A_{Scatt}[kCnts]	792	±34	4.3%
τ_{AvInt}[ns]	3.2369	±0.0032	0.1%
τ_{AvAmp}[ns]	3.2369	±0.0032	0.1%

Figure S41: Raw time-resolved photoluminescence decay of **O4** in DMSO (10 µM) with instrumental response function in red (left), including the residuals (λ_{ex} = 373.0 nm, λ_{em} = 433 nm); fitting parameters including pre-exponential factors and confidence limits (right).

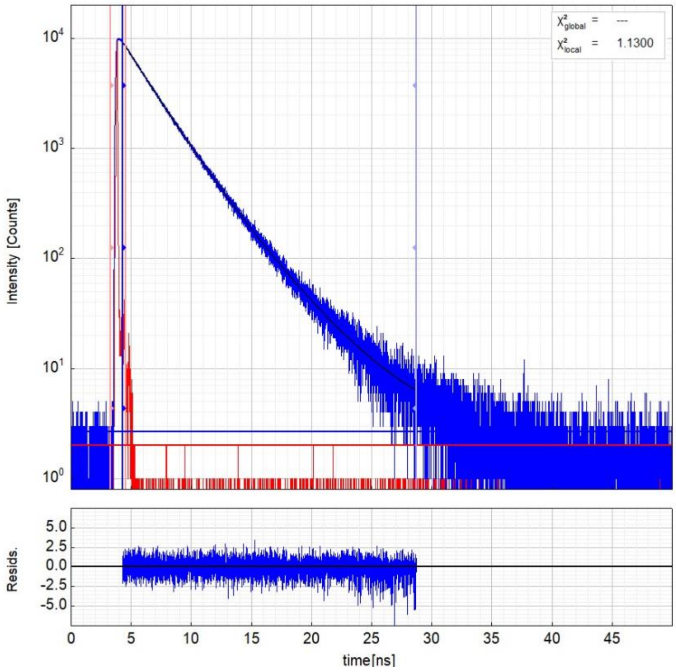

Parameter	Value	Δ	δ
A_1[kCnts/Chnl]	1.51	±0.53	35%
τ_1[ns]	4.09	±0.26	6.2%
I_1[kCnts]	1 540	±410	26%
A_{Rel1}[%]	13.4	±4.7	34%
I_{Rel1}[%]	21.2	±5.6	26%
A_2[kCnts/Chnl]	9.74	±0.51	5.1%
τ_2[ns]	2.361	±0.049	2.0%
I_2[kCnts]	5 750	±410	7.1%
A_{Rel2}[%]	86.7	±4.7	5.3%
I_{Rel2}[%]	78.9	±5.6	7.1%
$Bkgr_{Dec}$[kCnts]	0.0027	±0.0006	22%
$Bkgr_{IRF}$[Cnts/Chnl]	2.0000	---	---
$Shift_{IRF}$[ps]	0.0000	---	---
τ_{AvInt}[ns]	2.7254	±0.0068	0.2%
τ_{AvAmp}[ns]	2.5918	±0.0055	0.2%

Figure S42: Raw time-resolved photoluminescence decay of **O4** in the 3D-printed hexagon (0.1 wt%) with instrumental response function in red (left), including the residuals (λ_{ex} = 373.0 nm, λ_{em} = 427 nm); fitting parameters including pre-exponential factors and confidence limits (right).

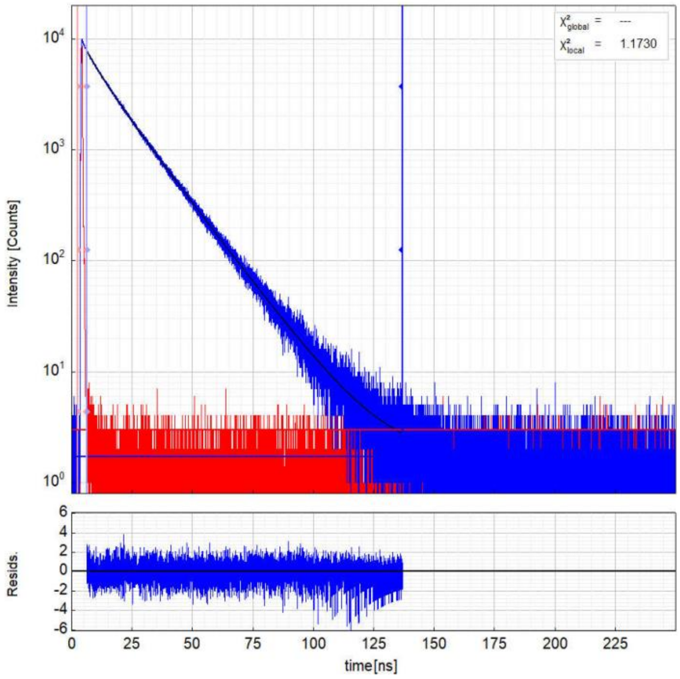

Parameter	Value	Δ	δ
A_1[kCnts/Chnl]	2.532	±0.066	2.6%
$τ_1$[ns]	6.37	±0.25	3.8%
I_1[kCnts]	1 007	±64	6.3%
A_{Rel1}[%]	27.1	±0.8	2.8%
I_{Rel1}[%]	13.5	±0.9	6.4%
A_2[kCnts/Chnl]	6.827	±0.094	1.4%
$τ_2$[ns]	15.163	±0.054	0.4%
I_2[kCnts]	6 470	±67	1.0%
A_{Rel2}[%]	73.0	±0.8	1.0%
I_{Rel2}[%]	86.6	±0.9	1.0%
$Bkgr_{Dec}$[kCnts]	0.0018	±0.0001	3.8%
$Bkgr_{IRF}$[Cnts/Chnl]	3.0000	---	---
$Shift_{IRF}$[ps]	150.0000	---	---
$τ_{AvInt}$[ns]	13.978	±0.014	0.1%
$τ_{AvAmp}$[ns]	12.783	±0.041	0.3%

Figure S43: Raw time-resolved photoluminescence decay of **N_1O_3** in the solid-state (blue) with instrumental response function in red (left), including the residuals ($λ_{ex}$ = 440.0 nm, $λ_{em}$ = 515 nm); fitting parameters including pre-exponential factors and confidence limits (right).

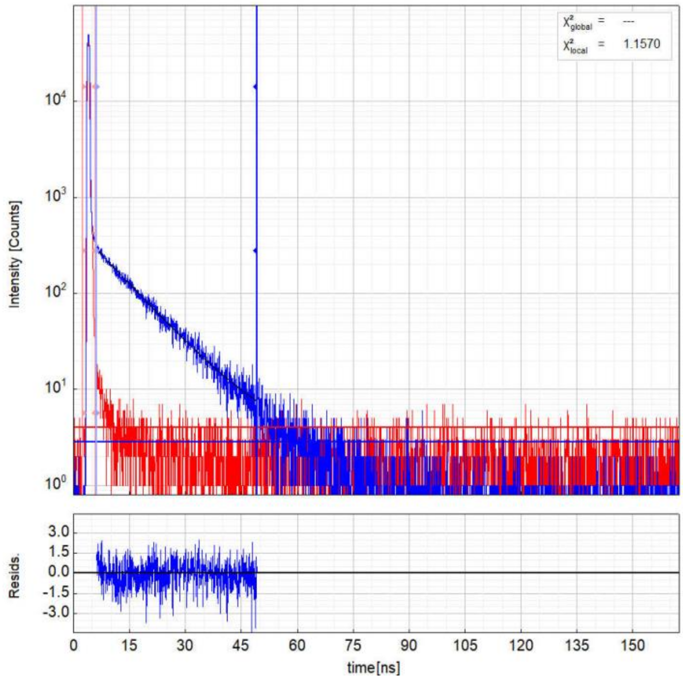

Parameter	Value	Δ	δ
A_1[kCnts/Chnl]	0.3528	±0.0073	2.0%
$τ_1$[ns]	10.51	±0.30	2.8%
I_1[kCnts]	57.91	±0.90	1.6%
A_{Rel1}[%]	100.0	---	---
I_{Rel1}[%]	100.0	---	---
$Bkgr_{Dec}$[kCnts]	0.0029	±0.0015	52%
$Bkgr_{IRF}$[Cnts/Chnl]	4.0000	---	---
$Shift_{IRF}$[ps]	50.0000	---	---
$τ_{AvInt}$[ns]	10.51	±0.30	2.8%
$τ_{AvAmp}$[ns]	10.51	±0.30	2.8%

Figure S44: Raw time-resolved photoluminescence decay of **N_1O_3** in the PMMA film (blue) with instrumental response function in red (left), including the residuals ($λ_{ex}$ = 440.0 nm, $λ_{em}$ = 480 nm); fitting parameters including pre-exponential factors and confidence limits (right).

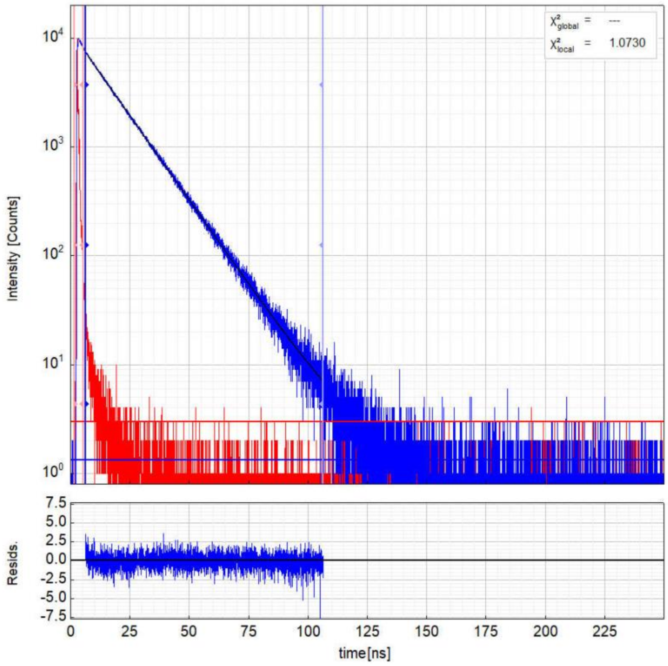

Parameter	Value	Δ	δ
A_1[kCnts/Chnl]	7.406	±0.020	0.3%
τ_1[ns]	13.979	±0.028	0.2%
I_1[kCnts]	3 234.9	±6.5	0.2%
A_{Rel1}[%]	100.0	---	---
I_{Rel1}[%]	100.0	---	---
$Bkgr_{Dec}$[kCnts]	0.0014	±0.0003	18%
$Bkgr_{IRF}$[Cnts/Chnl]	3.0000	---	---
$Shift_{IRF}$[ps]	50.0000	---	---
$Period_{Rep}$[ns]	13.862	±0.085	0.6%
τ_{AvInt}[ns]	13.979	±0.028	0.2%
τ_{AvAmp}[ns]	13.979	±0.028	0.2%

Figure S45: Raw time-resolved photoluminescence decay of **N_1O_3** in DMSO (10 µM) with instrumental response function in red (left), including the residuals (λ_{ex} = 402.6 nm, λ_{em} = 495 nm); fitting parameters including pre-exponential factors and confidence limits (right).

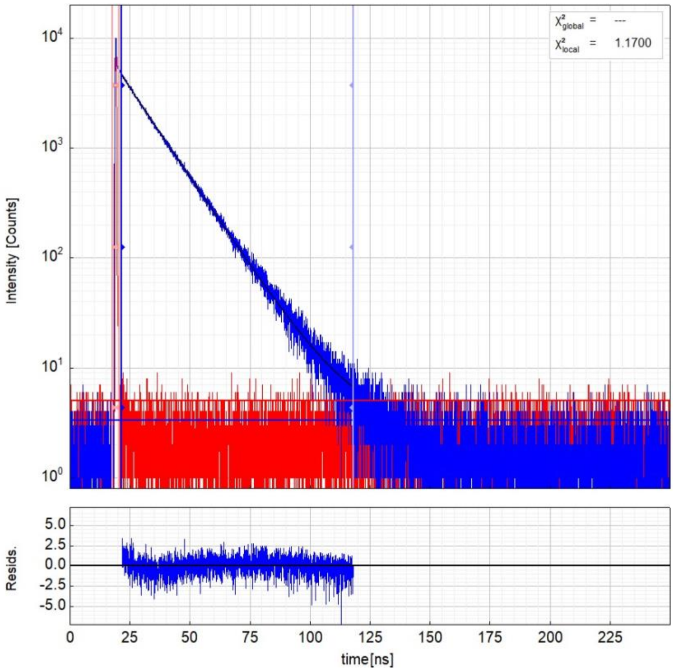

Parameter	Value	Δ	δ
A_1[kCnts/Chnl]	5.5012	±0.0084	0.2%
τ_1[ns]	13.387	±0.013	0.1%
I_1[kCnts]	2 301.4	±2.4	0.1%
A_{Rel1}[%]	100.0	---	---
I_{Rel1}[%]	100.0	---	---
$Bkgr_{Dec}$[kCnts]	0.0034	±0.0001	2.2%
$Bkgr_{IRF}$[Cnts/Chnl]	5.0000	---	---
$Shift_{IRF}$[ps]	130.0000	---	---
τ_{AvInt}[ns]	13.387	±0.013	0.1%
τ_{AvAmp}[ns]	13.387	±0.013	0.1%

Figure S46: Raw time-resolved photoluminescence decay of **N_1O_3** in the 3D-printed hexagon (0.1 wt%) with instrumental response function in red (left), including the residuals (λ_{ex} = 440.0 nm, λ_{em} = 489 nm); fitting parameters including pre-exponential factors and confidence limits (right).

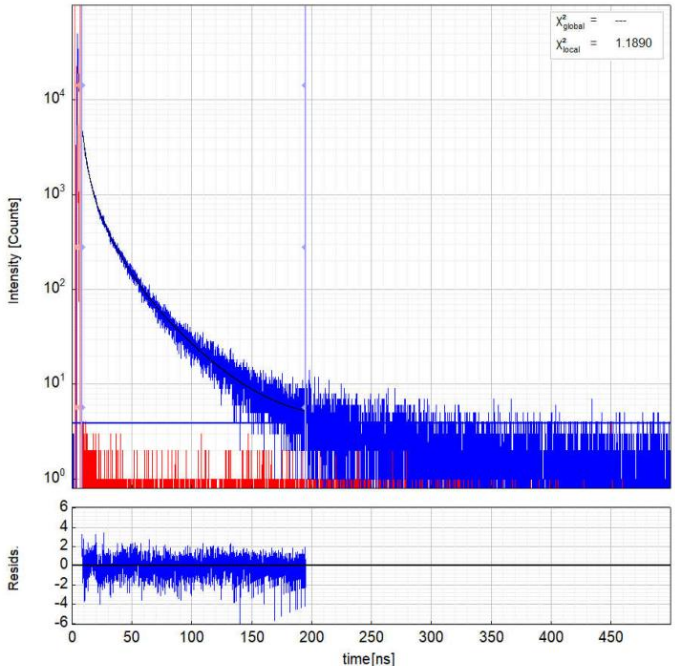

Parameter	Value	Δ	δ
A_1[kCnts/Chnl]	11.44	±0.69	6.0%
$τ_1$[ns]	3.10	±0.25	7.9%
I_1[kCnts]	554	±19	3.3%
A_{Rel1}[%]	83.3	±1.5	1.7%
I_{Rel1}[%]	49.7	±1.9	3.7%
A_2[kCnts/Chnl]	0.397	±0.097	24%
$τ_2$[ns]	33.3	±2.7	7.9%
I_2[kCnts]	207	±31	15%
A_{Rel2}[%]	2.9	±0.7	21%
I_{Rel2}[%]	18.5	±2.7	14%
A_3[kCnts/Chnl]	1.91	±0.20	10%
$τ_3$[ns]	12.0	±1.3	11%
I_3[kCnts]	356	±13	3.5%
A_{Rel3}[%]	14.0	±0.9	5.8%
I_{Rel3}[%]	31.9	±1.1	3.4%
$Bkgr_{Dec}$[kCnts]	0.0040	±0.0007	16%
$Bkgr_{IRF}$[Cnts/Chnl]	0.0000	---	---
$Shift_{IRF}$[ps]	100.0000	---	---
$τ_{AvInt}$[ns]	11.50	±0.38	3.2%
$τ_{AvAmp}$[ns]	5.20	±0.29	5.5%

Figure S47: Raw time-resolved photoluminescence decay of **N_2O_2** in the solid-state (blue) with instrumental response function in red (left), including the residuals (λ_{ex} = 505.7 nm, λ_{em} = 542 nm); fitting parameters including pre-exponential factors and confidence limits (right).

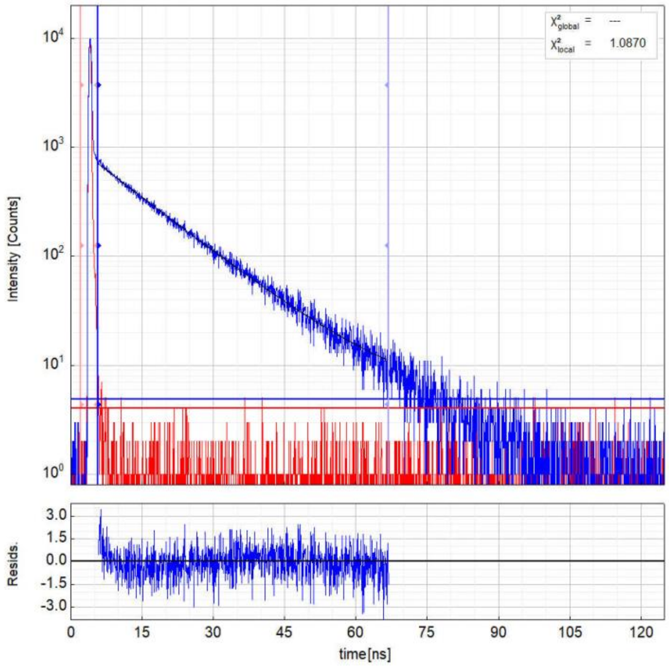

Parameter	Value	Δ	δ
A_1[kCnts/Chnl]	0.807	±0.012	1.4%
$τ_1$[ns]	12.93	±0.19	1.4%
I_1[kCnts]	162.83	±0.96	0.6%
A_{Rel1}[%]	100.0	---	---
I_{Rel1}[%]	100.0	---	---
$Bkgr_{Dec}$[kCnts]	0.0050	±0.0010	19%
$Bkgr_{IRF}$[Cnts/Chnl]	4.0000	---	---
$Shift_{IRF}$[ps]	0.0000	---	---
$τ_{AvInt}$[ns]	12.93	±0.19	1.4%
$τ_{AvAmp}$[ns]	12.93	±0.19	1.4%

Figure S48: Raw time-resolved photoluminescence decay of **N_2O_2** in the PMMA film (blue) with instrumental response function in red (left), including the residuals (λ_{ex} = 440.0 nm, λ_{em} = 510 nm); fitting parameters including pre-exponential factors and confidence limits (right).

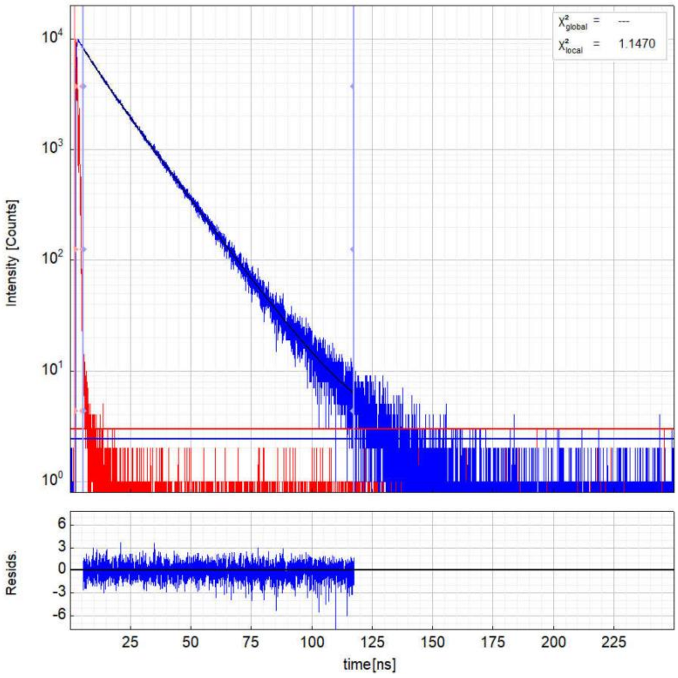

Parameter	Value	Δ	δ
A_1[kCnts/Chnl]	1.89	±0.25	13%
$τ_1$[ns]	6.51	±0.82	13%
I_1[kCnts]	390	±110	27%
A_{Rel1}[%]	18.7	±2.7	14%
I_{Rel1}[%]	9.1	±2.6	28%
A_2[kCnts/Chnl]	8.22	±0.32	3.9%
$τ_2$[ns]	14.94	±0.17	1.1%
I_2[kCnts]	3 840	±110	2.8%
A_{Rel2}[%]	81.4	±2.7	3.2%
I_{Rel2}[%]	91.0	±2.6	2.7%
$Bkgr_{Dec}$[kCnts]	0.0025	±0.0006	21%
$Bkgr_{IRF}$[Cnts/Chnl]	3.0000	---	---
$Shift_{IRF}$[ps]	50.0000	---	---
$τ_{AvInt}$[ns]	14.165	±0.032	0.2%
$τ_{AvAmp}$[ns]	13.357	±0.085	0.6%

Figure S49: Raw time-resolved photoluminescence decay of **N_2O_2** in DMSO solution (10 µM) with instrumental response function in red (left), including the residuals ($λ_{ex}$ = 402.6 nm, $λ_{em}$ = 545 nm); fitting parameters including pre-exponential factors and confidence limits (right).

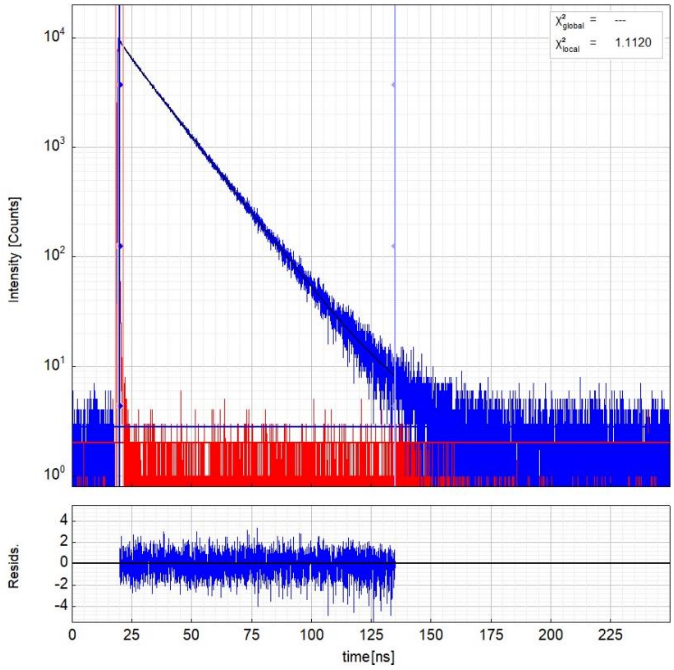

Parameter	Value	Δ	δ
A_1[kCnts/Chnl]	8.948	±0.067	0.7%
$τ_1$[ns]	15.609	±0.055	0.3%
I_1[kCnts]	4 365	±18	0.4%
A_{Rel1}[%]	91.0	±0.6	0.6%
I_{Rel1}[%]	97.2	±0.4	0.4%
A_2[kCnts/Chnl]	0.891	±0.052	5.8%
$τ_2$[ns]	4.59	±0.43	9.2%
I_2[kCnts]	128	±18	14%
A_{Rel2}[%]	9.1	±0.6	5.7%
I_{Rel2}[%]	2.9	±0.4	14%
$Bkgr_{Dec}$[kCnts]	0.0029	±0.0005	14%
$Bkgr_{IRF}$[Cnts/Chnl]	2.0000	---	---
$Shift_{IRF}$[ps]	300.0000	---	---
A_{Scatt}[kCnts]	280	±190	67%
$τ_{AvInt}$[ns]	15.296	±0.023	0.1%
$τ_{AvAmp}$[ns]	14.611	±0.042	0.3%

Figure S50: Raw time-resolved photoluminescence decay of **N_2O_2** in the 3D-printed hexagon (0.1 wt%) with instrumental response function in red (left), including the residuals ($λ_{ex}$ = 440.0 nm, $λ_{em}$ = 531 nm); fitting parameters including pre-exponential factors and confidence limits (right).

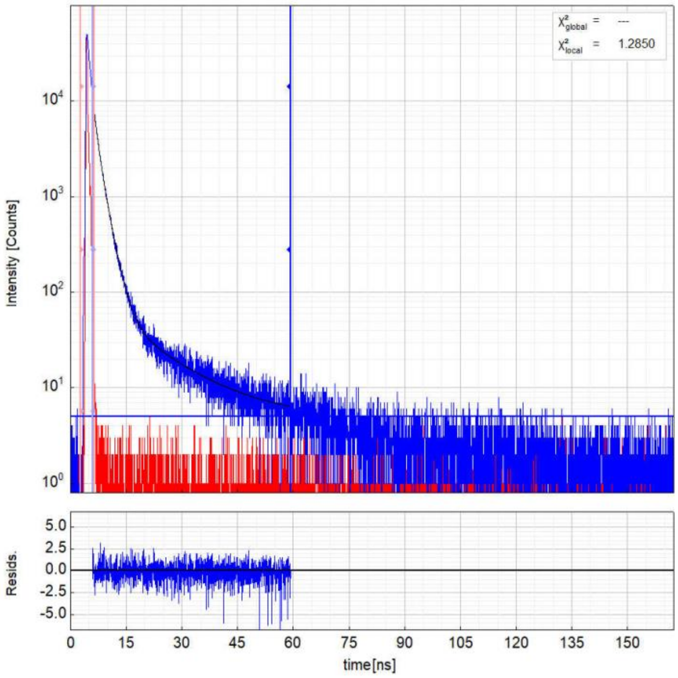

Parameter	Value	Δ	δ
A_1[kCnts/Chnl]	12.0	±1.3	11%
τ_1[ns]	2.006	±0.064	3.1%
I_1[kCnts]	751	±55	7.3%
A_{Rel1}[%]	26.6	±1.7	6.0%
I_{Rel1}[%]	44.9	±2.4	5.2%
A_2[kCnts/Chnl]	0.086	±0.019	21%
τ_2[ns]	13.5	±2.2	16%
I_2[kCnts]	35.9	±2.2	6.1%
A_{Rel2}[%]	0.2	±0.1	15%
I_{Rel2}[%]	2.2	±0.1	3.6%
A_3[kCnts/Chnl]	33.2	±2.0	5.9%
τ_3[ns]	0.858	±0.059	6.8%
I_3[kCnts]	888	±28	3.1%
A_{Rel3}[%]	73.4	±1.7	2.2%
I_{Rel3}[%]	53.1	±2.5	4.6%
$Bkgr_{Dec}$[kCnts]	0.0051	±0.0010	18%
$Bkgr_{IRF}$[Cnts/Chnl]	0.0000	---	---
$Shift_{IRF}$[ps]	35.0000	---	---
τ_{AvInt}[ns]	1.641	±0.064	3.9%
τ_{AvAmp}[ns]	1.186	±0.051	4.2%

Figure S51: Raw time-resolved photoluminescence decay of **N_3O_1** in the solid-state (blue) with instrumental response function in red (left), including the residuals (λ_{ex} = 505.7 nm, λ_{em} = 578 nm); fitting parameters including pre-exponential factors and confidence limits (right).

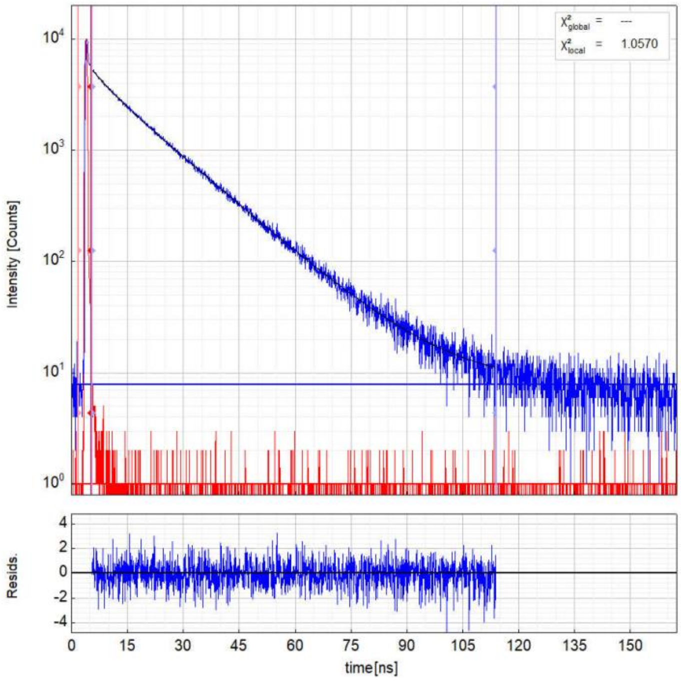

Parameter	Value	Δ	δ
A_1[kCnts/Chnl]	1.306	±0.070	5.3%
τ_1[ns]	4.43	±0.48	11%
I_1[kCnts]	91	±12	12%
A_{Rel1}[%]	21.0	±1.0	4.6%
I_{Rel1}[%]	7.3	±0.9	12%
A_2[kCnts/Chnl]	4.932	±0.086	1.7%
τ_2[ns]	15.035	±0.094	0.6%
I_2[kCnts]	1 159	±13	1.1%
A_{Rel2}[%]	79.1	±1.0	1.2%
I_{Rel2}[%]	92.8	±0.9	1.0%
$Bkgr_{Dec}$[kCnts]	0.0079	±0.0005	6.0%
$Bkgr_{IRF}$[Cnts/Chnl]	1.0000	---	---
$Shift_{IRF}$[ps]	0.0000	---	---
τ_{AvInt}[ns]	14.269	±0.037	0.3%
τ_{AvAmp}[ns]	12.82	±0.17	1.3%

Figure S52: Raw time-resolved photoluminescence decay of **N_3O_1** in the PMMA film (blue) with instrumental response function in red (left), including the residuals (λ_{ex} = 440.0 nm, λ_{em} = 560 nm); fitting parameters including pre-exponential factors and confidence limits (right).

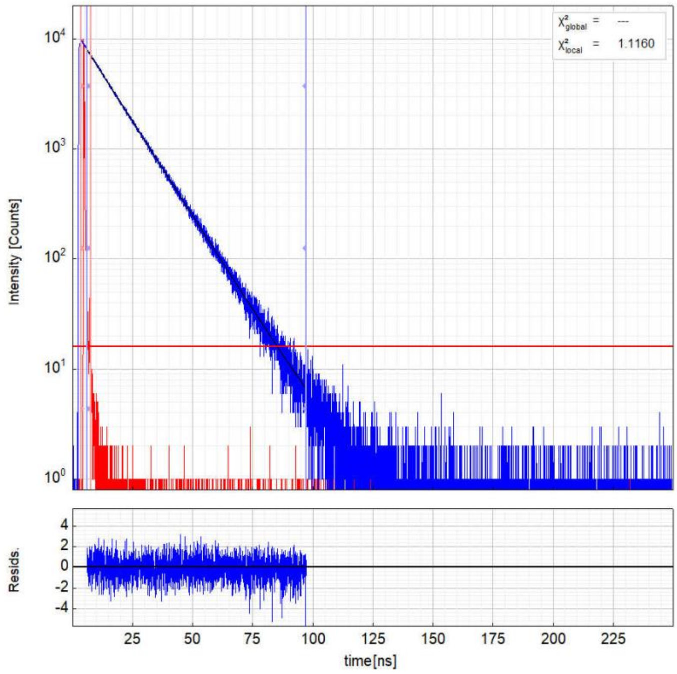

Parameter	Value	Δ	δ
A_1[kCnts/Chnl]	9.020	±0.078	0.9%
τ_1[ns]	12.665	±0.020	0.2%
I_1[kCnts]	3 570	±35	1.0%
A_{Rel1}[%]	100.0	---	---
I_{Rel1}[%]	100.0	---	---
$Bkgr_{Dec}$[kCnts]	0.0007	±0.0003	42%
$Bkgr_{IRF}$[Cnts/Chnl]	16.1	±6.1	38%
$Shift_{IRF}$[ps]	1 955	±97	4.9%
τ_{AvInt}[ns]	12.665	±0.020	0.2%
τ_{AvAmp}[ns]	12.665	±0.020	0.2%

Figure S53: Raw time-resolved photoluminescence decay of **N_3O_1** in DMSO solution (10 µM) with instrumental response function in red (left), including the residuals (λ_{ex} = 402.6 nm, λ_{em} = 603 nm); fitting parameters including pre-exponential factors and confidence limits (right).

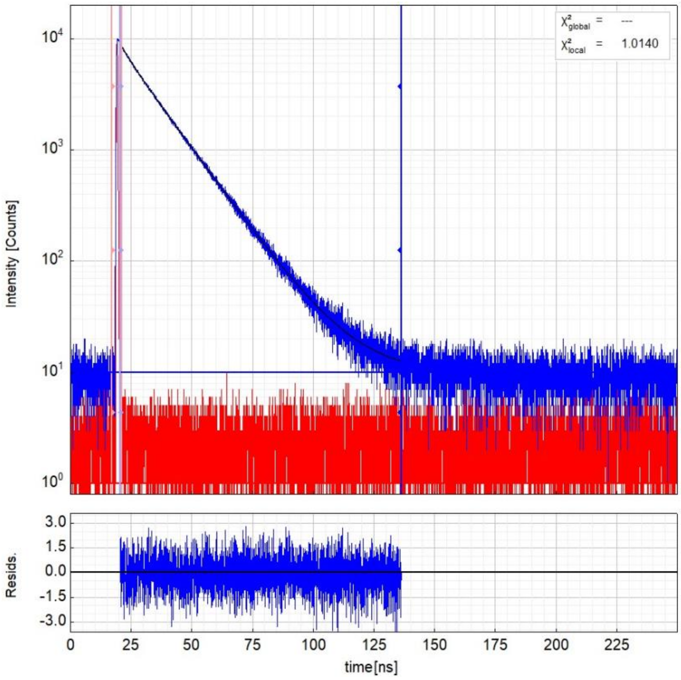

Parameter	Value	Δ	δ
A_1[kCnts/Chnl]	1.361	±0.042	3.1%
τ_1[ns]	4.42	±0.41	9.1%
I_1[kCnts]	188	±18	9.3%
A_{Rel1}[%]	13.7	±0.4	2.9%
I_{Rel1}[%]	4.6	±0.5	9.3%
A_2[kCnts/Chnl]	8.643	±0.058	0.7%
τ_2[ns]	14.504	±0.033	0.2%
I_2[kCnts]	3 917	±18	0.4%
A_{Rel2}[%]	86.4	±0.4	0.4%
I_{Rel2}[%]	95.5	±0.5	0.4%
$Bkgr_{Dec}$[kCnts]	0.0100	±0.0004	3.0%
$Bkgr_{IRF}$[Cnts/Chnl]	1.0000	---	---
$Shift_{IRF}$[ps]	200.0000	---	---
τ_{AvInt}[ns]	14.042	±0.015	0.1%
τ_{AvAmp}[ns]	13.132	±0.073	0.6%

Figure S54: Raw time-resolved photoluminescence decay of **N_3O_1** in the 3D-printed hexagon (0.1 wt%) with instrumental response function in red (left), including the residuals (λ_{ex} = 440.0 nm, λ_{em} = 582 nm); fitting parameters including pre-exponential factors and confidence limits (right).

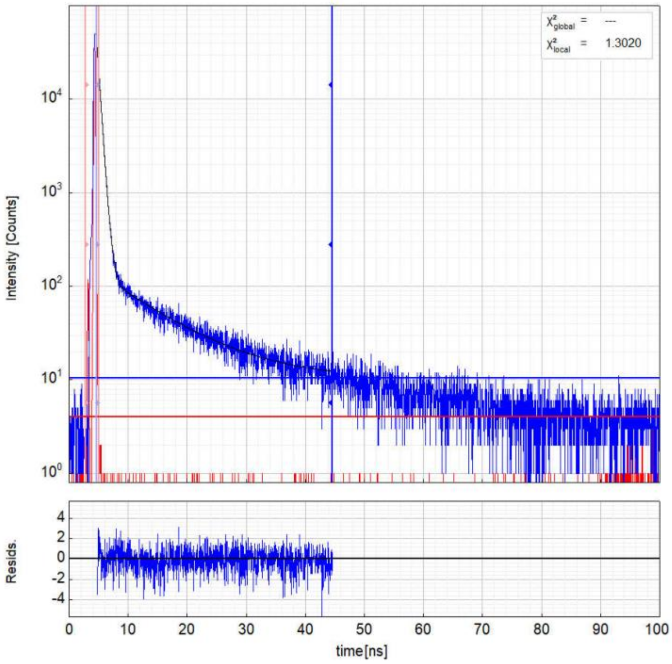

Parameter	Value	Δ	δ
A_1[kCnts/Chnl]	98.05	±0.83	0.8%
τ_1[ns]	0.4672	±0.0016	0.3%
I_1[kCnts]	1 431.2	±9.2	0.6%
A_{Rel1}[%]	99.9	±0.1	0.0%
I_{Rel1}[%]	97.3	±0.1	0.1%
A_2[kCnts/Chnl]	0.1362	±0.0046	3.3%
τ_2[ns]	9.53	±0.56	5.8%
I_2[kCnts]	40.6	±1.1	2.7%
A_{Rel2}[%]	0.2	±0.1	3.2%
I_{Rel2}[%]	2.8	±0.1	2.6%
$Bkgr_{Dec}$[kCnts]	0.0105	±0.0011	10.0%
$Bkgr_{IRF}$[Cnts/Chnl]	4.0000	---	---
$Shift_{IRF}$[ps]	10.0000	---	---
τ_{AvInt}[ns]	0.717	±0.022	3.1%
τ_{AvAmp}[ns]	0.4797	±0.0016	0.3%

Figure S55: Raw time-resolved photoluminescence decay of **N4** in the solid-state (blue) with instrumental response function in red (left), including the residuals (λ_{ex} = 505.7 nm, λ_{em} = 578 nm); fitting parameters including pre-exponential factors and confidence limits (right).

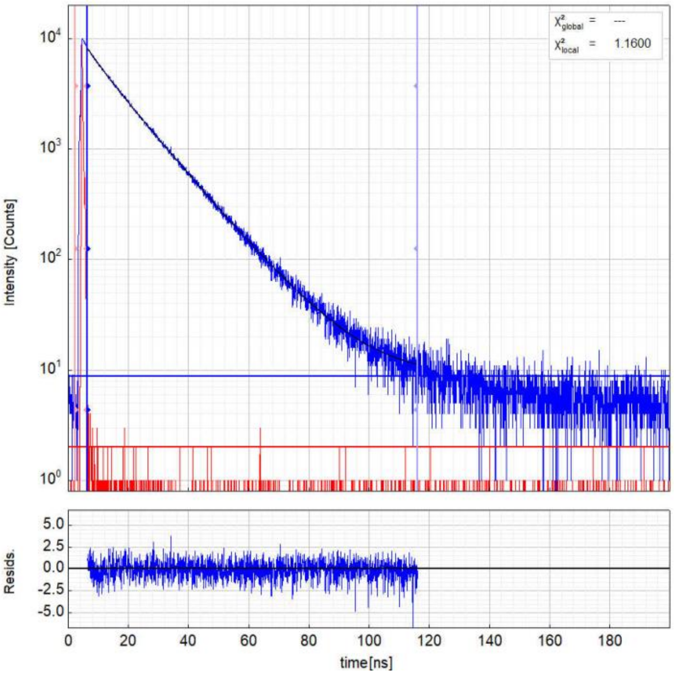

Parameter	Value	Δ	δ
A_1[kCnts/Chnl]	7.4	±1.8	24%
τ_1[ns]	13.96	±0.87	6.2%
I_1[kCnts]	1 600	±310	19%
A_{Rel1}[%]	77	±18	23%
I_{Rel1}[%]	88	±17	19%
A_2[kCnts/Chnl]	2.3	±1.6	72%
τ_2[ns]	6.7	±2.4	35%
I_2[kCnts]	230	±310	132%
A_{Rel2}[%]	24	±18	75%
I_{Rel2}[%]	13	±17	132%
$Bkgr_{Dec}$[kCnts]	0.0088	±0.0014	15%
$Bkgr_{IRF}$[Cnts/Chnl]	2.0000	---	---
$Shift_{IRF}$[ps]	200.0000	---	---
τ_{AvInt}[ns]	13.040	±0.082	0.6%
τ_{AvAmp}[ns]	12.28	±0.20	1.6%

Figure S56: Raw time-resolved photoluminescence decay of **N4** in the PMMA film (blue) with instrumental response function in red (left), including the residuals (λ_{ex} = 505.7 nm, λ_{em} = 560 nm); fitting parameters including pre-exponential factors and confidence limits (right).

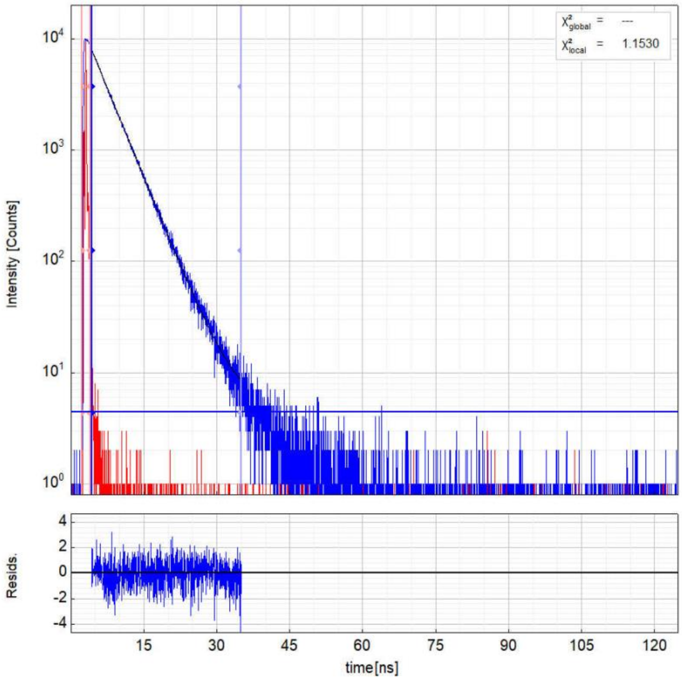

Parameter	Value	Δ	δ
A_1[kCnts/Chnl]	9.653	±0.028	0.3%
τ_1[ns]	4.0572	±0.0089	0.2%
I_1[kCnts]	1 223.8	±3.2	0.3%
A_{Rel1}[%]	100.0	---	---
I_{Rel1}[%]	100.0	---	---
$Bkgr_{Dec}$[kCnts]	0.0045	±0.0006	13%
$Bkgr_{IRF}$[Cnts/Chnl]	0.0000	---	---
$Shift_{IRF}$[ps]	0.0000	---	---
$Period_{Rep}$[ns]	6.871	±0.066	0.9%
τ_{AvInt}[ns]	4.0572	±0.0089	0.2%
τ_{AvAmp}[ns]	4.0572	±0.0089	0.2%

Figure S57: Raw time-resolved photoluminescence decay of **N4** in DMSO solution (10 µM) with instrumental response function in red (left), including the residuals (λ_{ex} = 402.6 nm, λ_{em} = 652 nm); fitting parameters including pre-exponential factors and confidence limits (right).

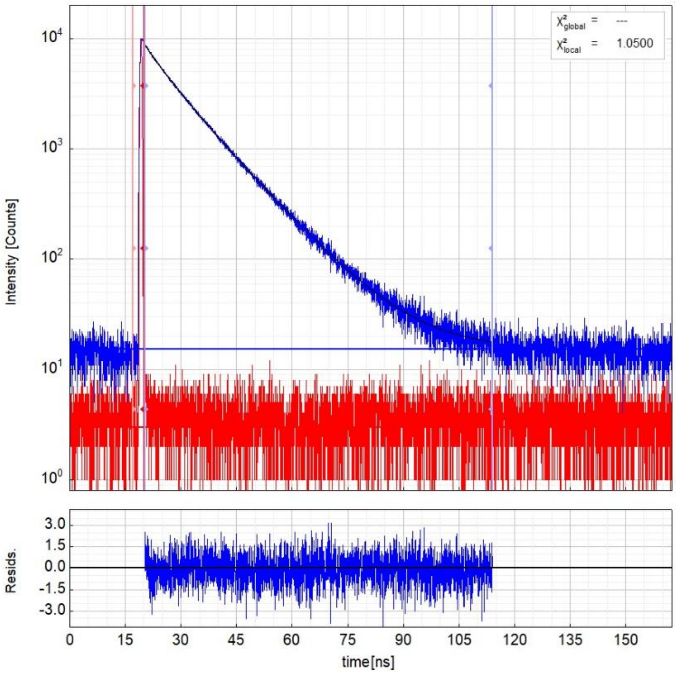

Parameter	Value	Δ	δ
A_1[kCnts/Chnl]	7.02	±0.22	3.1%
τ_1[ns]	11.91	±0.12	0.9%
I_1[kCnts]	2 610	±59	2.2%
A_{Rel1}[%]	70.8	±2.0	2.8%
I_{Rel1}[%]	84.8	±2.0	2.3%
A_2[kCnts/Chnl]	2.91	±0.19	6.3%
τ_2[ns]	5.17	±0.31	6.0%
I_2[kCnts]	469	±60	13%
A_{Rel2}[%]	29.3	±2.0	6.7%
I_{Rel2}[%]	15.3	±2.0	13%
$Bkgr_{Dec}$[kCnts]	0.0154	±0.0003	1.5%
$Bkgr_{IRF}$[Cnts/Chnl]	3.0000	---	---
$Shift_{IRF}$[ps]	200.0000	---	---
τ_{AvInt}[ns]	10.878	±0.026	0.2%
τ_{AvAmp}[ns]	9.930	±0.041	0.4%

Figure S58: Raw time-resolved photoluminescence decay of **N4** in the 3D-printed hexagon (0.1 wt%) with instrumental response function in red (left), including the residuals (λ_{ex} = 440.0 nm, λ_{em} = 617 nm); fitting parameters including pre-exponential factors and confidence limits (right).

4 X-RAY DIFFRACTOMETRIC ANALYSIS ON SINGLE CRYSTALS

Table S11: Crystallographic data.

**Identification code**	**O_4**	**N_2O_2**	**N_3O_1**	**N_4**
CCDC number	2393406	2393407	2393405	2393408
Name in cif	jv_ev_1m	alh_atj02m	alh_atj01m	alh_ev310m
Empirical formula	$C_{12}H_8N_2O_4$	$C_{14}H_{14}N_4O_2$	$C_{15}H_{17}N_5O$	$C_{16}H_{20}N_6$
M	244.20	270.29	283.33	296.38
Crystal size [mm]	0.182×0.069×0.053	0.410×0.207×0.168	0.416×0.134×0.088	0.155×0.124×0.024
T [K]	100(2)	100(2)	100(2)	100(2)
Crystal system	monoclinic	orthorhombic	monoclinic	triclinic
Space group	$P2_1/n$	*Pnma*	$P2_1/c$	*P*-1
a [Å]	7.7502(5)	16.4001(9)	17.0733(7)	5.8597(4)
b [Å]	5.0152(3)	8.5277(4)	9.3910(4)	7.5221(5)
c [Å]	13.5472(8)	8.9140(5)	8.4521(3)	9.2463(6)
α [°]	90	90	90	75.4147(17)
β [°]	99.0197(15)	90	91.8137(12)	72.6291(16)
γ [°]	90	90	90	86.8520(18)
V [Å^3]	520.05(6)	1246.67(11)	1354.49(9)	376.34(4)
Z	2	4	4	1
D_{calc} [g·cm^{-3}]	1.559	1.440	1.389	1.308
μ(CuK_α [mm^{-1}])	1.018	0.824	0.747	0.662
Transmissions	0.75/0.64	0.75/0.65	0.75/0.60	0.75/0.61
F(000)	252	568	600	158
Index ranges	$-9 \leq h \leq 9$	$-20 \leq h \leq 20$	$-21 \leq h \leq 21$	$-7 \leq h \leq 7$
	$-6 \leq k \leq 6$	$-10 \leq k \leq 10$	$-11 \leq k \leq 11$	$-9 \leq k \leq 9$
	$-17 \leq l \leq 17$	$-11 \leq l \leq 11$	$-9 \leq l \leq 10$	$-11 \leq l \leq 10$
θ_{max} [°]	80.235	79.420	80.226	80.591
Reflections collected	20777	67687	77391	20641
Independent reflections	1140	1451	2960	1638
R_{int}	0.0296	0.0426	0.0323	0.0323
Refined parameters	82	183	193	102
R_1 [$I > 2\sigma(I)$]	0.0275	0.0361	0.0448	0.0321
wR_2 [all data]	0.0751	0.0931	0.1202	0.0936
x(Flack)				
GooF	1.105	1.122	1.173	1.097
$\Delta\rho_{final}$ (max/min) [e·Å^{-3}]	0.251/-0.186	0.201/-0.212	0.383/-0.247	0.270/-0.179

Table S12: Interactions in **N_3O_1** [Å]. Operators for generating equivalent atoms: $1 x, y+1, z; $2 x, -y+1/2, z-1/2; $3 -x, y+1/2, -z-1/2; $4 -x+1, y-1/2, -z+1/2; $5 x, -y+1/2, z+1/2; $6 x, -y+1/2, z-1/2.

D-H	H···A	D···A	<(DHA) [°]	
0.99	2.54	3.512(2)	168.4	C9-H9A...N1_$1
0.99	2.67	3.377(2)	128.9	C11-H11A...N2_$2
0.98	2.69	3.628(2)	161.1	C14-H14A...N1_$3
0.98	2.69	3.6019(19)	154.7	C15-H15B...N2_$4
0.99	2.79	3.6972(19)	153.1	C12-H12B...N2_$4
0.99	2.79	3.741(2)	160.7	C9-H9B...N1_$5
0.99	2.70	3.673(2)	167.4	C12-H12A...C1_$5
0.98	2.89	3.820(2)	159.1	C14-H14C...C3_$6
0.98	2.92	3.692(2)	136.3	C14-H14C...C4_$6

Table S13: Interactions in **N_4** [Å]. Operators for generating equivalent atoms: $1 x+1, y-1, z; $2 -x, -y+1, -z+2; $3 -x+1, -y, -z+1; $4 -x+1, -y+1, -z+1.

D-H	H···A	D···A	<(DHA) [°]		
0.99	2.63	3.6097(13)	171.5		C4-H4A···N1_$1
0.99	2.57	3.5104(13)	158.2		C5-H5A···N1_$2
0.98	2.64	3.5004(12)	146.4		C7-H7B···N2_$3
D-H	H···A	D···A	<(DHA)	<(H/Cnt/Plane)	
0.98	2.64	3.4573(10)	140.5	80.0	C7-H7C···CnT1_$4

Table S14: Interactions in **O_4** [Å]. Operators for generating equivalent atoms: $1 x+1, y-1, z; $2 -x+5/2, y-1/2, -z+3/2; $3 x, y+1, z; $4 -x+2, -y+1, -z+1.

D-H	H···A	D···A	<(DHA) [°]	
0.99	2.57	3.4127(12)	142.6	C6-H6A···N1_$1
0.99	2.50	3.4346(11)	157.4	C6-H6B···O1_$2
0.99	2.69	3.3671(12)	126.3	C5-H5B···O2_$3
		3.3924(12)		N1···C3_$4

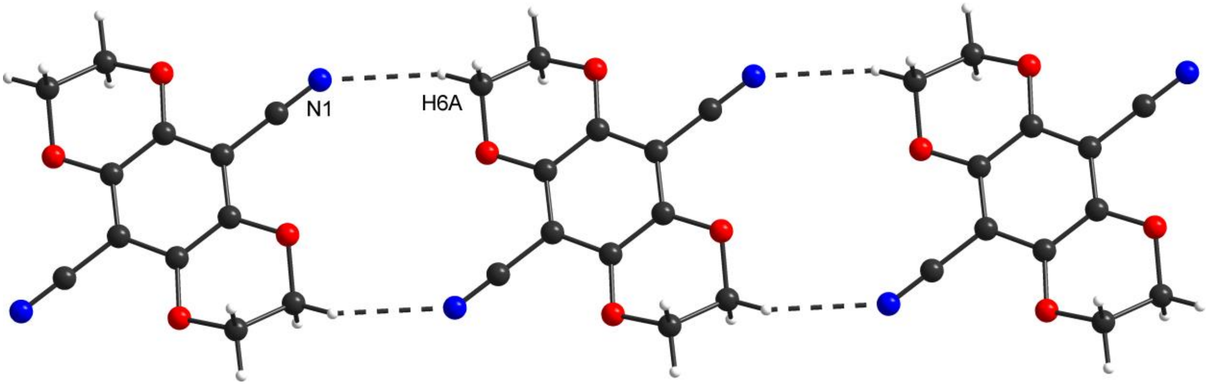

Figure S59: String of molecules in the packing of **O4**.

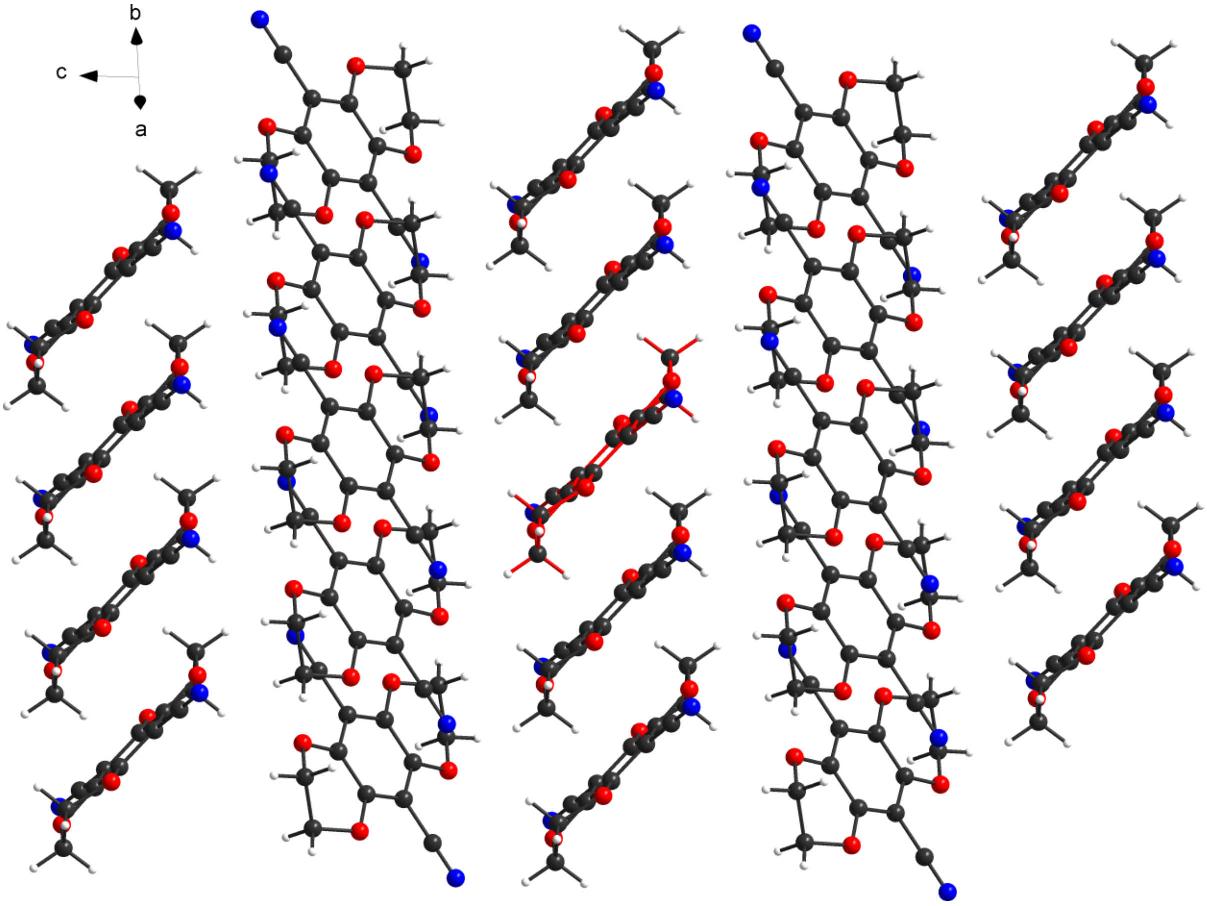

Figure S60: Packing of **O4**. One of the strings high-lighted in red bonds.

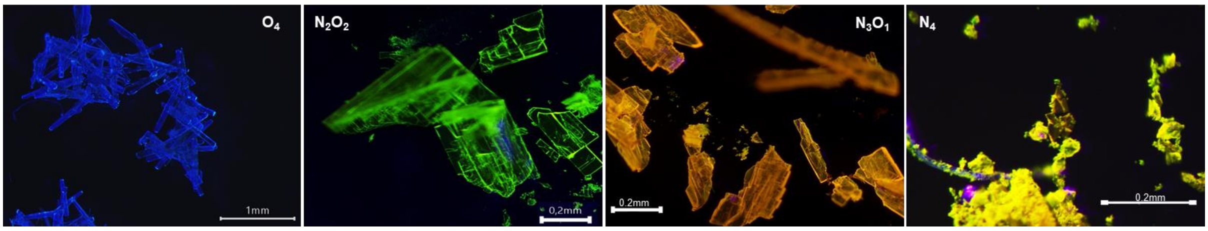

Figure S61: Microscope pictures of the crystalline solids taken under 365 nm UV light.

5 CRYSTALEXPLORER

Further analysis of occurring interactions in the crystal packing were carried out using Hirshfeld Surface (HS) analysis and the corresponding Fingerprint plots (FPP). HS calculations using the parameter d_{norm} were performed with *CrystalExplorer17* program.[15] Reciprocal contacts are included in the FP plots.

Table S15: Display of all interactions in the crystal lattices determined by HS analysis. Reciprocal contacts are summarized.

Interaction	O_4	N_3O_1	N_4
O-O	1.4%	0.0%	-
O-N	0.0%	0.0%	-
O-C	2.6%	1.2%	-
O-H	25.9%	5.2%	-
N-N	0.3%	0.1%	0.0%
N-C	5.6%	0.3%	0.0%
N-H	33.1%	31.0%	35.5%
C-C	5.8%	4.0%	0.0%
C-H	12.4%	12.0%	16.3%
H-H	13.0%	46.2%	48.2%

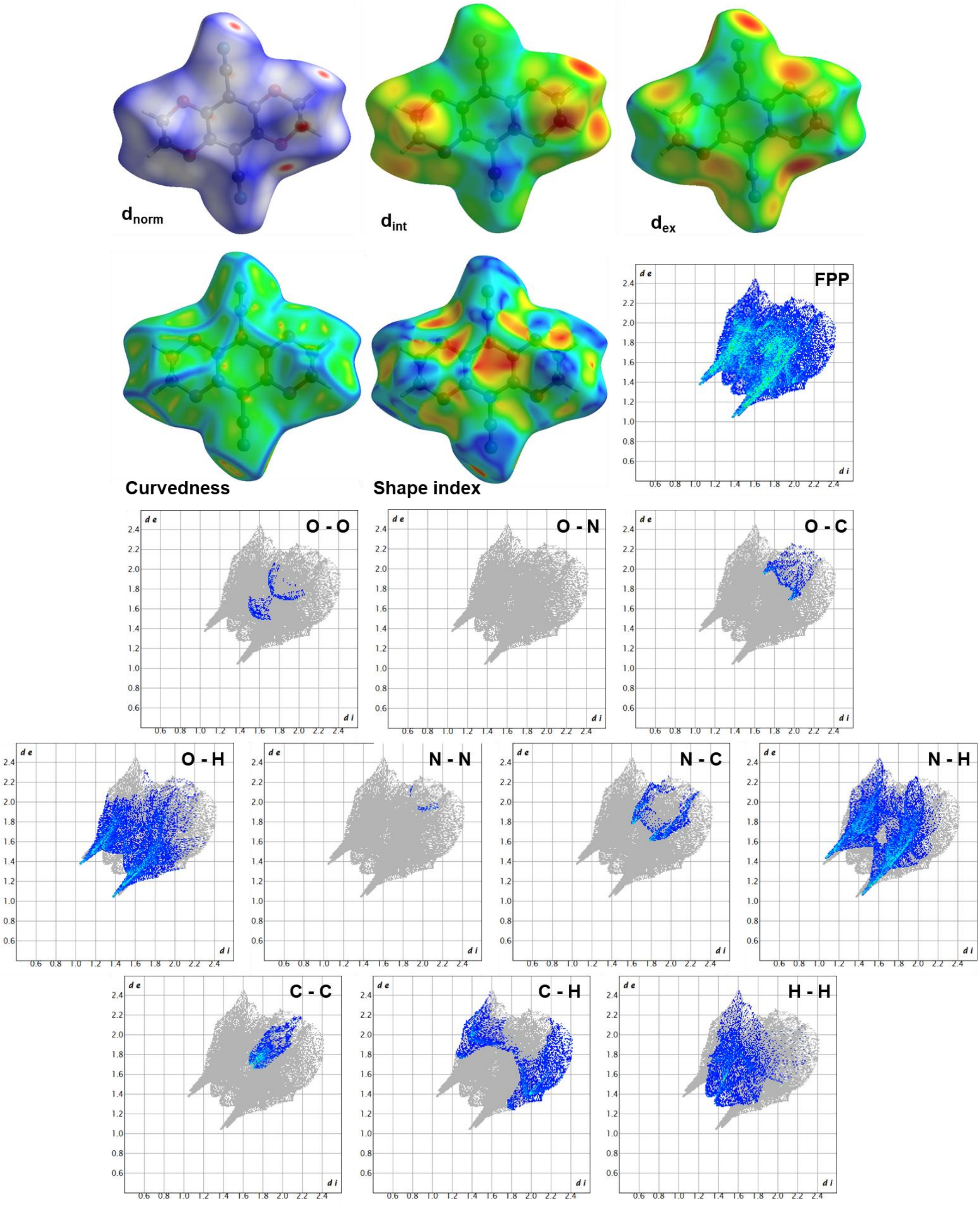

Figure S62: Hirshfeld surface of **O4** mapped with d_{norm} (normalised contact distance), d_{int}, d_{ex}, curvedness and shape index; Fingerprint plot for **O4** resolved into the contacts of all elements contained. Reciprocal contacts are included.

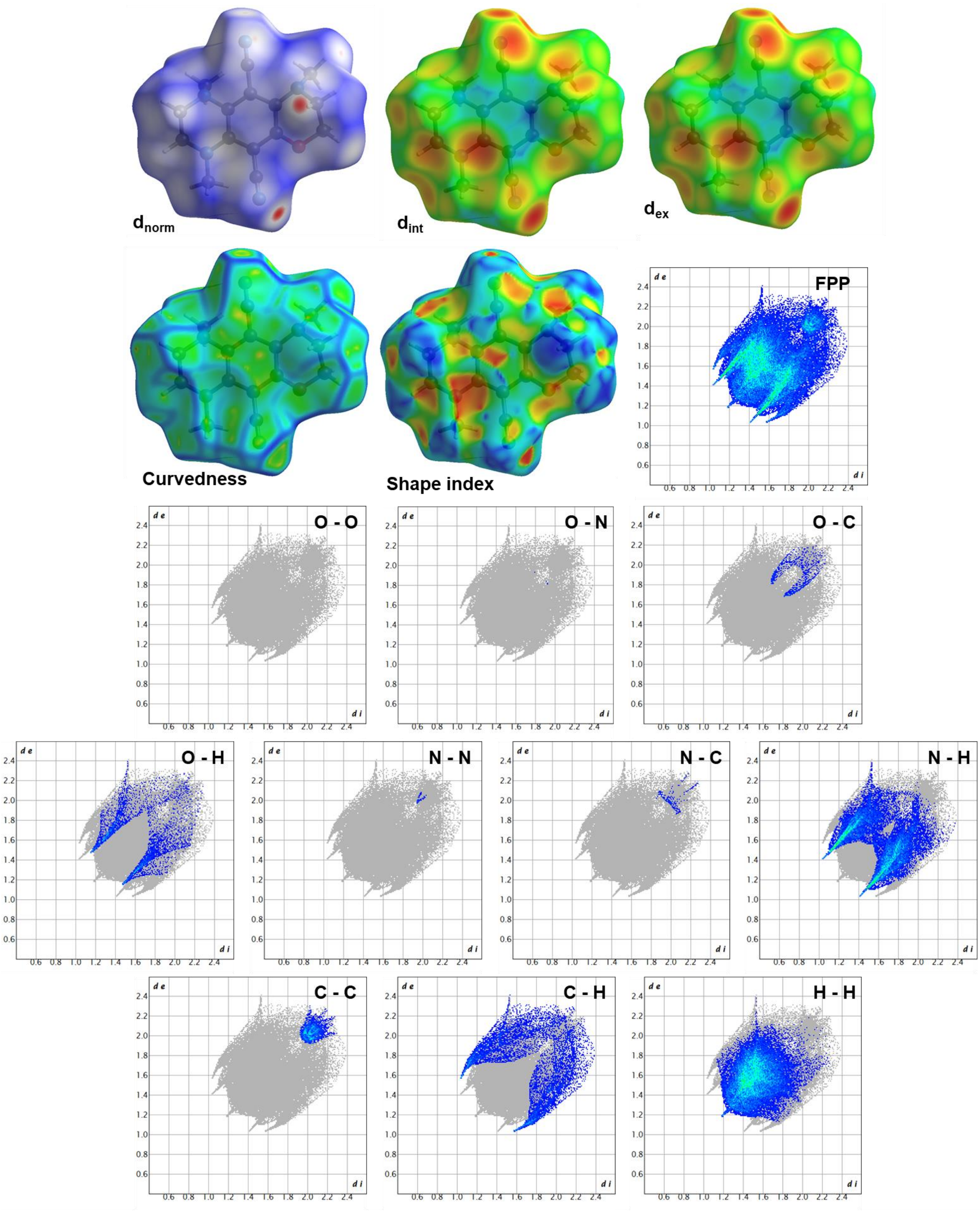

Figure S63: Hirshfeld surface of **N_3O_1** mapped with d_{norm} (normalised contact distance), d_{int}, d_{ex}, curvedness and shape index; Fingerprint plot for **N_3O_1** resolved into the contacts of all elements contained. Reciprocal contacts are included.

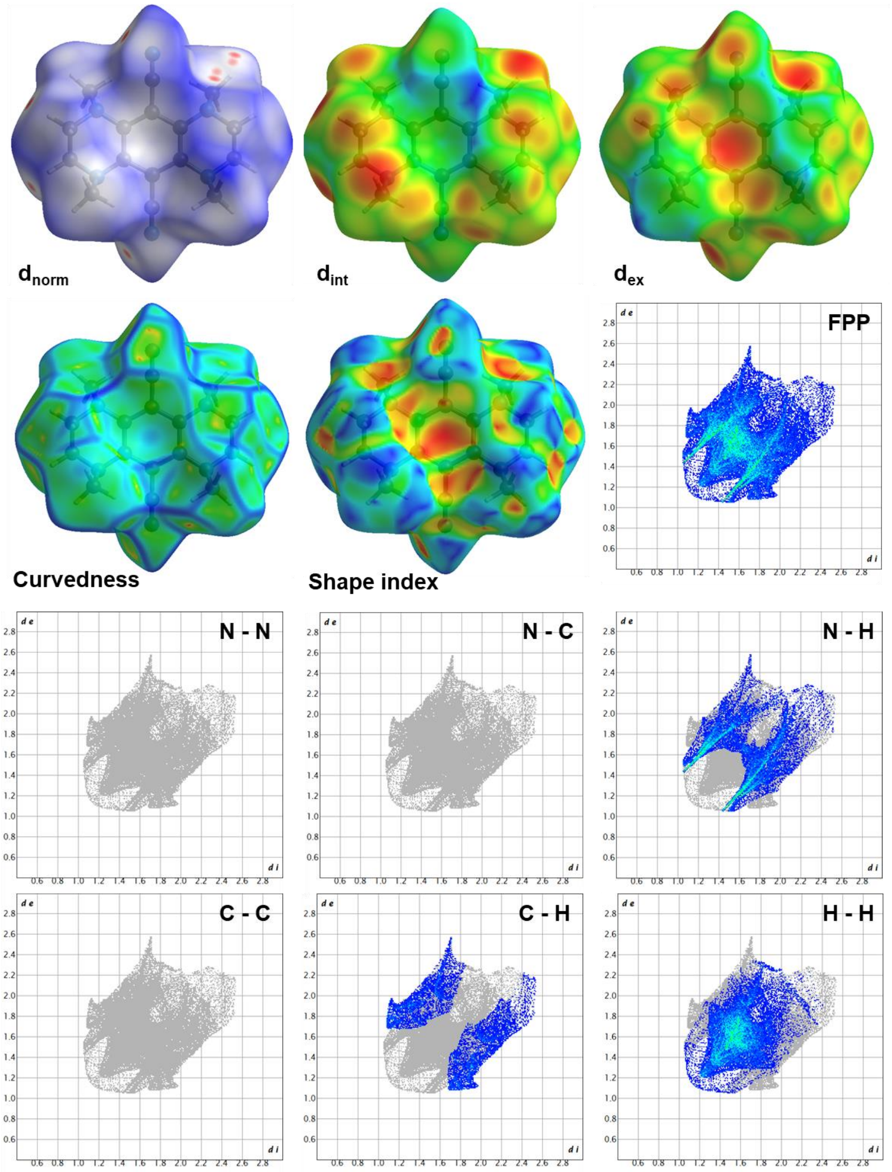

Figure S64: Hirshfeld surface of **N4** mapped with d_{norm} (normalised contact distance), d_{int}, d_{ex}, curvedness and shape index; Fingerprint plot for **N4** resolved into the contacts of all elements contained. Reciprocal contacts are included.

6 QUANTUM CHEMICAL CALCULATIONS

Table S16: Overview of the measured and calculated absorption and emission wavelengths using PBE0-GD3BJ/TZVP with the PCM method (solvent = dimethyl sulfoxide). Both conformers (all-equatorial and all-axial) were calculated and averaged.

		O_4	N_1O_3	N_2O_2	N_3O_1	N_4
λ_{ab} [nm]	Measured	389	410	437	450	467
	Equatorial	366	413	439	476	498
	Axial		397	408	427	437
	Averaged	-	405	424	452	468
λ_{em} [nm]	Measured	433	495	545	603	652
	Equatorial	436	493	571	643	669
	Axial		502	531	585	600
	Averaged	-	498	551	614	635

CARTESIAN COORDINATES OF THE OPTIMIZED GEOMETRIES

Cartesian coordinates of the optimized geometry of O_4 (S_0) using PBE0-GD3BJ/TZVP with the PCM method (solvent = dimethyl sulfoxide).

C	0.64963400	-0.37912000	3.51598100
C	-0.64963400	0.37912000	3.51598100
O	1.41764600	-0.03726900	2.35911300
C	0.69660400	-0.01414400	1.22054200
C	-0.69660400	0.01414400	1.22054200
O	-1.41764600	0.03726900	2.35911300
C	1.38089700	-0.00001500	0.00000100
C	0.69660100	0.01412800	-1.22054600
C	-0.69660100	-0.01412800	-1.22054600
C	-1.38089700	0.00001500	0.00000100
O	1.41765200	0.03727100	-2.35911600
O	-1.41765200	-0.03727100	-2.35911600
C	0.64963400	0.37912200	-3.51597800
C	-0.64963400	-0.37912200	-3.51597800
C	-2.79912100	-0.00001200	0.00000100
N	-3.95205300	-0.00002700	0.00000000
C	2.79912100	0.00001200	0.00000100
N	3.95205300	0.00002700	0.00000000
H	0.46779700	-1.45766200	3.51829500
H	1.25693500	-0.11110800	4.37815000
H	-0.46779700	1.45766200	3.51829500
H	-1.25693500	0.11110800	4.37815000
H	1.25693800	0.11112100	-4.37814800
H	0.46778900	1.45766300	-3.51828300
H	-0.46778900	-1.45766300	-3.51828300
H	-1.25693800	-0.11112100	-4.37814800

Cartesian coordinates of the optimized geometry of N_1O_3 (S_0, equatorial) using PBE0-GD3BJ/TZVP with the PCM method (solvent = dimethyl sulfoxide).

C	-1.19488400	-0.31609100	-0.02759800

C	-0.11770600	-1.22830300	0.05194400
C	-0.85608800	1.05712100	-0.03694900
C	1.22590400	-0.80169400	-0.02176800
C	-0.30294300	-2.61053100	0.30252600
N	-2.50730200	-0.68560500	-0.08968400
C	0.47030800	1.46797600	-0.05915200
O	-1.80987100	2.01408300	-0.04158500
C	1.52756800	0.54509800	-0.07838200
O	2.18574300	-1.75355500	0.00821300
N	-0.39902300	-3.73171000	0.56173800
C	-3.52715800	0.34603200	-0.09813600
C	-2.92487900	-1.97343300	-0.60697600
C	0.76313100	2.85672000	-0.06065600
C	-3.02149100	1.58541300	0.58232000
O	2.79785800	1.01575000	-0.12035500
C	3.47878600	-1.27505400	-0.36566300
H	-3.82617400	0.58159700	-1.12742400
H	-4.40868700	-0.02115400	0.43426200
H	-3.02373700	-2.72858800	0.17539900
H	-2.22360300	-2.33579600	-1.36155700
H	-3.89585900	-1.84818800	-1.08672500
N	1.00796000	3.98344100	-0.05782900
H	-3.72902700	2.40439400	0.47614100
H	-2.83259400	1.40491400	1.64502500
C	3.75826400	0.04679900	0.29844700
H	4.19160600	-2.03205700	-0.04402100
H	3.52194500	-1.17347900	-1.45414900
H	4.73427500	0.42944700	0.00611400
H	3.71768800	-0.05450900	1.38744000

Cartesian coordinates of the optimized geometry of N_1O_3 (S_0, axial) using PBE0-GD3BJ/TZVP with the PCM method (solvent = dimethyl sulfoxide).

C	-3.37765300	0.18188100	-0.64446700
C	-3.17067700	1.47672500	0.10592400
N	-2.46438600	-0.84272700	-0.16194900
C	-1.15261700	-0.37211000	-0.06849500
C	-0.87766600	0.99998400	-0.07170100
C	-0.05661900	-1.25650700	-0.05712500
C	1.26628300	-0.81021800	0.00233900
C	1.52346000	0.55942200	0.01307000
C	0.44958100	1.45161000	-0.04415300
C	3.53874300	-1.18852400	-0.30882200
C	3.76420300	0.09821100	0.43676900
C	-2.97859400	-1.59551600	0.97714600
C	0.70896300	2.84569700	-0.01963600
N	0.92172900	3.97889800	0.00125600
C	-0.27532500	-2.65185900	-0.19463200
N	-0.44660000	-3.78464300	-0.32820600
H	-3.20419900	0.34612500	-1.71153800
H	-4.40212100	-0.16803300	-0.52028000
H	-3.33228800	1.33712000	1.17956700
H	-3.82246600	2.27159000	-0.25198000
H	3.59178600	-1.02320200	-1.38888900
H	4.27204000	-1.93886200	-0.02036200
H	4.72695400	0.53510800	0.17944800
H	3.71556000	-0.06742200	1.51682800

H	-3.87018600	-2.14170700	0.66677000
H	-3.23761500	-0.94336100	1.82225600
H	-2.24261600	-2.32026000	1.31861300
O	2.77305500	1.06064700	0.06657100
O	2.25686400	-1.72769400	0.02116500
O	-1.83523700	1.94447400	-0.10341500

Cartesian coordinates of the optimized geometry of N_2O_2 (S_0, equatorial) using PBE0-GD3BJ/TZVP with the PCM method (solvent = dimethyl sulfoxide).

C	0.94254100	0.74446600	0.05376600
C	0.97129300	-0.69093200	0.03536600
C	-0.30295500	1.38658800	0.04839900
N	2.16450400	-1.33860200	0.15881400
C	-0.25081400	-1.37909000	-0.06607800
N	2.16422300	1.36721000	0.08250700
C	-1.51798500	0.65947900	0.10089000
C	-0.46286400	2.79043300	-0.08603000
C	2.28657000	-2.65880600	0.73462800
C	3.42497000	-0.67036100	-0.09095700
C	-1.49205800	-0.71035200	0.03930700
C	-0.31643800	-2.76122300	-0.36449000
C	3.19031900	0.68985800	-0.68926200
C	2.34717700	2.77460800	0.33748900
O	-2.67785400	1.36719400	0.15606700
N	-0.67501300	3.91574600	-0.23430300
H	3.20538800	-2.68638800	1.32506400
H	2.33782300	-3.45088500	-0.01760300
H	1.45021000	-2.86293400	1.40324900
H	3.97528600	-0.57168800	0.85233200
H	4.03225100	-1.28069800	-0.77002600
O	-2.61739800	-1.46905700	0.02697300
N	-0.41281600	-3.87130200	-0.67013000
H	2.89555000	0.60964800	-1.74364000
H	4.11019800	1.27027300	-0.64274900
H	1.70248700	3.09471600	1.15505100
H	3.38136000	2.92726400	0.64938700
H	2.15283200	3.40721500	-0.53580700
C	-3.80697700	0.60255900	-0.25789400
C	-3.78715800	-0.74814400	0.40842000
H	-3.79012600	0.49093100	-1.34689900
H	-4.69072100	1.16696000	0.03454600
H	-4.63864300	-1.35055900	0.09622100
H	-3.80188100	-0.63480600	1.49702500

Cartesian coordinates of the optimized geometry of N_2O_2 (S_0, axial) using PBE0-GD3BJ/TZVP with the PCM method (solvent = dimethyl sulfoxide).

C	3.28062200	0.64884000	-0.39487200
C	3.28061400	-0.64884800	0.39489500
N	2.11189600	1.44078600	-0.04411000
C	0.92876300	0.70468900	-0.00714700
C	0.92876100	-0.70469300	0.00714900
N	2.11189500	-1.44079500	0.04411500
C	-0.31299300	1.37223000	-0.06601000
C	-1.53364200	0.69488900	-0.03920500
C	-1.53364200	-0.69489000	0.03920600
C	-0.31299500	-1.37223200	0.06601000
C	-3.82863300	0.63009500	-0.41098000

C	-3.82863100	-0.63009400	0.41098800
C	2.35921400	-2.34793500	-1.07228200
C	2.35921000	2.34795800	1.07226200
C	-0.35503800	-2.77458800	0.27953400
N	-0.40195900	-3.90970100	0.48080600
C	-0.35503800	2.77458400	-0.27953900
N	-0.40196400	3.90969600	-0.48081800
H	3.26220200	0.43053500	-1.46706000
H	4.17251000	1.24161700	-0.19303400
H	3.26217300	-0.43054100	1.46708200
H	4.17250600	-1.24162500	0.19307400
H	-3.82853600	0.39344600	-1.47911900
H	-4.69268100	1.24885800	-0.17679300
H	-4.69268200	-1.24885600	0.17680700
H	-3.82852600	-0.39344500	1.47912700
H	1.44781800	-2.86545800	-1.36152400
H	3.09080200	-3.09785700	-0.76832200
H	2.74361500	-1.81468100	-1.95231900
H	3.09075100	3.09791300	0.76826600
H	2.74367000	1.81473400	1.95229000
H	1.44779900	2.86543700	1.36153000
O	-2.67437100	-1.41450900	0.10344100
O	-2.67437000	1.41450900	-0.10344200

Cartesian coordinates of the optimized geometry of N_3O_1 (S_0, equatorial) using PBE0-GD3BJ/TZVP with the PCM method (solvent = dimethyl sulfoxide).

C	-3.46803100	0.05011600	0.58228900
C	-3.34808600	-1.34919400	0.04035700
N	-2.60214700	0.92621000	-0.18608900
C	-1.26869000	0.60421800	-0.09653500
C	-0.95804900	-0.78848600	-0.03257500
N	-1.95708500	-1.71004300	-0.14074700
C	-0.19983800	1.50965900	-0.04422000
C	1.14645700	1.07859400	-0.09034000
C	1.46998100	-0.26370400	-0.03676600
C	0.38795200	-1.17382900	0.13557000
N	2.79501800	-0.70008900	-0.04012500
C	3.30578700	1.61324500	0.52018300
C	3.79377800	0.35339300	-0.13755100
C	-1.74185400	-3.04067600	-0.66209200
C	-3.08659900	2.25726400	-0.45509600
C	3.14251000	-1.81444200	-0.90942900
C	0.66707400	-2.44580400	0.68232900
N	0.89298400	-3.44878300	1.21263000
C	-0.36939900	2.91203000	0.09969800
N	-0.43381700	4.05350900	0.26161100
H	-3.20841100	0.08129500	1.64893000
H	-4.49768600	0.38918200	0.47892600
H	-3.86749100	-1.41970400	-0.92296700
H	-3.82221900	-2.05983500	0.72770900
H	3.11274800	1.45525600	1.58665300
H	4.02860700	2.41795400	0.40105100
H	4.71699700	0.01312600	0.34011300
H	4.02323800	0.57115900	-1.19108600
H	-1.66662300	-3.79924900	0.12224100
H	-2.58626500	-3.29490000	-1.30785500

H	-0.83222000	-3.07040500	-1.26244000
H	-2.49768200	2.71725200	-1.24771800
H	-4.11560500	2.17560100	-0.80850200
H	-3.07413100	2.91494600	0.42160700
H	2.46951700	-2.65657200	-0.77797900
H	4.14818600	-2.15192600	-0.65364700
H	3.13439000	-1.51450500	-1.96698900
O	2.10400300	2.04642400	-0.11424300

Cartesian coordinates of the optimized geometry of N_3O_1 (S_0, axial) using PBE0-GD3BJ/TZVP with the PCM method (solvent = dimethyl sulfoxide).

C	-3.52070800	0.10392400	0.47599000
C	-3.30711800	-1.14845200	-0.35649700
N	-2.53238900	1.10954700	0.11723700
C	-1.23497700	0.61073800	0.02256300
C	-0.97295000	-0.77290900	-0.03436800
N	-2.00021200	-1.71664200	-0.06675100
C	-0.13535900	1.49214300	0.04988300
C	1.19012800	1.04754100	-0.01220500
C	1.46382800	-0.31927400	-0.11476700
C	0.36563500	-1.19478500	-0.15949800
O	2.15297000	1.98966800	0.03967600
N	2.77414500	-0.78357500	-0.25883100
C	3.48411600	1.49983700	0.21544200
C	3.69110700	0.27672200	-0.64729000
C	-2.03404100	-2.68677400	1.02259600
C	-2.98709900	1.99005500	-0.95386900
C	3.29097300	-1.64130700	0.80166200
C	0.61446500	-2.55032700	-0.50005700
N	0.81964600	-3.64038600	-0.81978500
C	-0.34359200	2.87515500	0.29032500
N	-0.50093300	3.99657200	0.51308800
H	-3.42420000	-0.13656500	1.53926300
H	-4.51369100	0.52525700	0.32092600
H	-3.36724500	-0.90041500	-1.42081000
H	-4.06605800	-1.90254600	-0.14906400
H	3.63918200	1.26229800	1.27307100
H	4.14210900	2.32054000	-0.06489800
H	4.71458700	-0.08573300	-0.55334700
H	3.52046800	0.53693700	-1.69571700
H	-1.03127400	-3.03058200	1.26509100
H	-2.62170800	-3.55243500	0.71345600
H	-2.48008100	-2.26320700	1.93287900
H	-3.82671400	2.58713500	-0.59508300
H	-3.30916900	1.42148600	-1.83685300
H	-2.19704400	2.67122600	-1.25940800
H	2.54749300	-2.38173200	1.08868600
H	3.57123200	-1.06978300	1.69714200
H	4.17010400	-2.17156600	0.43309000

Cartesian coordinates of the optimized geometry of N_4 (S_0, equatorial) using PBE0-GD3BJ/TZVP with the PCM method (solvent = dimethyl sulfoxide).

C	3.68552100	-0.50631000	0.16543900
C	3.49828000	0.77956900	-0.58080400

N	2.49373800	-1.33243800	0.08998200
C	1.28287700	-0.63261900	0.01127600
C	1.21596900	0.76392500	0.05030600
N	2.38029400	1.52462900	-0.01919500
C	0.07645800	-1.36795400	-0.14729800
C	-1.20917200	-0.79230800	-0.06901800
C	-1.27632900	0.61996900	-0.02804100
C	-0.07586300	1.35121500	0.13948900
N	-2.38642000	-1.52833000	-0.03691600
N	-2.47234800	1.28248900	-0.15388200
C	-3.50584800	-0.79290800	0.53549600
C	-3.69437300	0.51154300	-0.17921400
C	2.29569400	2.87296400	-0.55903600
C	2.57813500	-2.39152800	1.08929400
C	-2.56280100	2.55295700	-0.84801700
C	-2.33861500	-2.90880500	0.41653200
C	-0.21916500	2.64623100	0.69032500
N	-0.37091000	3.65500200	1.23562200
C	0.23074000	-2.67725800	-0.66870000
N	0.39005500	-3.69984600	-1.18371800
H	3.92451900	-0.27496500	1.21438300
H	4.52885700	-1.06440400	-0.25067900
H	3.33366700	0.58058400	-1.65095200
H	4.40089100	1.38223500	-0.48306400
H	-3.34654200	-0.61798400	1.61019800
H	-4.40836800	-1.39162800	0.42038200
H	-4.49018200	1.08350500	0.30731600
H	-4.00293500	0.32524100	-1.21709500
H	1.92884300	3.58660300	0.17480000
H	3.29825000	3.18433800	-0.84797600
H	1.64971800	2.91882600	-1.44638900
H	1.74702000	-3.08684200	1.02566500
H	3.49493100	-2.95744300	0.91459200
H	2.61563800	-1.97508100	2.10636400
H	-1.63483000	2.76678600	-1.37964300
H	-2.77106900	3.38484300	-0.17076800
H	-3.36939200	2.49302500	-1.58398900
H	-2.00595000	-3.58562600	-0.36703200
H	-1.67828400	-3.02838100	1.28598000
H	-3.34470700	-3.20628200	0.70648000

Cartesian coordinates of the optimized geometry of N_4 (S_0, axial) using PBE0-GD3BJ/TZVP with the PCM method (solvent = dimethyl sulfoxide).

C	-3.59437500	-0.61705000	-0.44297100
C	-3.59437200	0.61705100	0.44297100
N	-2.42573600	-1.43271400	-0.15235900
C	-1.24178800	-0.70148800	-0.05446400
C	-1.24178700	0.70148800	0.05446200
N	-2.42573500	1.43271400	0.15235600
C	0.00000000	-1.35902900	-0.14762800
C	1.24178700	-0.70148800	-0.05446500
C	1.24178800	0.70148800	0.05446200
C	0.00000100	1.35902900	0.14762600
N	2.42573600	1.43271100	0.15235800
C	3.59437300	-0.61705100	-0.44297500
C	3.59437400	0.61704700	0.44297000

C	-2.67564900	2.42580300	-0.88672400
C	-2.67564900	-2.42580100	0.88672400
C	2.67565200	2.42581100	-0.88670900
C	-0.00000100	2.72235000	0.54380100
N	-0.00000400	3.81638100	0.91339700
C	-0.00000100	-2.72235100	-0.54380100
N	-0.00000100	-3.81638400	-0.91339000
H	-3.57583600	-0.31722300	-1.49546600
H	-4.48668200	-1.22326400	-0.28748200
H	-3.57582800	0.31722200	1.49546600
H	-4.48668000	1.22326500	0.28748800
H	3.57583300	-0.31722200	-1.49547000
H	4.48668000	-1.22326700	-0.28748900
H	4.48668200	1.22326200	0.28748400
H	3.57583600	0.31721700	1.49546400
H	-1.76137100	2.95485900	-1.14439500
H	-3.39650100	3.15748400	-0.51859700
H	-3.07455800	1.96619100	-1.80143900
H	-3.39649500	-3.15748700	0.51859500
H	-3.07456600	-1.96618700	1.80143500
H	-1.76137000	-2.95485000	1.14440100
H	1.76137200	2.95485700	-1.14439100
H	3.07458100	1.96621000	-1.80142100
H	3.39649000	3.15749900	-0.51856600
N	2.42573400	-1.43271500	-0.15236200
C	2.67564800	-2.42580500	0.88671800
H	3.39648800	-3.15749500	0.51858400
H	3.07457300	-1.96619500	1.80142700
H	1.76136800	-2.95485000	1.14440000

NATURAL TRANSITION ORBITALS AND ELECTRON DENSITY DIFFERENCES

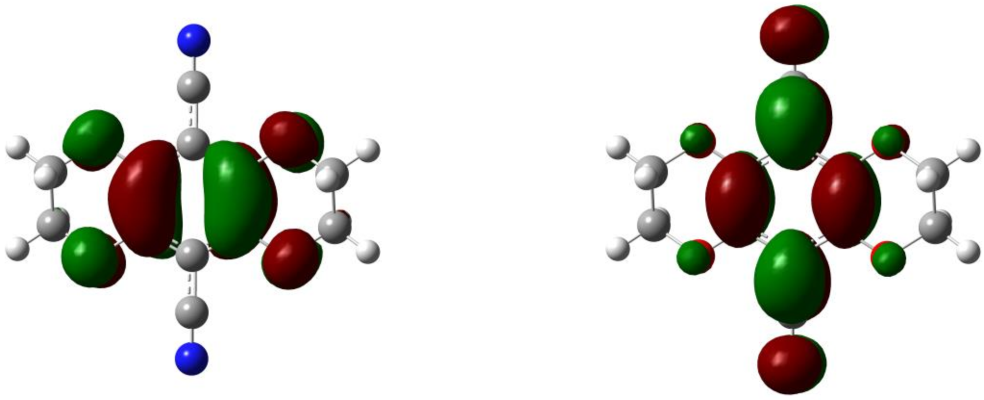

Figure S65: Occupied (left) and virtual (right) natural transition orbitals of the optimized S_0 state of **O4** calculated by means of PBE0/TZVP (isovalue = 0.030 au).

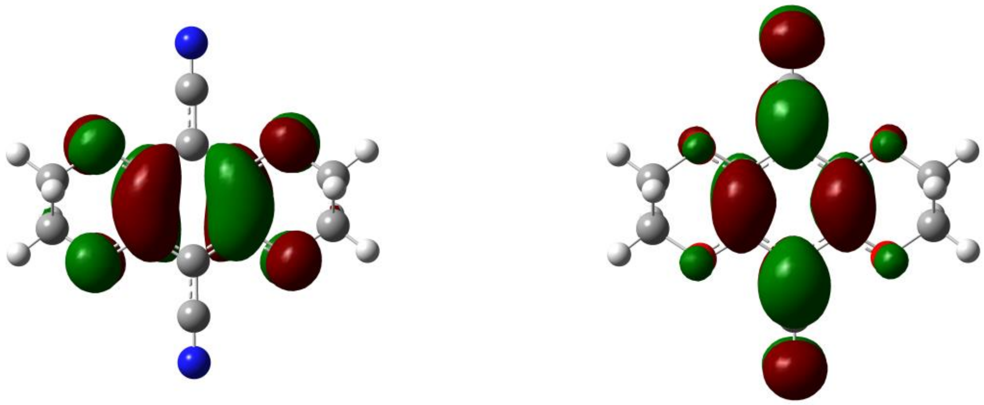

Figure S66: Occupied (left) and virtual (right) natural transition orbitals of the optimized S_1 state of **O4** calculated by means of PBE0/TZVP (isovalue = 0.030 au).

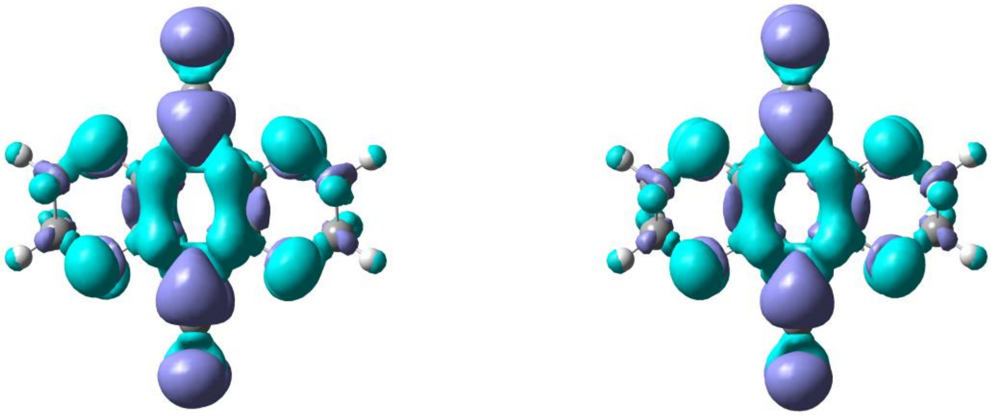

Figure S67: Electron density difference between excited states and ground states computed using PBE0/TZVP (isovalue = 0.0008 au). The violet (cyan) area represents the region where the electron density is increased (reduced) upon the absorption. Left: Electron density difference at the optimized S_0 state of **O4**. Right: Electron density difference at the optimized S_1 state of **O4**.

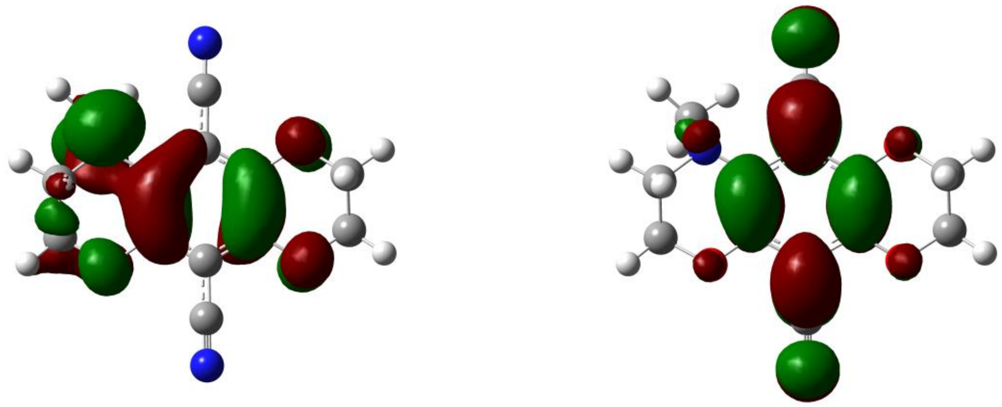

Figure S68: Occupied (left) and virtual (right) natural transition orbitals of the optimized S_0 state of **N_1O_3** (all-axial) calculated by means of PBE0/TZVP (isovalue = 0.030 au).

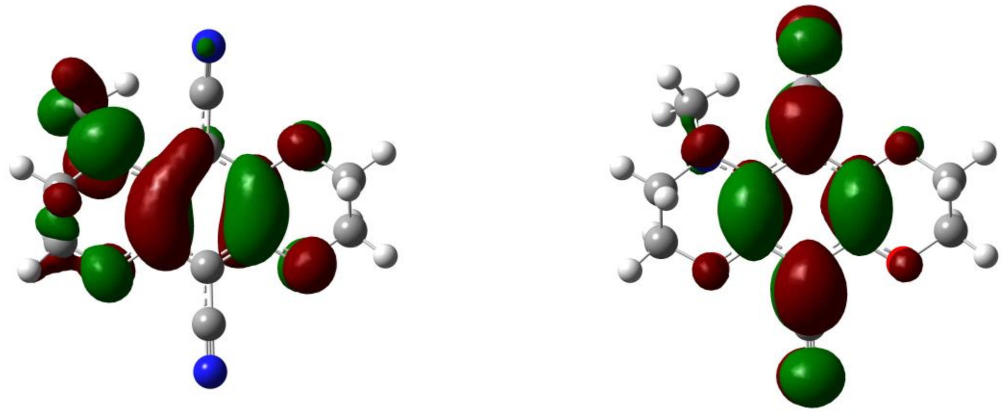

Figure S69: Occupied (left) and virtual (right) natural transition orbitals of the optimized S_1 state of **N_1O_3** (all-axial) calculated by means of PBE0/TZVP (isovalue = 0.030 au).

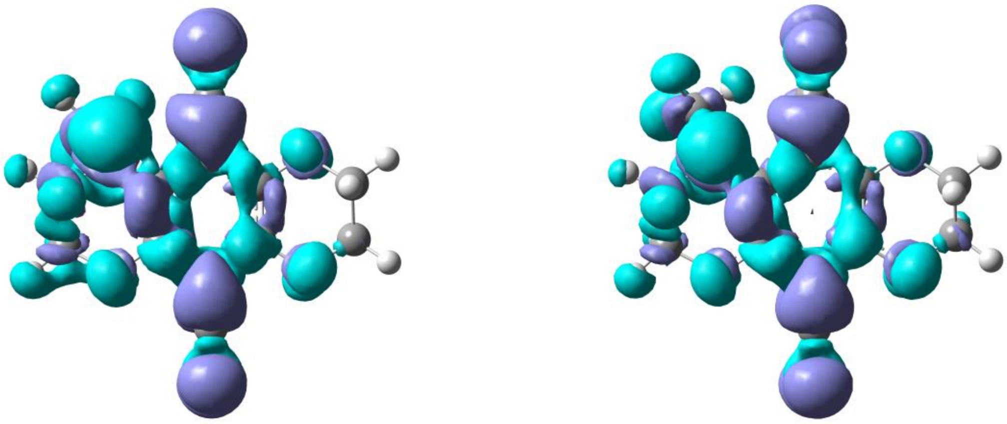

Figure S70: Electron density difference between excited states and ground states computed using PBE0/TZVP (isovalue = 0.0008 au). The violet (cyan) area represents the region where the electron density is increased (reduced) upon the absorption. Left: Electron density difference at the optimized S_0 state of **N_1O_3**. Right: Electron density difference at the optimized S_1 state of **N_1O_3** (all-axial).

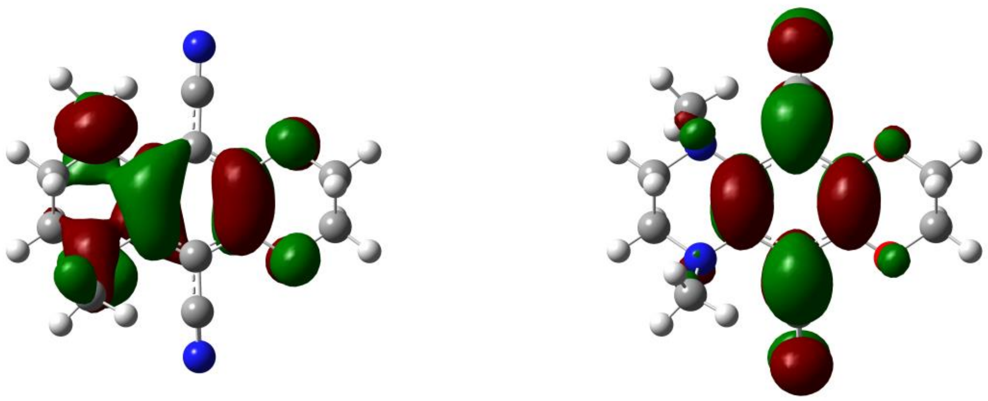

Figure S71: Occupied (left) and virtual (right) natural transition orbitals of the optimized S_0 state of **N_2O_2** (all-axial) calculated by means of PBE0/TZVP (isovalue = 0.030 au).

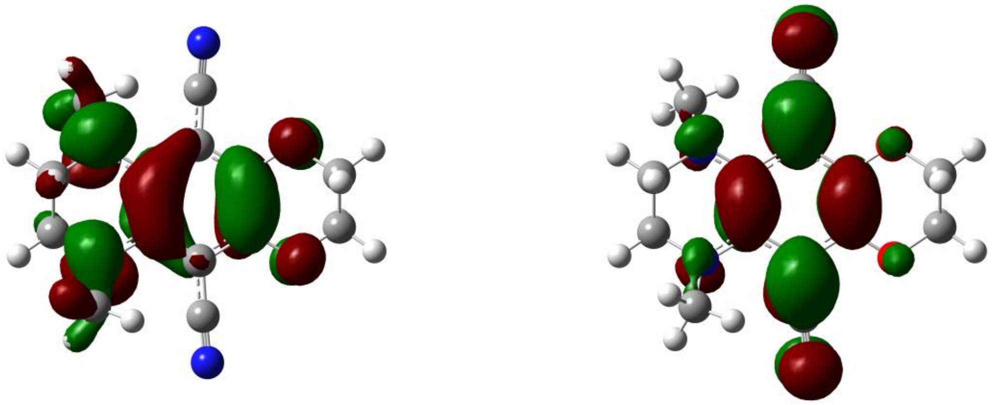

Figure S72: Occupied (left) and virtual (right) natural transition orbitals of the optimized S_1 state of **N_2O_2** (all-axial) calculated by means of PBE0/TZVP (isovalue = 0.030 au).

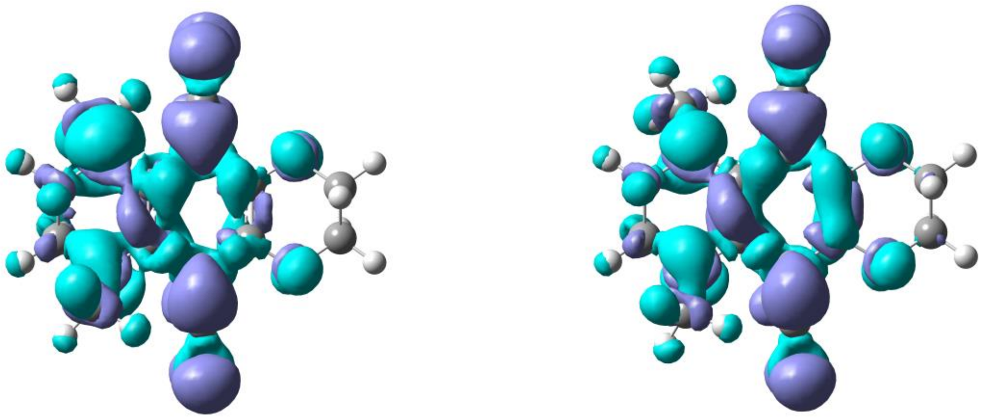

Figure S73: Electron density difference between excited states and ground states computed using PBE0/TZVP (isovalue = 0.0008 au). The violet (cyan) area represents the region where the electron density is increased (reduced) upon the absorption. Left: Electron density difference at the optimized S_0 state of **N_2O_2**. Right: Electron density difference at the optimized S_1 state of **N_2O_2** (all-axial).

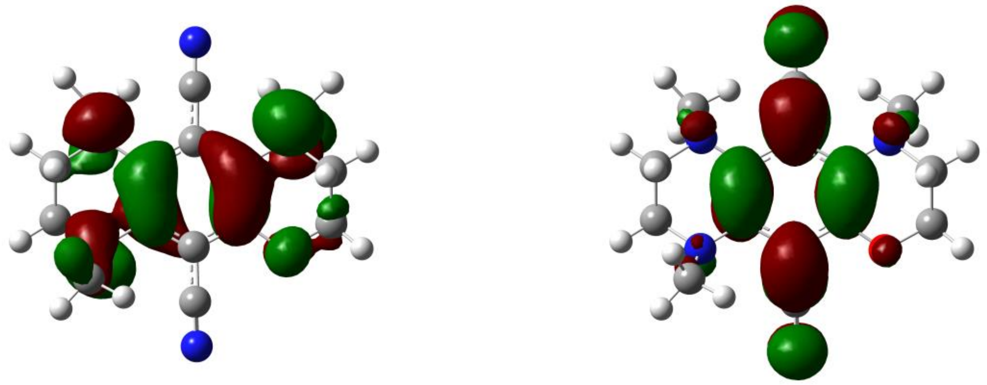

Figure S74: Occupied (left) and virtual (right) natural transition orbitals of the optimized S_0 state of **N_3O_1** (all-axial) calculated by means of PBE0/TZVP (isovalue = 0.030 au).

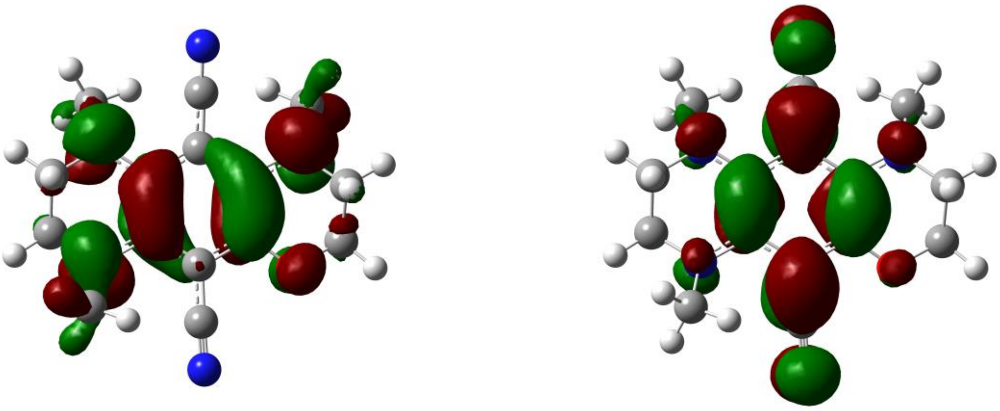

Figure S75: Occupied (left) and virtual (right) natural transition orbitals of the optimized S_1 state of **N_3O_1** (all-axial) calculated by means of PBE0/TZVP (isovalue = 0.030 au).

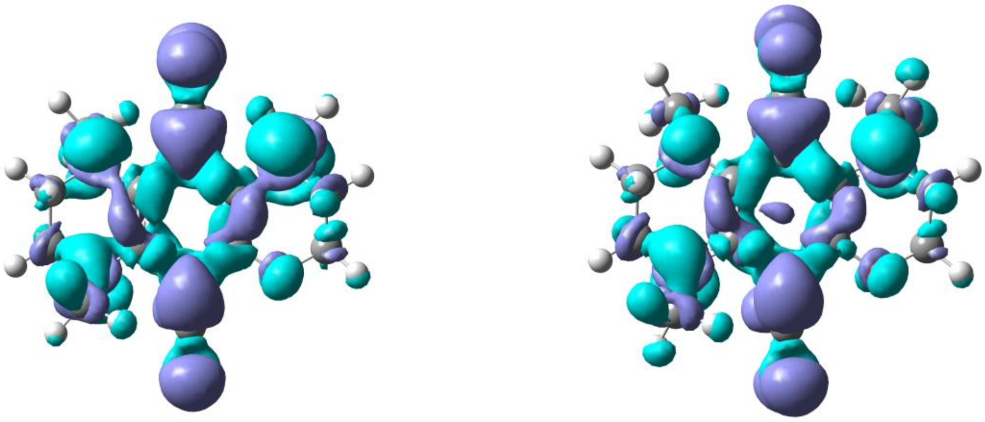

Figure S76: Electron density difference between excited states and ground states computed using PBE0/TZVP (isovalue = 0.0008 au). The violet (cyan) area represents the region where the electron density is increased (reduced) upon the absorption. Left: Electron density difference at the optimized S_0 state of **N_3O_1**. Right: Electron density difference at the optimized S_1 state of **N_3O_1** (all-axial).

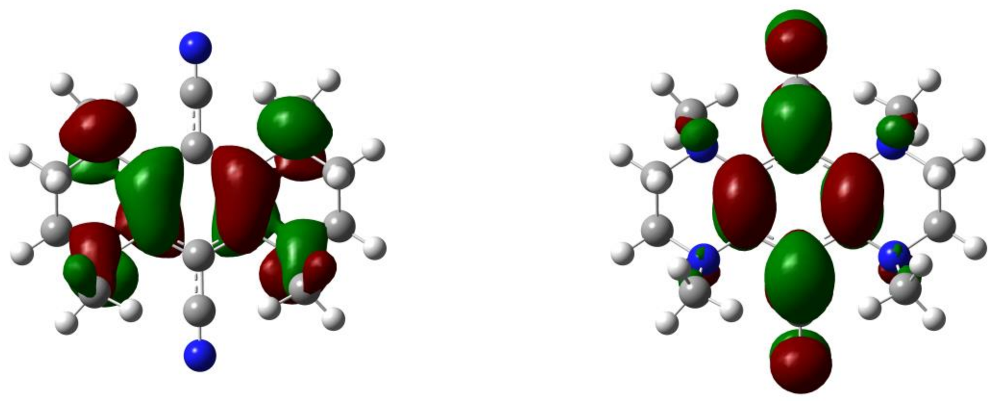

Figure S77: Occupied (left) and virtual (right) natural transition orbitals of the optimized S_0 state of **N_4** (all-axial) calculated by means of PBE0/TZVP (isovalue = 0.030 au).

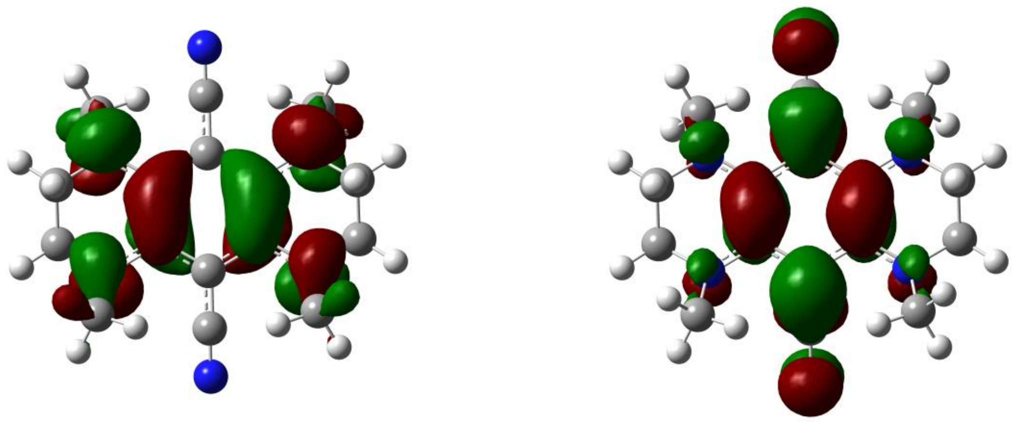

Figure S78: Occupied (left) and virtual (right) natural transition orbitals of the optimized S_1 state of **N_4** (all-axial) calculated by means of PBE0/TZVP (isovalue = 0.030 au).

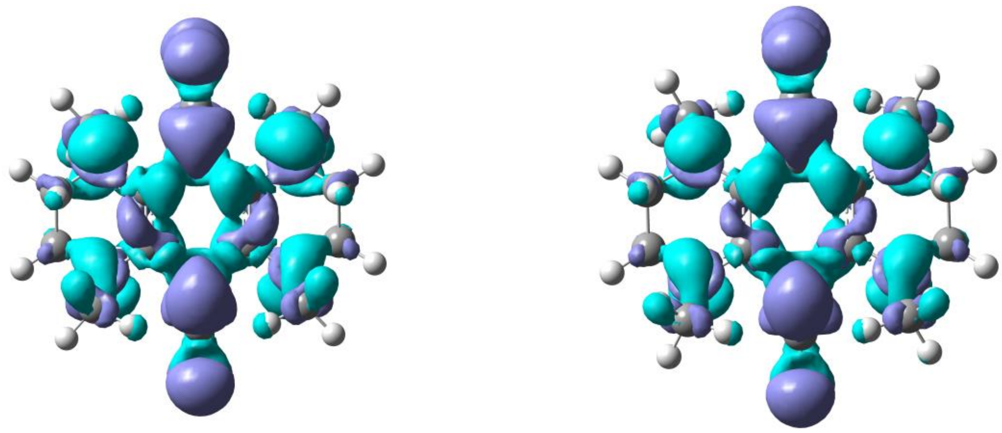

Figure S79: Electron density difference between excited states and ground states computed using PBE0/TZVP (isovalue = 0.0008 au). The violet (cyan) area represents the region where the electron density is increased (reduced) upon the absorption. Left: Electron density difference at the optimized S_0 state of **N_4**. Right: Electron density difference at the optimized S_1 state of **N_4** (all-axial).

7 CELL ASSAYS AND MICROSCOPY

Cell culture

The human HeLa Kyoto cell line (RRID:CVCL_1922) was cultivated in DMEM growth medium (*Invitrogen*) with 10% FBS (*Gibco*), 1% Antibiotic-Antimycotic (*Gibco*) at 37 °C, 95% relative humidity and 5% CO_2.

Laser confocal microscopy

HeLa Kyoto cells were seeded in microscopy dishes (µ-Slide 8 Well, *Ibidi*) at a concentration of $4x10^4$ cells per well. After 24 h, cells were either treated for 24 h by directly adding the compounds solved in DMSO to culture medium, preincubated with 1 µL of Lipofectamine™ 2000 (Invitrogen) for 10 min prior to compound treatment or by directly adding the compounds solved in Pluronic® F-127 (*Thermo Fisher Scientific*). After fixation (4% PFA in PBS pH 7.4, 15 min, RT) and permeabilization (0.3% Triton in PBS pH 7.4, 15 min, RT), cells were stained with HCS CellMask® Deep Red (*Thermo Fisher Scientific*, 1:1000 in DPBS, 30 min, RT). Imaging was performed on a Leica TCS SP8X Falcon Confocal Laser Scanning Microscope using the Leica Application Suite X (LAS X) software (*Leica Microsystems*). The laser confocal microscope is equipped with two continuous wave lasers (diode: 405 nm; argon: 458/476/488/496/514 nm) and one pulsed white light laser (WLL) tunable from 470-670 nm. It also contains a 5-channel SP-detector consisting of 2 HyD SMD detectors, 1 HyD and 2 photomultiplier tubes (PMT). Imaging was performed with a HC PL APO 63x/1.2 W motCORR CS2 water objective. The samples were excited using the diode at 405 nm and the WLL at 633 nm and detected with two HyD hybrid photon counting detectors (Leica). The emission of HCS CellMask® Deep Red was detected at emission wavelengths between 650-720 nm. All compounds were excited at 405 nm before detecting the fluorescence signal over a large range (412-640 nm) to enable detection of each compound relying on their specific emission maxima. For an optimal visualization of all compounds irrespective of their unique emission properties, linear adjustment of contrast and brightness was applied to all sets of images equally.

Cell viability assay

HeLa Kyoto cells were seeded in 96-well plates (Greiner) at a concentration of $1x10^4$ cells per well and incubated at 37 °C, 95 % humidity and 5 % CO_2. After 24 h, cells were either treated for another 24 h by directly adding the compounds solved in DMSO to culture medium, preincubated with 1 µL of Lipofectamine™ 2000 (*Invitrogen*) for 10 min prior to compound treatment or by directly adding the compounds solved in Pluronic® F-127. Then, the CellTiter® Aqueous One Kit (*Promega*) was utilized according to the instructions of the manufacturer. Briefly, cells were incubated for 60 min before the absorption of the metabolite was measured at 490 nm using a Glomax®-Multi plate reader (*Promega*). Cell viability was then determined after blank subtraction followed by normalization to the negative control of each series.

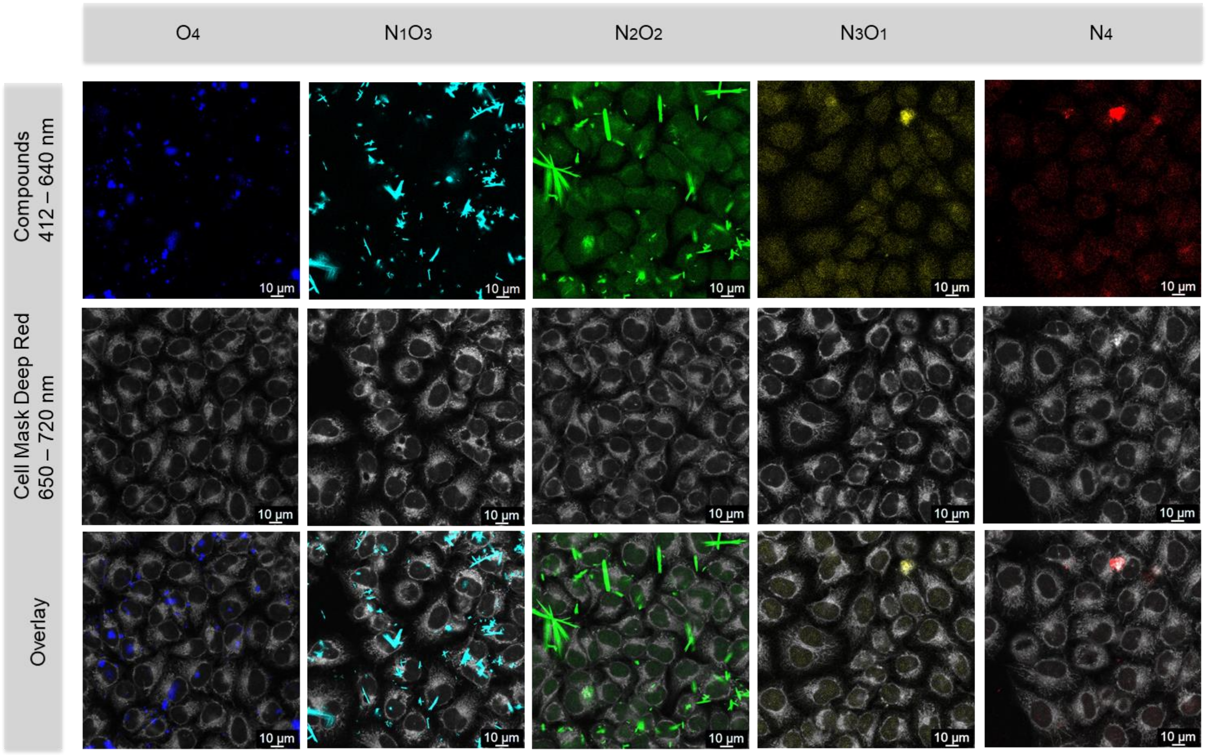

Figure S80: Internalization of compounds (100 µM in 100% DMSO) in HeLa Kyoto cells imaged by confocal laser scanning microscopy after 24 hours of treatment. Cells were imaged after fixation and staining with HCS CellMask™ Deep Red (shown in grey). Compounds are shown in the color of their actual emission wavelength. Contrast was adjusted by 20–40% to optimize the visibility of the compounds. Scale bar: 10 µm.

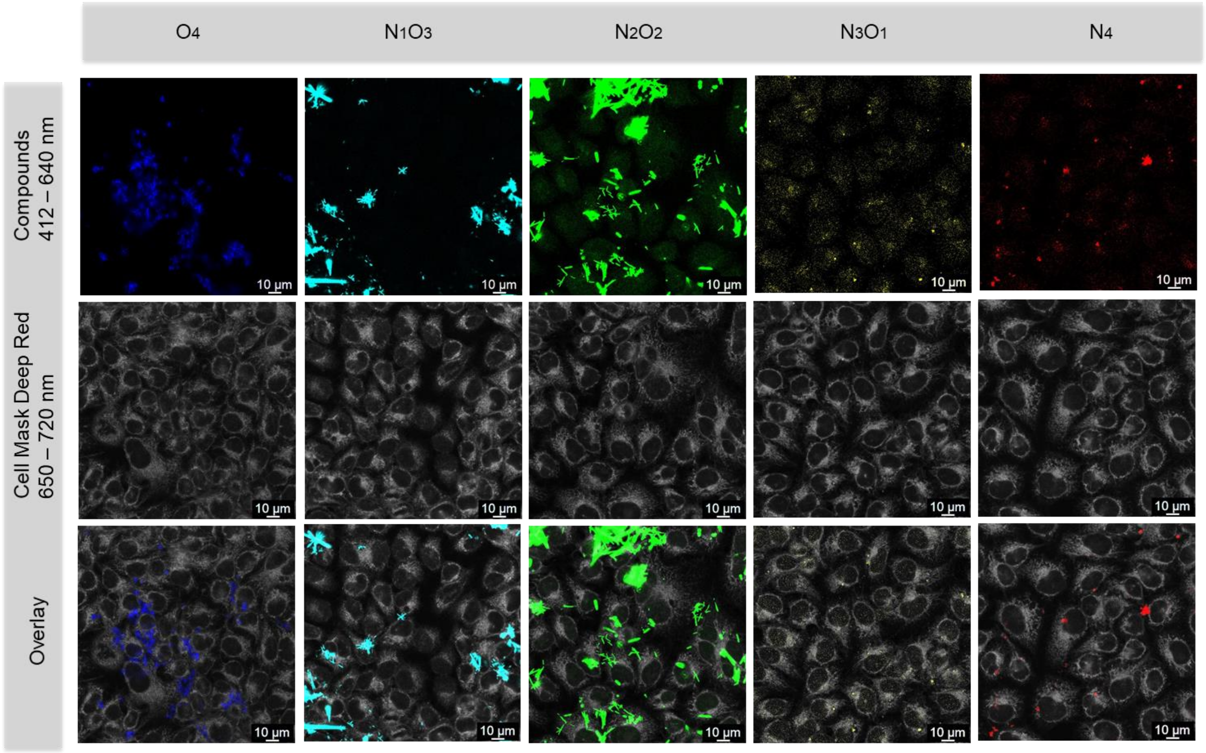

Figure S81: Internalization of Lipofectamine™ 2000 liposome-enclosed compounds (1 µL Lipofectamine™ 2000 added to 100 µM compounds in 100% DMSO) in HeLa Kyoto cells imaged by confocal laser scanning microscopy after 24 hours of treatment. Cells were imaged after fixation and staining with HCS CellMask™ Deep Red (shown in grey). Compounds in liposomes are shown in the color of their actual emission wavelength. Contrast was adjusted by 20–40% to optimize the visibility of the compounds. Scale bar: 10 µm.

8 LITERATURE

[1] G. M. Sheldrick, *Acta Crystallogr. Sect. A* **1990**, *46*, 467–473.
[2] G. M. Sheldrick, *Acta Crystallogr. Sect. C Struct. Chem.* **2015**, *71*, 3–8.
[3] Sheldrick, G. M., SHELXL-2017, Program for the Refinement of Crystal Structures, University of Göttingen, Göttingen (Germany), **2017**.
[4] C. B. Hübschle, G. M. Sheldrick, B. Dittrich, *J. Appl. Crystallogr.* **2011**, *44*, 1281–1284.
[5] M. J. Frisch, G. W. Trucks, H. B. Schlegel, G. E. Scuseria, M. A. Robb, J. R. Cheeseman, G. Scalmani, V. Barone, G. A., Petersson, H. Nakatsuji, X. Li, M. Caricato, A. V. Marenich, J. Bloino, B. G. Janesko, R. Gomperts, B. Mennucci, H. P., Hratchian, J. V. Ortiz, A. F. Izmaylov, J. L. Sonnenberg, D. Williams-Young, F. Ding, F. Lipparini, F. Egidi, J. Goings, B. Peng, A., Petrone, T. Henderson, D. Ranasinghe, V. G. Zakrzewski, J. Gao, N. Rega, G. Zheng, W. Liang, M. Hada, M. Ehara, K. Toyota, R. Fukuda, J. Hasegawa, M. Ishida, T. Nakajima, Y. Honda, O. Kitao, H. Nakai, T. Vreven, K. Throssell, J. A. Montgomery, Jr., J. E. Peralta, F. Ogliaro, M. J. Bearpark, J. J. Heyd, E. N. Brothers, K. N. Kudin, V. N. Staroverov, T. A. Keith, R. Kobayashi, J., Normand, K. Raghavachari, A. P. Rendell, J. C. Burant, S. S. Iyengar, J. Tomasi, M. Cossi, J. M. Millam, M. Klene, C. Adamo,, R. Cammi, J. W. Ochterski, R. L. Martin, K. Morokuma, O. Farkas, J. B. Foresman, D. J. Fox, *Gaussian 16, Revision A.03,* **2016**, Gaussian, Inc., Wallingford CT.
[6] J. P. Perdew, K. Burke, M. Ernzerhof, *Phys. Rev. Lett.* **1996**, *77*, 3865–3868.
[7] A. Schäfer, C. Huber, R. Ahlrichs, *J. Chem. Phys.* **1994**, *100*, 5829–5835.
[8] S. Grimme, S. Ehrlich, L. Goerigk, *J. Comput. Chem.* **2011**, *32*, 1456–1465.
[9] F. Furche, R. Ahlrichs, *J. Chem. Phys.* **2002**, *117*, 7433–7447.
[10] L. Wang, T. Chen, S. Chen, Q. Chen, M. He, *J. Heterocycl. Chem.* **2014**, *51*, 1536–1540.
[11] A. Ashok Phadte, A. Chattopadhyay, S. Banerjee, D. Singh Sisodiya, T. Raghava, *ChemistrySelect* **2020**, *5*, 10177–10186.
[12] T. Raghava, P. Bhavana, S. Banerjee, *J. Mol. Struct.* **2024**, *1318*, 139340.
[13] T. Raghava, A. Chattopadhyay, S. Banerjee, N. Sarkar, *Org. Biomol. Chem.* **2024**, *22*, 364–373.
[14] T. Gallavardin, M. Maurin, S. Marotte, T. Simon, A.-M. Gabudean, Y. Bretonnière, M. Lindgren, F. Lerouge, P. L. Baldeck, O. Stéphan, Y. Leverrier, J. Marvel, S. Parola, O. Maury, C. Andraud, *Photochem. Photobiol. Sci.* **2011**, *10*, 1216–1225.
[15] P. R. Spackman, M. J. Turner, J. J. McKinnon, S. K. Wolff, D. J. Grimwood, D. Jayatilaka, M. A. Spackman, *J. Appl. Crystallogr.* **2021**, *54*, 1006–1011.
